# Urolithin A analog inhibits castration-resistant prostate cancer by targeting the androgen receptor and its variant, androgen receptor-variant 7

**DOI:** 10.3389/fphar.2023.1137783

**Published:** 2023-03-03

**Authors:** Balaji Chandrasekaran, Ashish Tyagi, Uttara Saran, Venkatesh Kolluru, Becca V. Baby, Venkat R. Chirasani, Nikolay V. Dokholyan, Jyh M. Lin, Amandeep Singh, Arun K. Sharma, Murali K. Ankem, Chendil Damodaran

**Affiliations:** ^1^ Department of Pharmaceutical Science, College of Pharmacy, Texas A&M University, College Station, TX, United States; ^2^ Department of Urology, University of Louisville, Louisville, KY, United States; ^3^ Department of Pharmacology, Penn State Cancer Institute, Penn State College of Medicine, Hershey, PA, United States; ^4^ Department of Biochemistry and Molecular Biology, Penn State College of Medicine, Hershey, PA, United States

**Keywords:** CRPC, Androgen Receptor, AR-Splice Variants, N-terminal domain, small molecule, growth inhibition

## Abstract

We investigated the efficacy of a small molecule ASR-600, an analog of Urolithin A (Uro A), on blocking androgen receptor (AR) and its splice variant AR-variant 7 (AR-V7) signaling in castration-resistant prostate cancer (CRPC). ASR-600 effectively suppressed the growth of AR^+^ CRPC cells by inhibiting AR and AR-V7 expressions; no effect was seen in AR^−^ CRPC and normal prostate epithelial cells. Biomolecular interaction assays revealed ASR-600 binds to the N-terminal domain of AR, which was further confirmed by immunoblot and subcellular localization studies. Molecular studies suggested that ASR-600 promotes the ubiquitination of AR and AR-V7 resulting in the inhibition of AR signaling. Microsomal and plasma stability studies suggest that ASR-600 is stable, and its oral administration inhibits tumor growth in CRPC xenografted castrated and non-castrated mice. In conclusion, our data suggest that ASR-600 enhances AR ubiquitination in both AR^+^ and AR-V7 CRPC cells and inhibits their growth *in vitro* and *in vivo* models.

## Introduction

Androgen receptor (AR) signaling plays a key role in prostate cancer (CaP) pathogenesis (Schmidt and Tindall, 2013; Tan et al., 2015). Androgen deprivation therapy (ADT) that either represses androgen synthesis (van Poppel and Nilsson, 2008) or inhibits AR function (Tran et al., 2009) is the initial treatment for both localized and advanced CaP (Loblaw et al., 2007). Structurally, full-length AR (AR-FL) comprises three domains: The N-terminal activation domain (NTD), DNA-binding domain (DBD), and ligand binding domain (LBD) (Shafi et al., 2013). Current ADTs block the AR LBD directly with anti-androgens or indirectly with androgen biosynthesis inhibitors (Lu et al., 2015; Imamura and Sadar, 2016). Second-generation ADT agents such as abiraterone and enzalutamide are often recommended as first-line therapeutics for castration-resistant prostate cancer (CRPC) (Beer et al., 2014). While patients are initially responsive toward ADT, tumor relapse often occurs, leading to the development of CRPC (de Bono et al., 2011). A characteristic feature of CRPC is its continued reliance on AR signaling; however, the mechanisms underlying AR reactivation remain unclear.

The truncated AR protein encoded by AR splice variants (AR-Vs) lacks the LBD domain while retaining the transactivating NTD domain. This results in ligand-independent activation and resistance to ADTs (Moon et al., 2018). The AR variant 7 (AR-V7) is the most commonly expressed variant identified to date and contains an intact AR NTD and DBD, as well as a unique C-terminal of 16-amino acids in place of the LBD (Antonarakis et al., 2016). This variant, unlike the AR-FL, is continuously localized to the nucleus (Hu et al., 2009) and has been reported to play a key role in promoting CRPC progression and metastasis, as well as developing resistance to ADT and anti-androgens (Guo et al., 2009; Li et al., 2013; Antonarakis et al., 2014). Moreover, heterogeneity among AR-driven CRPC is extensive, and AR-Vs is known to heterodimerize with the AR-FL (Brady et al., 1999; O'Neill et al., 2015). Thus, combining existing therapeutics with an AR-Vs-targeting agent may be a viable approach to overcome the acquired resistance of CRPC.

Recently, more attention was paid to post-translational regulation of AR activation, either by ubiquitination or lysosome degradation. Currently, three ubiquitination sites have been identified: K845 and K847 at the ligand-binding C-terminal (Xu et al., 2009; Linn et al., 2012) and another site K311 in the NTD (McClurg et al., 2017). Similarly, several preclinical studies suggest that induction of the lysosome pathway (autophagy) promotes CaP progression, and inhibition of lysosomal signaling abrogates the CaP growth (Finkbeiner, 2020; Machado et al., 2021). It has been reported that the AR activation is modulated by post-translational modifications, including ubiquitination (van der Steen et al., 2013).

Urolithin (UroA), a dietary gut microbiota-derived metabolite of ellagic acid, has been shown to exert anti-cancer effects on many cancer types (Sanchez-Gonzalez et al., 2016; Gonzalez-Sarrias et al., 2017; Dahiya et al., 2018; Komatsu et al., 2018; Zhao et al., 2018; Totiger et al., 2019). Interestingly, UroA has been shown to inhibit the proliferation of both androgen-dependent (LNCaP) and -independent (DU-145) CaP cells (Komatsu et al., 2018). In our prior study, our group demonstrated that UroA effectively inhibits AR^+^ CRPC growth in both *in vitro and in vivo* models compared to AR^−^ CRPC growth (Dahiya et al., 2018). UroA was also found to inhibit AR signaling in the AR^+^ CRPC cells, Moreover, the reintroduction of AR expression in AR-null PC-3 cells sensitized them to subsequent UroA treatment. Based on these results, we hypothesized that UroA might be a promising lead compound for developing potent and target-specific small UroA analogs to treat CRPC by directly targeting AR and AR-Vs.

**FIGURE 1:**
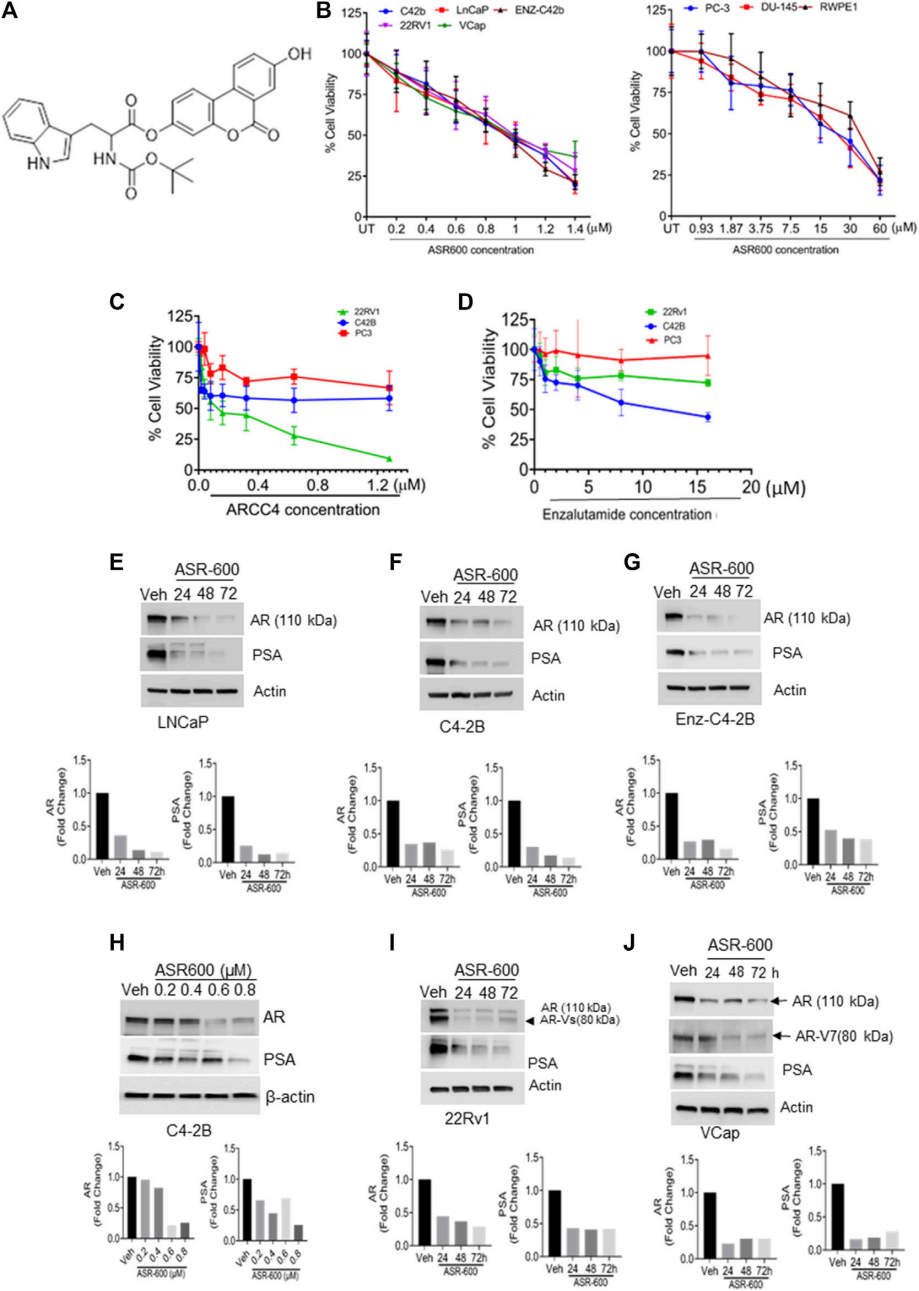
ASR-600 inhibits the AR and AR-V7 expression and abolishes the growth of AR^+^ CaP cells. **(A)**. The molecular structure of ASR-600. **(B)**. MTT cell viability assays performed on AR^+^ (C4-2B, LNCaP, EnZ-C4-2B, VCaP, and 22Rv1), AR- (PC-3 and DU-145) CaP, and normal prostate epithelial cell line (RWPE-1) with vehicle (DMSO) or different concentrations of ASR600. **(C, D)**. MTT cell viability assays were performed on AR^+^ C4-2B and 22Rv1, as well as AR- (PC-3) cells treated with vehicle (DMSO) or different concentrations of ARCC4 and Enzalutamide. **(E–G, I, J)** immunoblots for AR, AR-V7and PSA expressions following treated with vehicle (DMSO) or ASR-600 for 24, 48, and 72 h **(H)**. Immunoblots for AR and PSA expressions following treatment with vehicle (DMSO), ASR-600 (at indicated concentrations).

We synthesized several O-methylated amino acids (tryptophan) and sulfonamide-/sulfoxide-conjugated analogs of UroA and tested their therapeutic effect on CRPC cell lines. Through structure-activity relationship (SAR) studies, we identified ASR-600 (an N-Boc-protected tryptophan analog, Figure 1A) as the most potent analog that effectively targeted both AR-FL and AR-V7 and suppressed the growth of AR^+^ CaP and enzalutamide resistant cells in both *in vitro* and *in vivo* models.

## Methods

### Cell lines and reagents

Human CaP (LNCaP, PC-3, DU-145, VCaP, and 22Rv1), normal prostate epithelial (RWPE-1), and HEK-293T cell lines were purchased from the American Type Culture Collection (Manassas, VA, USA). C4-2B cells were obtained from ViroMed Laboratories (Minneapolis, MN, USA). The cells were grown on a specified medium, as described previously (Dahiya et al., 2018; Chandrasekaran et al., 2020). Dihydrotestosterone (DHT), cycloheximide (CHX), chloroquine (CQ), and MG132 were purchased from Sigma (St. Louis, MO). MG132 (10 µM) was used as the working concentration for experiments.

### Synthesis of ASR-600 (8-Hydroxy-6-oxo-6H-benzo [c]chromen-3-yl (tert butoxycarbonyl)tryptophanate)

ASR-600 was synthesized from Urolithin A according to a synthetic strategy recently developed in our laboratory. Briefly, Urolithin A (1.0 g, 4.38 mmol) was reacted with Nα-Boc-tryptophan (1.38 g, 4.38 mmol) in the presence of coupling agents 1-ethyl-3-(3-dimethyl aminopropyl) carbodiimide hydrochloride (0.67 g, 4.38 mmol), hydroxy benzotriazole (0.59 g, 4.48 mmol), and triethylamine (0.88 g, 8.76 mmol) in acetonitrile at room temperature to afford ASR-600 as the major product. The compound was purified by silica gel column chromatography to yield ASR-600 as an off-white solid and characterized by proton nuclear magnetic resonance (^1^H NMR, Bruker Avance 500 MHz)) and mass spectrometry (MS, Expression-S Compact Mass Spectrometer) and its purity (≥98%) was determined by high-performance liquid chromatography (HPLC). ^1^H NMR (500 MHz, DMSO-*d*_*6*_), 1.40 (s, 9H, 3 x CH_3_), 3.17–3.29 (m, 2H, CH_2_), 4.45–4.49 (m, 1H, CH), 6.77–6.85 (m, 1H, Ar-H), 6.85–6.87 (m, 1H, Ar-H), 7.01 (t, *J* = 10 Hz, 1H, Ar-H), 7.10 (t, *J* = 7.5 Hz, 1H, Ar-H), 7.37–7.41 (m, 1H, Ar-H), 7.57–7.61 (m, 2H, Ar-H), 7.75–7.76 (m, 1H, Ar-H), 8.15 (d, *J* = 9.0 Hz, 1H, Ar-H), 8.26 (d, *J* = 9.0 Hz, 1H, Ar-H), 8.30 (d, *J* = 8.5 Hz, 1H, Ar-H), 10.38 (s, 1H, OH), 10.93 (s, 1H, NH). MS *m/z* 457.92 [M^+^ (514.17) - *t*-butyl]. Melting point, 200°C–202°C.

### Cell viability assays

RWPE-1 and CaP cell lines were treated with vehicle control (DMSO) or different ASR-600 concentrations (200 nM–30 µM) for 24 h and then subjected to cell viability (MTT) assays as described before (Dahiya et al., 2018).

### Immunoblot and immunoprecipitation

Cell lysates (vehicle, ASR-600, DHT, DHT + ASR-600, AR transfected cells + ASR-600, empty vector-transfected cells + ASR-600) of CaP cells were prepared following treatment for 24 h in a 6-well plate. Immunoblots were performed as described previously (Dahiya et al., 2018) for the following antibodies: AR-V7 (abcam #ab198394), PSA (abcam#53774), PTEN (abcam# 31392), AR-FL (CST #5153), AKT (CST#4691), pAKT^ser473^ (CST #4060), pmTOR^Ser2481^(CST #5536), mTOR (CST #2972), ERα (CST#8644), PR (CST#3176), Ubiquitin (CST#3933), and Lamin A. β-Actin was used as the loading control (more details are given in Supplementary File S1). For immunoprecipitation (IP) experiments, protein samples were immunoprecipitated with the AR antibody per the protocol described before (Chandrasekaran et al., 2020). Briefly, in immunoprecipitation experiments, protein samples (40 µg) were extracted from cells using radioimmunoprecipitation assay (RIPA) buffer, then immunoprecipitated with AR antibody at 4°C under agitation overnight. The immunoprecipitated protein was pulled down using protein A-agarose beads (Thermo Fisher Scientific, Rockford, IL) at 4°C under rotary agitation for 3 h. Subsequently, centrifugation was followed by resuspension of the pellets in sample buffer, which was then heated for 5 min at 95°C for sodium dodecyl sulfate-polyacrylamide gel electrophoresis and immunoblot analysis for ubiquitin expression. All uncropped immunoblot images are presented as Supplementary File S3.

### Transfection

PC-3 and HEK-29 cells were seeded on 6-well plates in a respective medium supplemented with 10%FBS and were allowed to attach overnight. They were then transfected with either a control vector or AF-FL plasmid, using Lipofectamine-2000 reagent in Opti-MEM medium. After 24 h, these cells were treated with ASR-600 at different time points. Finally, the cells were lysed, protein was extracted, and AR expression was assessed using immunoblot and IP. Briefly, the transfected cells were treated with IC_50_ concentrations of ASR-600 and AR expression was assessed using immunoblots and IP.

### Real-time quantitative PCR

RNA was extracted from control and ASR600-treated cells using the RNAeasy Mini Kit (Qiagen, Hilden, Germany). This was followed by reverse transcription using the iScript DNA Synthesis Kit (Bio-Rad, Hercules, CA). RT-PCR using SYBR Green PCR Master Mix (Applied Biosystems, Foster City, CA) with specific AR and PSA primers as per the protocol described previously (Dahiya et al., 2018). β-actin was used as the internal control.

### Immunofluorescence and immunohistochemistry analysis

Immunofluorescence analysis for AR and AR-V7 was performed on control, ASR-600, DHT or ASR-600 + DHT, MG132 or MG132+ASR-600 treated cells as described previously (Dahiya et al., 2018). Immunohistochemical analyses were performed for Ki-67, AR and PSA expression on vehicle control and ASR-600 treated xenograft tumors (22RV1 and C2-4B).

### Proteasome activity

Proteasomal activity of control and ASR-600 treated C4-2B cells was measured using a Proteasome activity assay kit (BioVision) per the manufacturer’s protocol. MG132 was used as the positive control.

### Molecular docking

Molecular docking of ASR-600 to AR-NTD was conducted using MedusaDock 30, 32, 37, a docking program that incorporates structural flexibility during simultaneous sampling of conformational states of protein and ligand (Wang and Dokholyan, 2019). Due to the presence of low-complexity regions, the structural conformation of NTD is unknown. Therefore, we performed *ab initio* modeling to decipher its 3D structure. We submitted the FASTA sequence of NTD to the I-TASSER (Iterative Threading ASSEmbly Refinement) server (Yang et al., 2015), which utilizes a hierarchical approach to predict the structure of the query protein. Steric clashes were most prevalent in modeled and redesigned structures. Subsequently, we performed discrete molecular dynamics (DMD) simulations (Dokholyan et al., 1998; Lazaridis and Karplus, 2000; Shirvanyants et al., 2012; Ding and Dokholyan, 2013) for 6 × 10^6^-time steps to remove steric clashes and to optimize the energy of the modeled structure. The structures were clustered using Gromacs tools (Cherinka et al., 2018), and the optimal representative structure of NTD was extracted for subsequent docking studies with ASR-600. The optimized structure of ASR-600 was built using a Marvin sketch workspace (Cherinka et al., 2018).

### Nuclear magnetic resonance (NMR)

The proton NMR experiment was carried out with Bruker Avance 600 MHz NEO NMR equipped with the TCI cyroprobe. The purified NTD protein (5 μM/L) was mixed with ASR-600 (500 μM/L; dissolved in DMSO-*d*_*6*_) in 500 µL of 20 mM phosphate buffer made up of 100% deuterium oxide. The NMR saturation transfer difference (STD) experiment was carried out with a standard pulse program from the Bruker pulse library. Two parallel experiments: the reference (A, without saturation, blue spectra; with decoupler set at δ= −30 ppm) and one with selective saturation on the AR protein (B, red spectra; decoupler set at δ= 0.8 ppm) were executed. The peak intensity differences are shown by spectra C (green spectrum). The binding affinity was measured based on the peak intensity by the different spectra.

### Xenograft studies

The *in vivo* effect of ASR-600 was evaluated by subcutaneously injecting castrated and non-castrated 6–8 weeks old BALB/c male athymic nude mice (nu/nu), purchased from the Jackson Laboratory (Bar Harbor, ME, USA), with either 22Rv1 or C4-2B cells. ASR-600 first dissolved in DMSO was diluted in PBS to make a 0.1% solution. Mice bearing 22Rv1 and C4-2B xenografts were then randomized into control (placebo) and treatment (20 mg/kg, ASR-600) groups. All mice were euthanized *via* CO_2_ asphyxiation after 4 weeks of treatment, and the xenograft tumors were removed and fixed in 10% formalin for histopathological studies. All experimental animals were approved by the University of Louisville’s ethical committee and maintained following Institutional Animal Care and Use Committee approved-protocols.

### Microsomal incubation and sample preparation for liquid chromatography–mass spectrometry (LC-MS)

Pooled mouse liver microsomes were procured from BioIVT (USA). The ASR-600 metabolic stability was analyzed using previously established protocol (Attwa et al., 2020). A detailed procedure is given in the Supplementary Material. Briefly, 7.5 µL compound working solution was incubated with 592.5 μL microsomal/S9 mixture/vial and the metabolic reactions were initiated using NADPH (1 mM) for a specific time. Reactions were stopped at 14, 28, and 42 min by adding acetonitrile (ACN) containing 100 ng/mL tolbutamide. From the generated data after sample analysis, ASR-600 metabolic stability curve was established, and the *in vitro* intrinsic clearance was calculated (detailed procedure was given in Supplementary File S2).

### Statistical analysis

All statistical analyses were performed using GraphPad Prism 8.0a software (GraphPad Software, Inc., La Jolla, CA). Unpaired two-tailed Student’s *t*-tests and one-way ANOVA analysis were performed for two-group and multiple group comparisons, respectively. *p*-values <0.05 were considered statistically significant and values were presented as either mean ± SD.

## Results

### ASR-600 inhibits the growth of CaP cells

To develop more potent and target-specific compounds based on the lead UroA structure, a series of UroA analogs such as *O*-methylated and amino acid- and sulfonamide-/sulfoxide-conjugated analogs were synthesized. SAR studies based on cytotoxicity towards CaP cells led to the identification of ASR-600 (Figure 1A), an N-Boc-protected tryptophan analog, as the most potent compound. The inhibitory effect of ASR-600 on CaP cell growth in well-characterized human CaP cell lines (AR^+^: LNCaP, C4-2B; AR-Variant: 22Rv1, VCaP; AR-null: PC-3 and DU-145) and normal prostate epithelial cells (RWPE1) was assessed by MTT assay. ASR-600 treatment inhibited the viability of C4-2B (IC_50_: 824 nM), LnCaP (811 nM), 22Rv1 (IC_50_: 919 nM) and VCaP (IC_50_: 923 nM) cells in a concentration-dependent manner at 24 h. Similarly, treatment with ASR-600 for 24 h also significantly inhibited the viability of enzalutamide resistant C4-2B cells (IC_50_: 815 nM). However, IC_50_ of AR-null CaP cell lines PC-3 (18 µM) and DU-145 (19.5 µM) are higher than AR-null CRPC cells. Interestingly, the RWPE1 cells remained unaffected (IC_50_: 37 µM) with ASR-600 treatment (Figure 1B). We used AR inhibitors/degraders, such as enzalutamide and ARCC4, as controls for our experiments. The inhibitory effect of ASR-600 was significantly higher as compared to enzalutamide. ARCC4 was effective in 22RV1 (AR-FL/AR-V7) cells compared to ASR-600 (Figures 1C, D). These results suggested that AR^+^ CaP cell lines are sensitive to ASR-600 compared to AR-null CaP or the RWPE1 cells.

### ASR-600 targets AR and AR-V7 expression in CaP cell lines

**FIGURE 2:**
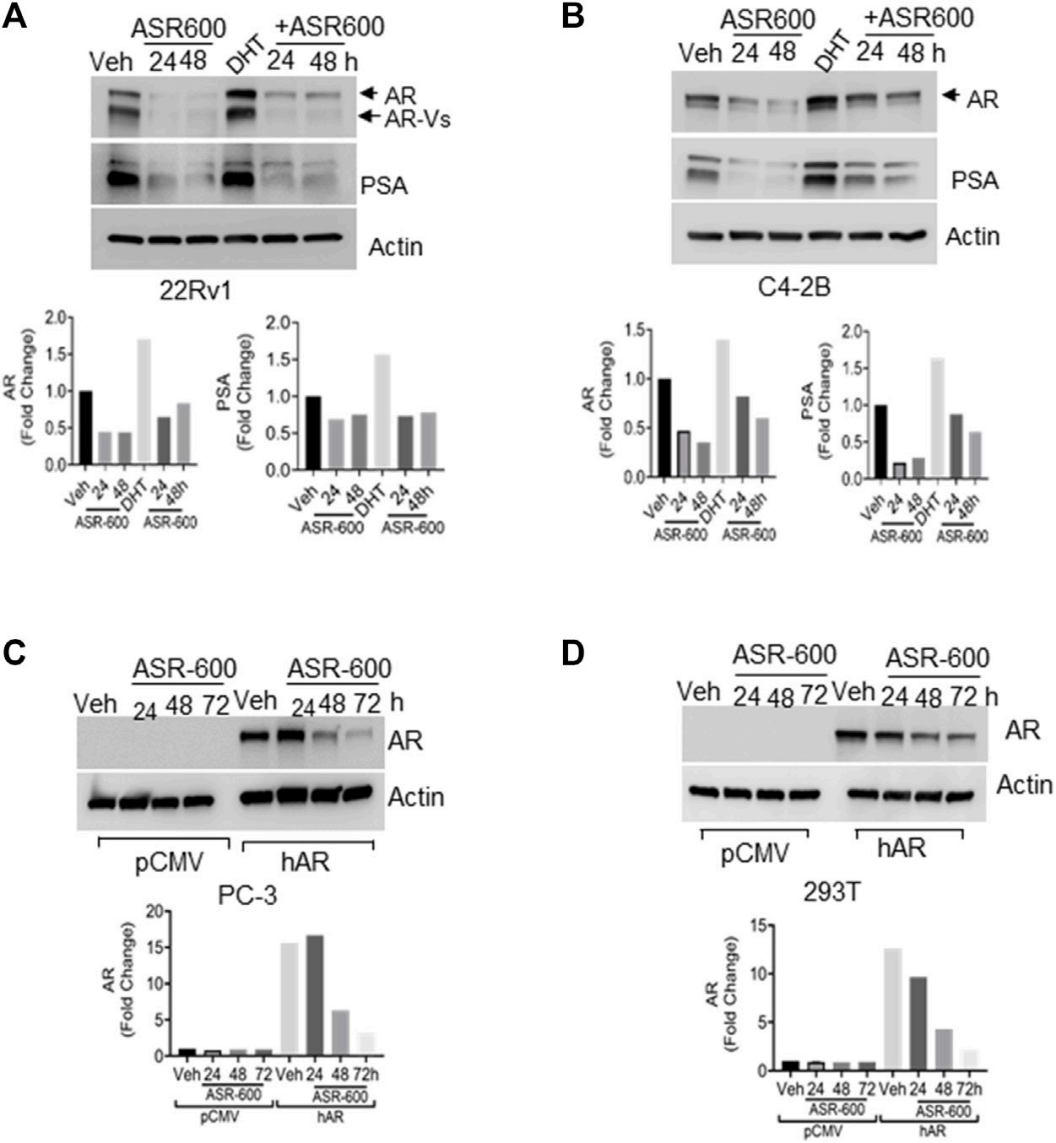
ASR-600 inhibits DHT-induced AR/AR-V7 expression. **(A, B)**. Immunoblots for AR, AR-V7and PSA expressions following treated with vehicle (DMSO) or ASR-600, DHT or DHT+ASR600 for 24 and 48 h in 22Rv1 and C4-2B cells. **(C, D)** Immunoblots for AR-expression in empty vector or AR transfected PC-3 or 293T cells following ASR-600 treatment for 24, 48, and 72 h.

**FIGURE 3:**
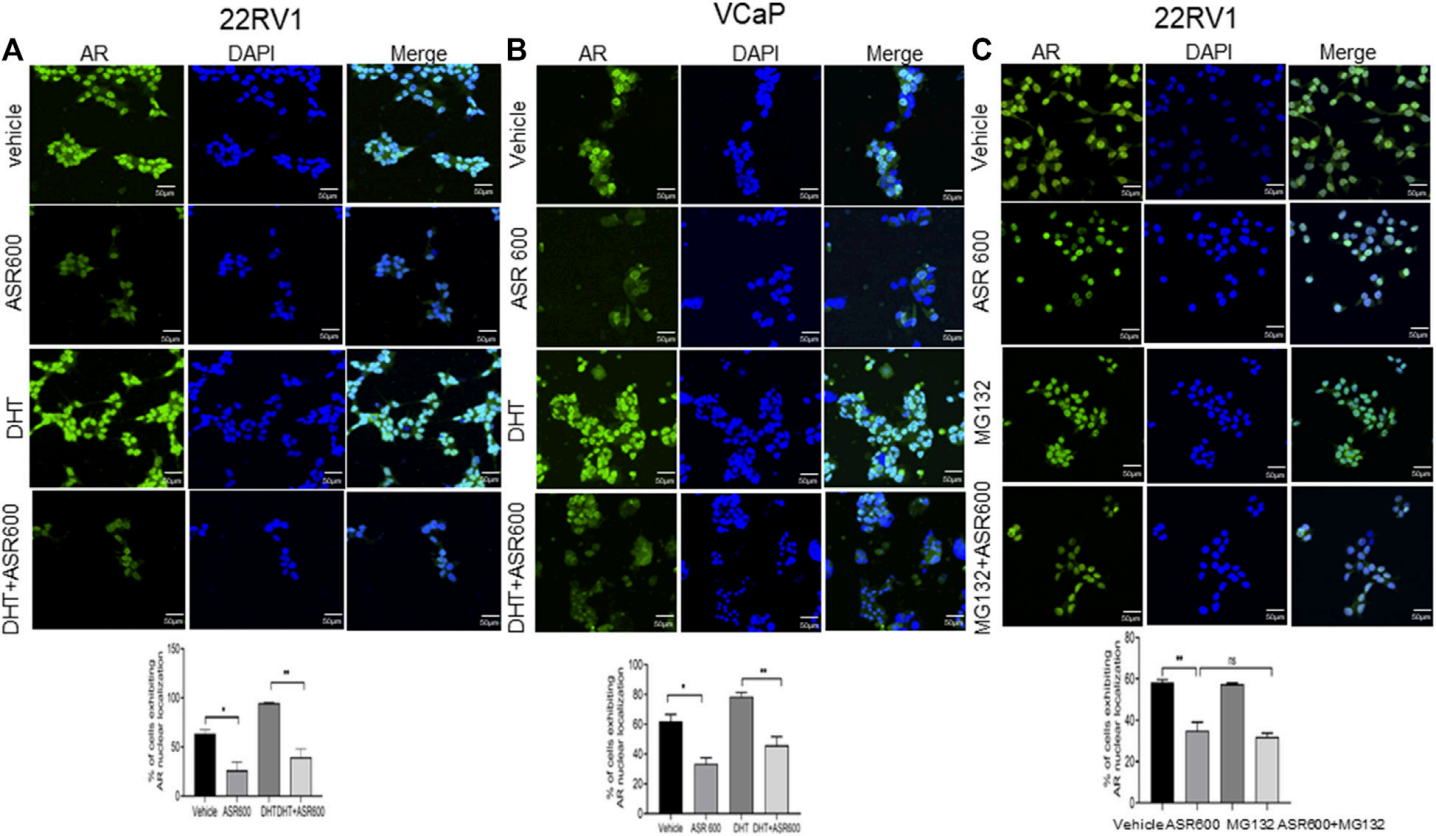
Nuclear accumulation of AR abolished by ASR-600. **(A, B)**. Immunofluorescence analysis of cyto-nuclear localization of AR expression in ASR-600, DHT, or ASR 600 + DHT treated 22RV1 and VCaP cells. **(C)**. Immunofluorescence analysis of cyto-nuclear localization of AR expression in ASR-600, MG132 (10 µM), or ASR 600 + MG132 treated 22RV1 cells.

Because the AR^+^ CaP cell lines were found to be sensitive to ASR-600 treatment, we next sought to determine whether ASR-600 mediated its effects *via* targeting AR signaling. A time-dependent downregulation of AR expression as well as a concomitant decrease of AR downstream target prostate-specific antigen (PSA), was seen in a panel of CaP (C4-2B, LNCaP, EnZ-C4-2B, VCaP and 22Rv1) cell lines after treatment with their respective IC_50_ concentrations of ASR-600 (Figures 1E-G, I, J). A dose-dependent decline in AR expression was also observed in C4-2B cells (Figure 1H). We also noted that ASR-600 decreased the expression of AR-V7 in 22Rv1 and VCaP cells, suggesting that ASR-600 targets not only the AR-FL but also AR-Vs, which is associated with aggressive CaP phenotypes. We next explored the effects of ASR-600 on DHT-induced AR signaling in C4-2B and 22Rv1 cells. Results indicated that ASR-600 treatment abolished DHT-induced AR signaling in both cell lines, as confirmed by the decreased expressions of AR and PSA (Figures 2A, B). Next, to understand whether the inhibitory activity of ASR-600 is specifically through AR signaling, we overexpressed AR in 293T and prostate-specific AR-null PC-3 cells. ASR-600 suppressed the expression of AR-FL in both cell lines (Figures 2C, D). Together these data confirm AR could be a target for ASR-600. Subsequent immunofluorescence analysis reconfirmed that ASR-600 treatment resulted in the overall loss of AR in DHT-treated 22Rv1 and VCaP cells (Figures 3A, B) and inhibiting proteasomal activity by MG132 (10 µM) inhibited loss of AR in ASR-600 treated 22Rv1 cells (Figure 3C).

### ASR-600 inhibits AR-V7 expression in CaP cells

**FIGURE 4:**
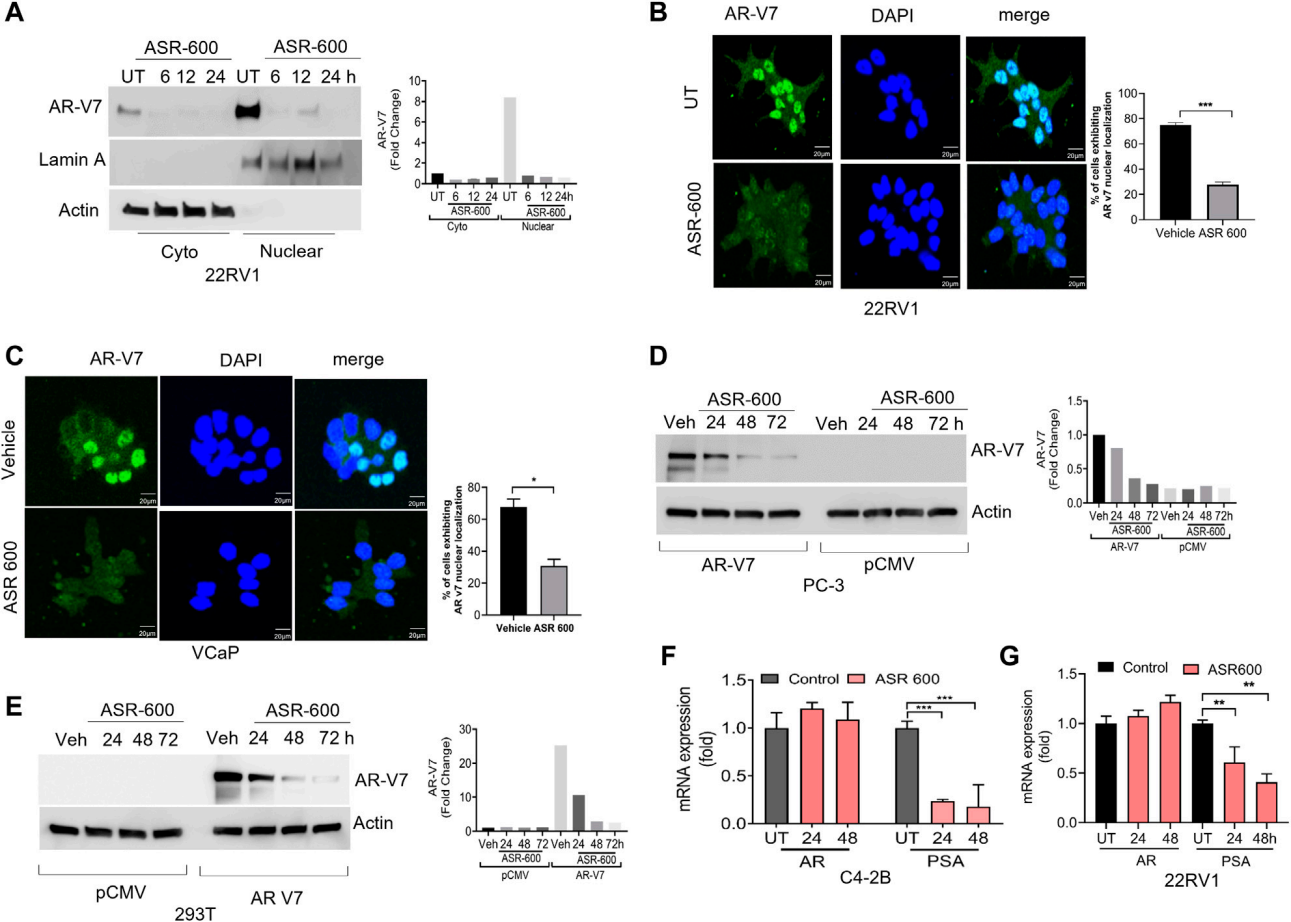
ASR-600 inhibited AR-V7 expression in CaP cells. **(A)** Immunoblots demonstrating subcellular fractionation in 22RV1 cells treated with ASR-600 for 6, 12, and 24 h. Lamin A and ß-actin were used as the internal controls. **(B, C)** Immunofluorescence analysis of cyto-nuclear localization of AR-V7 expression in ASR-600 treated 22Rv1 and VCaP cells. **(D, E)** Immunoblots for AR-V7 expression in PC-3 or 293T cells transfected with AR V7 or empty vector and treated with ASR-600 for 24, 48 and 72 h. **(F, G)** AR and PSA mRNA expression of ASR-600 treated C4-2B and 22Rv1 cells.

We examined the cytosolic and nuclear expression of AR-V7 in ASR-600 treated 22Rv1 cells. Results demonstrate that ASR-600 decreases both cytoplasmic and nuclear AR-V7 in a time‐dependent manner (Figure 4A). Subsequent, immunofluorescence analysis revealed a significant reduction in nuclear expression of AR-V7 in ASR-600 treated 22Rv1 and VCaP cells when compared to vehicle-treated cells (Figures 4B, C). As a proof of principle, we overexpressed AR-V7 in PC-3 and 293T cells and observed that ASR-600 completely abolished AR-V7 expression in both cell lines (Figures 4D, E). These results confirm that ASR-600 inhibits AR-V7 expression in CaP cell lines. Next, we analyzed the effect of ASR-600 on the transcriptional levels of AR and PSA in CaP cells. Surprisingly, no significant reduction of AR mRNA levels was observed. However, a significant reduction of PSA levels was seen in the CaP cells suggesting that AR inhibition by ASR-600 may be post-transcriptionally regulated (Figures 4F, G).

### Downregulation of AR by ASR-600 may involve ubiquitin signaling

**FIGURE 5:**
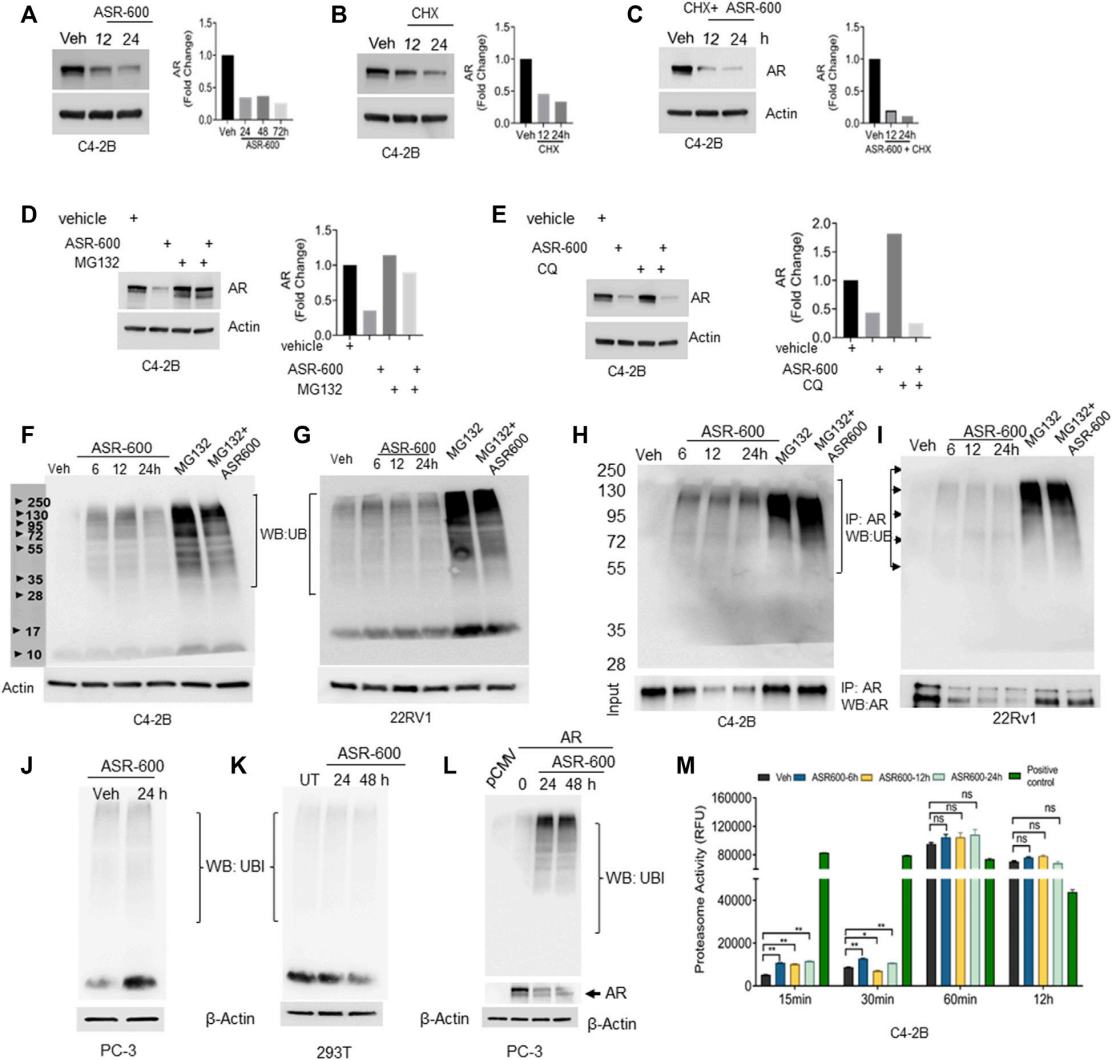
Ubiquitination of AR in ASR-600 treated CaP cells. **(A–C)** Immunoblots of AR expression in C4-2B cells treated with ASR-600, CHX (50 µM) or combinations at the indicated time points. **(D,E)** Immunoblots of AR expression in C4-2B cells treated with ASR-600, MG-132(10 µM) or CQ (50 µM), or combinations. **(F,G)** Immunoblots for ubiquitin protein expression in C4-2B and 22Rv1 cells were treated with MG-132, ASR-600, veh, or combinations for 6, 12, and 24 h **(H,I)**. Cell lysates were IP with AR antibody and subjected to WB with ubiquitin antibody. The input cell lysates were WB with AR. **(J,K)** Immunoblots of ubiquitin expression in PC-3 and 293T cells were treated with ASR-600 at the indicated time points. **(L)** PC-3 cells transfected with AR and treated with Veh or ASR600 for 0, 24, and 48 h. **(M)** The proteasomal activity was measured in C4-2B cells treated with ASR-600 at the indicated time points.

As ASR-600 seems to have an inconsistent inhibitory effect on AR mRNA, as compared to AR protein levels, we investigated ASR-600 post-translational regulation of AR. We found that treatment with protein synthesis inhibitor CHX alone and in combination with ASR-600 resulted in a time-dependent decrease of AR expression in C4-2B cells (Figures 5A–C), suggesting the possible involvement of AR degradation signaling in ASR-600 mediated effects. To further explore these results, we examined degradation pathways that may be involved in the ASR-600-regulated decrease of AR. As the ubiquitin-proteasome or lysosome pathways are the predominant mechanisms for AR degradation, C4-2B cells were treated with ASR-600 in the presence or absence of a proteasome (MG132) and lysosome (CQ) inhibitor for 9 h. While the decrease in AR levels upon ASR-600 treatment was significantly rescued when MG132 was used to inhibit proteasomes, the same was not observed when cells were treated with the lysosomal inhibitor (Figures 5D-E). These results suggest that AR degradation is caused by proteasome activation.

Next, we examined ubiquitination-associated AR degradation in ASR-600 treated CaP cells. Increased ubiquitin expressions were seen in ASR-600 treated C4-2B and 22Rv1 cells (Figures 5F, G). MG132 treatment which inhibits proteasome mediated degradation of proteins causes polyubiquitination. Hence, we have used MG-132 as a positive control in these experiments. To confirm that ASR-600 specifically ubiquitinates AR, we analyzed ubiquitin expression in ASR-600 treated AR-null PC-3 and 293T cells. No change in ubiquitin expression was observed (Figures 5J, K). These results further confirm that ASR-600 treatment specifically ubiquitinates AR. However, when we overexpressed AR in AR-null PC-3 cells and treated them with ASR-600, we observed an increased expression of ubiquitin compared to the vehicle which suggests that ASR-600 specifically targets AR in CRPC cells (Figure 5L). We also examined ubiquitination-associated AR degradation by immunoprecipitation (IP) with AR and immunoblot for ubiquitin antibody on CaP cell lysates treated with MG-132, ASR-600 and combinations. ASR-600 induced AR ubiquitination in both C4-2B and 22Rv1 cells.

Moreover, MG-132 increased AR ubiquitination which was further increased in both cell lines on treatment with a combination of MG-132 and ASR-600 (Figures 5H, I). To determine whether ASR-600 could be a proteasome inhibitor, we measured proteasomal activity using a chymotrypsin-like compound with a 7-amido-4-methylcoumarin (AMC)-tagged peptide substrate. An induction of proteasome activity was measured at 15 and 30 min and no significant changes were noted until 12 h in ASR-600-treated CaP cells (Figure 5M). We used commercially available positive and negative controls for these experiments. These results suggest that ASR-600 is not a proteasome inhibitor. Together, these results indicate that ASR-600 is a potent ubiquitination agent for AR in CaP cells.

### ASR-600 specifically targets AR signaling

**FIGURE 6:**
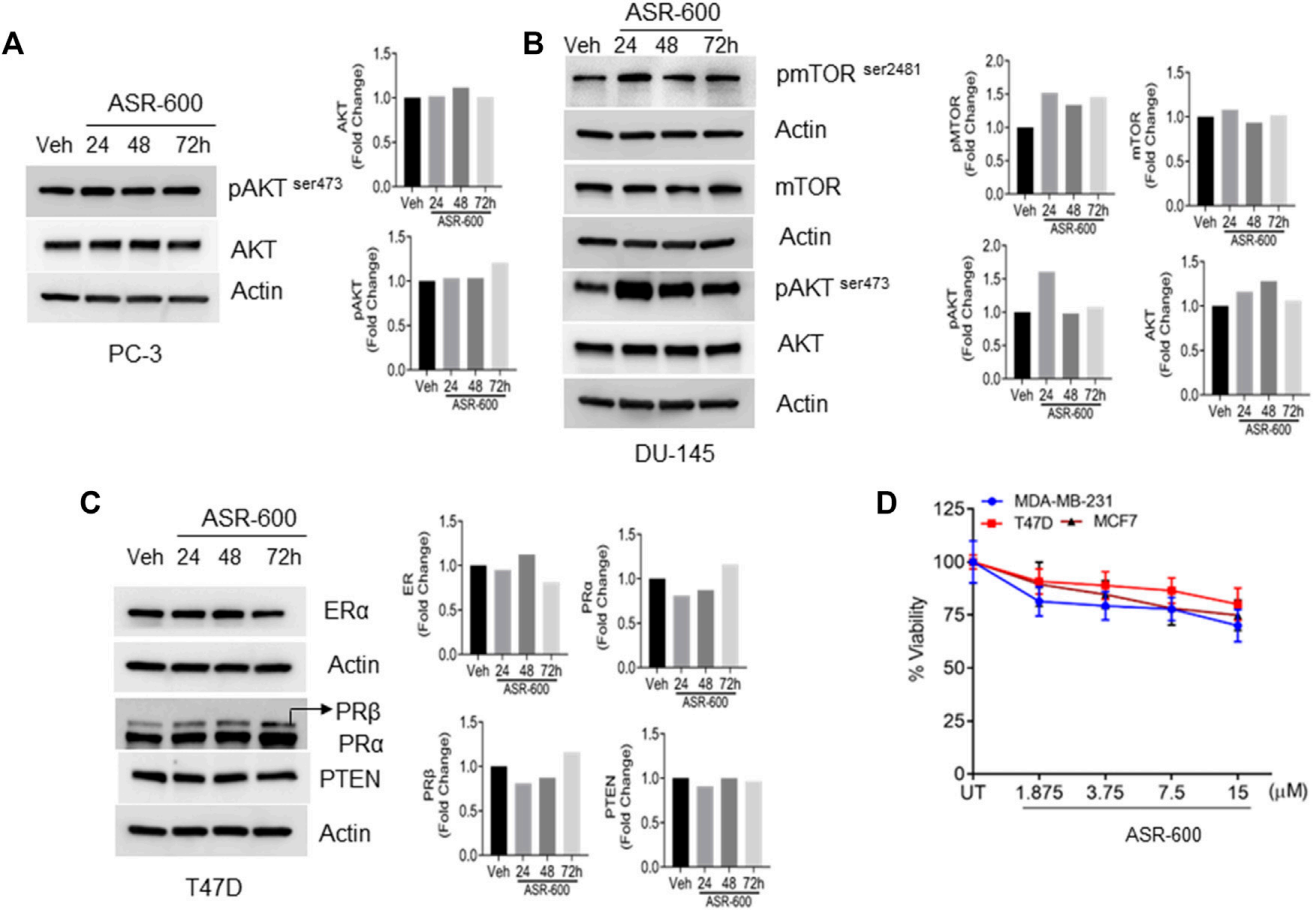
ASR-600 specifically targets AR in CaP cells: **(A–C)**. Immunoblots of indicated antibodies in vehicle or ASR-600 treated PC-3, DU-145, and T47D cell lines. **(D)**. Cell viability assay results of vehicle or ASR-600 treated ER-positive and ER-negative breast cancer (MCF-7, T47D, and MDA-231) cell lines.

To confirm whether ASR-600 affects other pro-survival signaling pathways apart from AR in CRPC, we examined AKT, mTOR, Estrogen receptor (ER), and Progesterone receptor (PR) expressions in prostate and breast cancer cell lines. No significant changes in the expressions pAKTser243 were observed in the ASR-600 treated PC-3 cells (Figure 6A). Similarly, we confirmed that ASR-600 did not alter the expression of pmTOR^Ser2481^, and pAKT^ser243^ in DU-145 cells (Figure 6B). Moreover, assessment of ASR-600 effect on other closely related receptors in T47D cells, revealed that ASR-600 did not affect ER and PR levels. Moreover, no changes in the PTEN levels were seen in the ASR-600 treated cells (Figure 6C). As expected, ASR-600 failed to inhibit the growth of breast cancer cells (Figure 6D), which supports the notion that ASR-600 specifically targets AR in CaP cells.

### ASR-600 binds to NTD of AR

**FIGURE 7:**
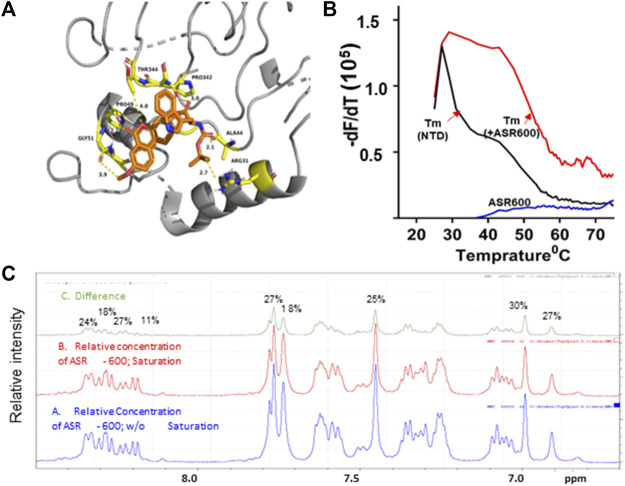
Physical interaction between ASR 600 and AR- NTD: **(A)**. AR-NTD residues participating in potential electrostatic and steric interactions with ASR-600 are depicted in yellow stick representation. The AR-NTD backbone is shown as a cartoon in gray, and ASR-600 is represented as an orange stick representation. **(B)**. GloMelt™ thermal shift assay was performed on NTD (5 µg) in the presence of ASR-600. The binding stabilized the protein, as indicated by the shift in the melting curves. **(C)**. NMR STD experiment results of ASR-600 with AR-NTD; the STD effect varied from 18 to 30%. The figure shows the aromatic region as proton peaks of ASR-600: the blue spectrum-without saturation; the red - with saturation; and the green depicts the difference in the spectrum observed.

We performed *ab initio* modeling to decipher the 3D structure of AR-NTD and used molecular docking studies to assess the binding affinity of ASR-600 to the NTD domain of AR. Visual inspection of the NTD-ASR-600 docked structures revealed compact binding between the ligand and NTD (Figure 7A). The interaction energy of ASR-600 with NTD displayed a moderate (−29.71 kcal/mol) binding energy. Next, we confirmed that ASR-600 binds to NTD by using a commercially available kit which works on a different principle (differential scanning fluorimetry). The first derivate of the fluorescence curve (-dF/dT) was plotted against temperature to calculate Tm (the temperature at which 50% of the double-stranded DNA dissociates into single strands) (lowest–dF/dT value). The purified NTD protein alone had a melt temperature of approx. 35.0°C and ASR-600 treatment shifted the melt temperature (ΔTm) by 12°C–15°C (median of 13.5°C) in a distribution that appeared normal (Figure 7B).

Finally, we utilized NMR spectroscopic studies to confirm the binding of ASR-600 to AR-NTD. The principle of this experiment is that in the bound state, the most abundant molecule governs the hydrodynamic properties of both ligand and receptor. Thus, the magnetization transfer in the on-resonance experiment leads to a significant reduction in signal intensities for the ligand, which is recorded in the spectra. The off-resonance irradiation experiment will not affect the spectra intensities of the ligand because there is no transfer of magnetization from protein to ligand, which serves as the reference spectra. Subtraction of the on-resonance spectra from off-resonance spectra indicates the binding interaction between ligand and protein (Figure 7C). In contrast, subtraction of the no-binding ligands will result in a flat line spectrum because no magnetization transfer takes place during both on- and off-resonance irradiation (This distinction provides the basis for NMR screening experiments. The STD effects observed were 30–18%, indicating a significant binding to NTD (Figure 7C).

### *In vitro* metabolic stability

**FIGURE 8:**
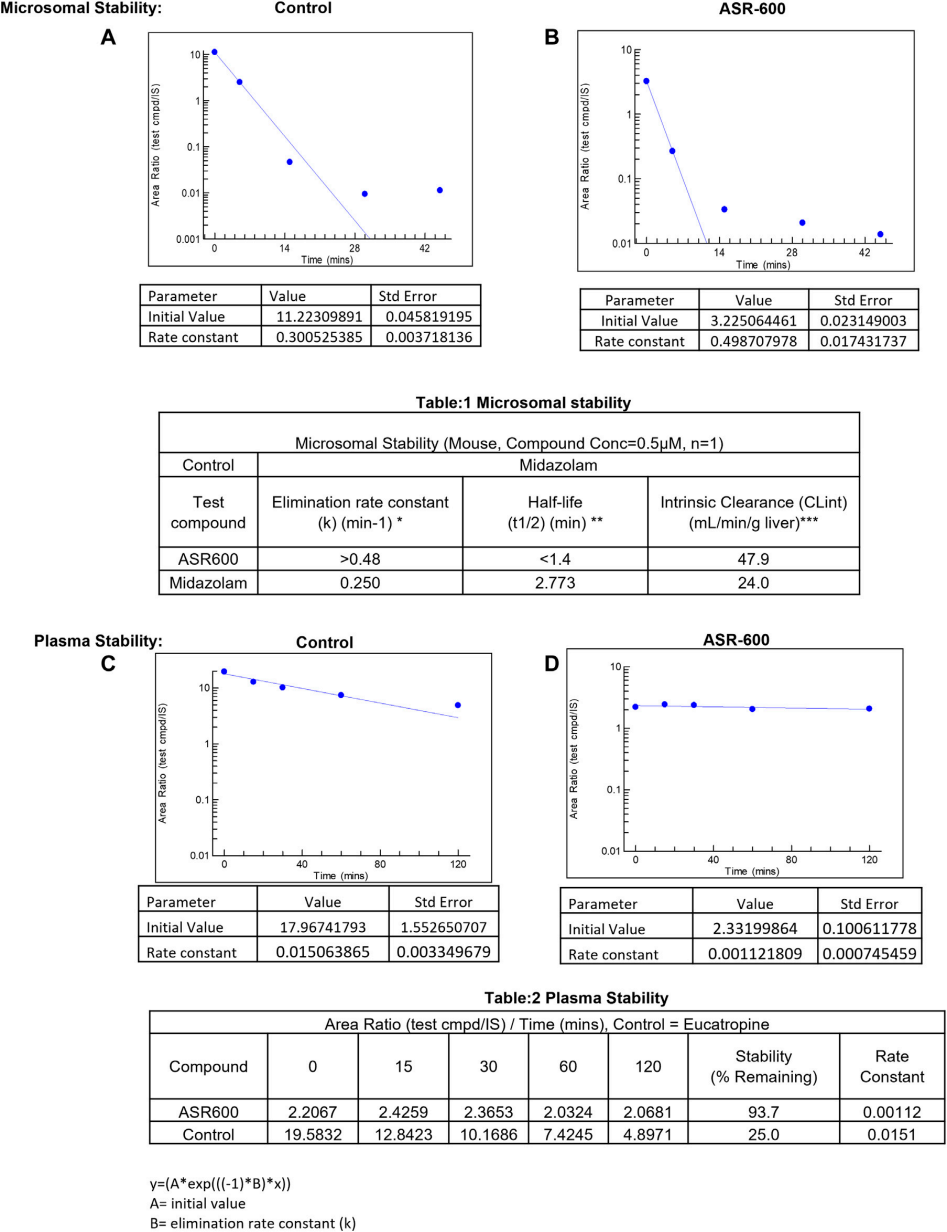
Microsomal and plasma stability ASR-600. Mouse microsomes were thawed and mixed with ASR-600 or control (Midazolam) **(A)**. ASR-600 and microsomes were incubated with or without cofactor (+NADPH) for indicated time points and plotted against area ratio. **(B)**. The area ratio was plotted against indicated time points for Midazolam **(Table:1)** Mouse microsomal stability. The elimination rate constant, half-life and intrinsic clearance of ASR-600 and control: Midazolam. Mouse plasma was mixed with ASR-600 or control (Eucatropine) **(C)**. ASR-600 and mouse plasma were incubated for indicated time points and analyzed with LC-MS/MS. **(D)**. The area ratio was plotted against indicated time points for Midazolam **(Table:2)** Plasma stability. Stability and rate constant of ASR-600 and control: Eucatropine.

*In vitro* systems, such as human liver microsomes (HLM) and human hepatocytes, are the best models to predict a drug’s hepatic clearance (Gajula et al., 2021). At a concentration of 0.5 µM, the elimination rate constant k) of ASR-600 in mice liver microsome metabolism was >0.48 min^−1^ (control: Midazolam, k = 0.250 min^−1^) with a half-life (t½) of <1.4 min (Midazolam, t½ = 2.773 min) (Figures 8A, B, Table 1).

ASR-600 intrinsic clearance (CLint) was also calculated based on the *in vitro* t½ (Baranczewski et al., 2006) so the CLint of ASR-600 was 47.9 mL/min/g (Midazolam, 24.0 mL/min/g). ASR-600 was highly stable in the mouse plasma *in vitro*, 93.7% remaining after 2 h incubation time as compared to 25% in control i.e., Eucatropine (Figure 8D, E, Table 2). The high stability of ASR-600 in the mouse plasma suggests that ASR-600 is not subject to cleavage of any significant levels by the enzymes resided in the systemic circulation after it is intravenously administered to the animals.

### ASR-600 inhibits the growth of CRPC tumors in castrated and non-castrated xenograft mouse models

**FIGURE 9:**
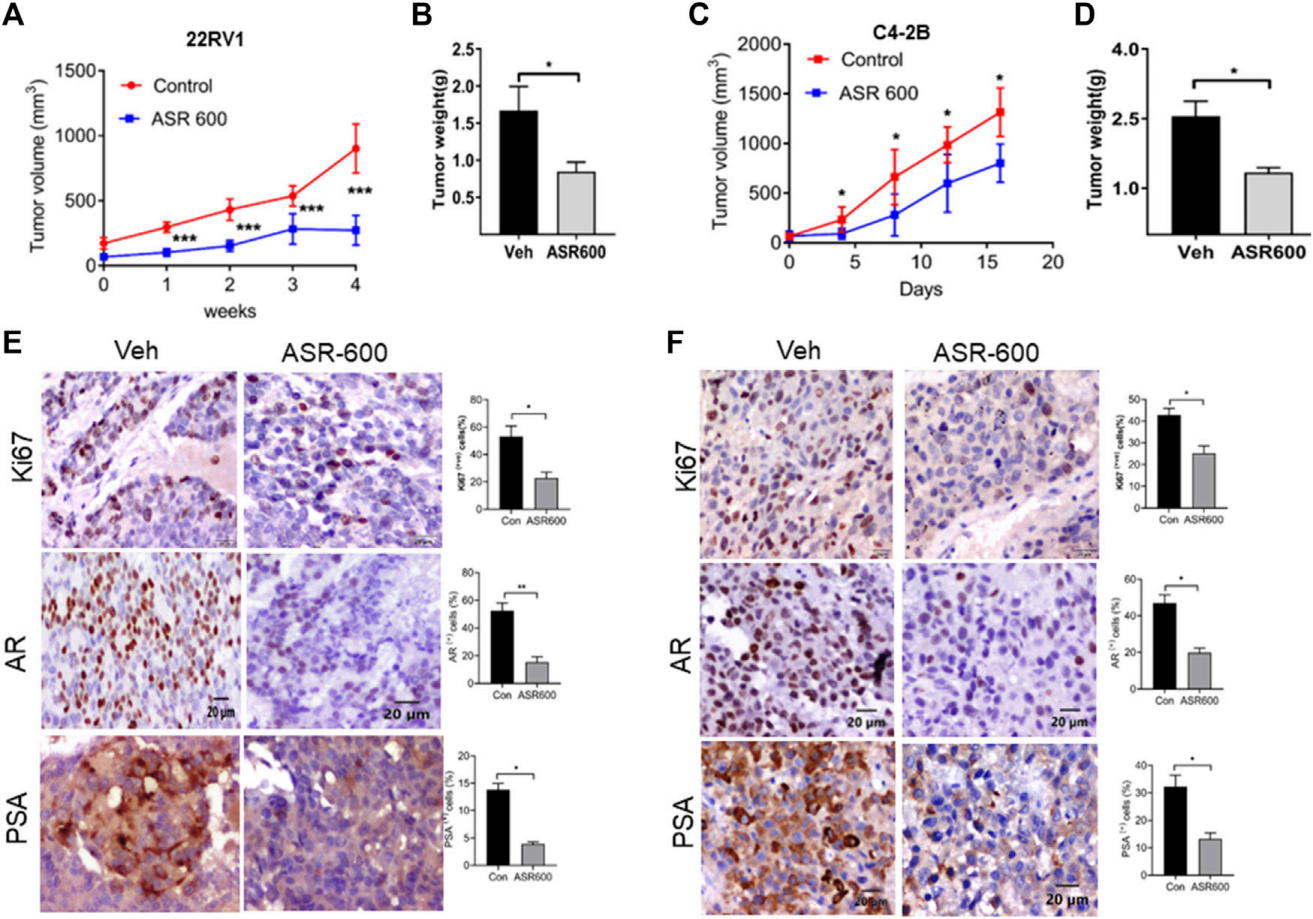
The therapeutic effect of ASR-600 on Xenotransplanted prostate tumors. **(A, C)** Oral administration of ASR-600 (20 mg/kg) inhibited the xenotransplanted tumors from 22Rv1 & C4-2B tumors. Tumor volumes were measured once per week for 4 weeks, and a line graph was plotted to compare tumor growth volume (mm^3^). **(B, D)** Tumor weight for vehicle and ASR 600-treated 22Rv1 and C4-2B tumors. **(E, F)** Immunohistochemistry analyses of tumor samples were performed to evaluate the expression of Ki67, AR and PSA in 22Rv1 and C4-2B xenografted tumors.

The anti-cancer effect of ASR-600 was also evaluated *in vivo* using castrated and non-castrated CRPC xenograft mouse models. ASR-600 treatment was highly effective in reducing tumor volumes of both 22Rv1 and C4-2B xenograft models (Figures 9A, C). The weights of tumors from ASR-600 treated mice were also lower than that of the vehicle-treated group (Figures 9B, D). IHC analysis revealed that ASR-600 decreased AR and PSA expression in treated tissues. This confirms that ASR-600 effectively decreases AR levels and downregulates AR signaling. ASR-600 treated tumors also showed low Ki67 + nuclei, which confirms the growth inhibitor effect of ASR-600 in CRPC xenograft models (Figures 9E, F). Examination of the body weight of the treated mice revealed that ASR-600 had no toxic pathological effect on their growth during the treatment period (data not shown). These results suggest that the 20 mg/kg dose of ASR-600 used did not induce any significant toxicity in the mice.

**FIGURE 10:**
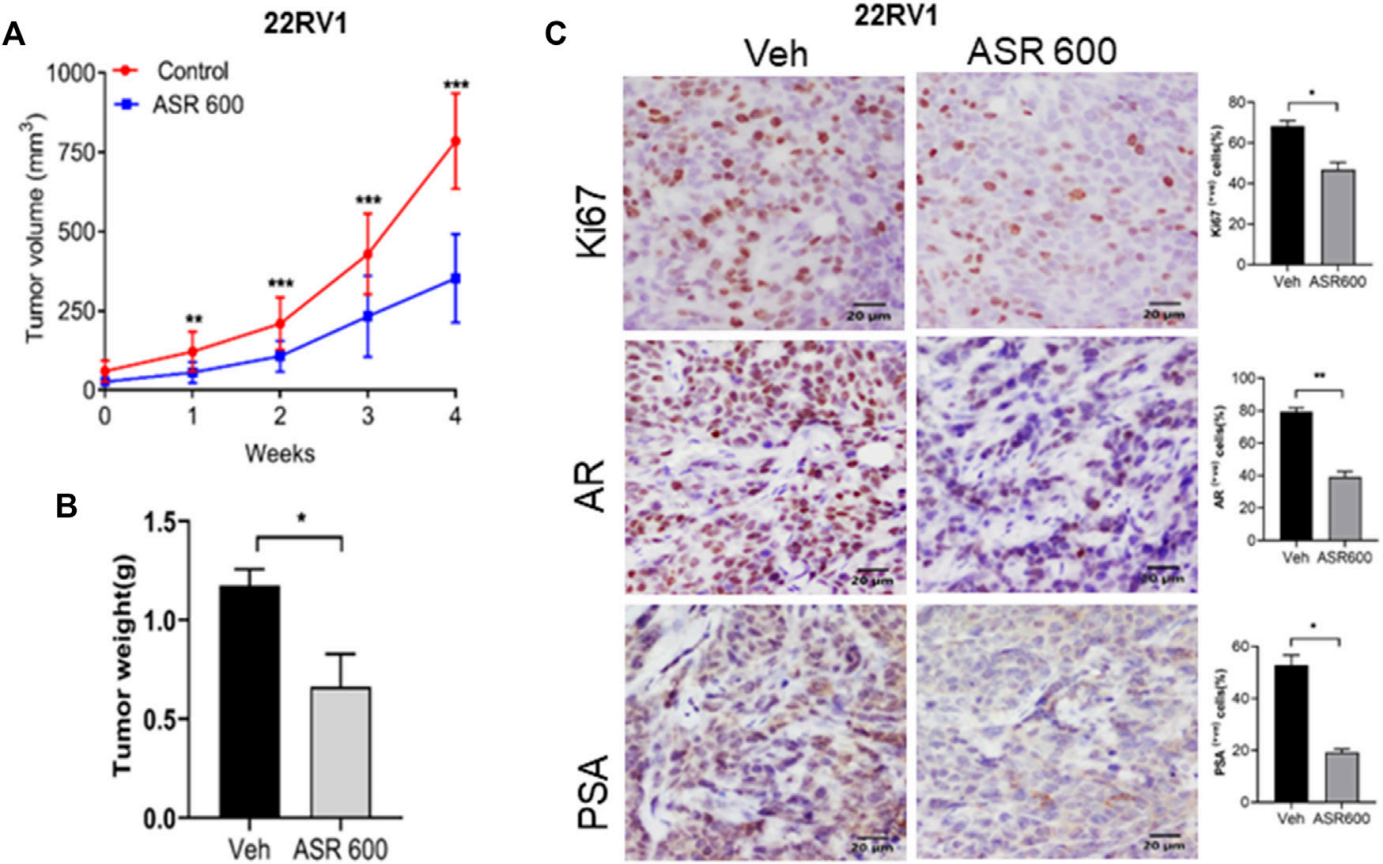
Oral administration of ASR-600 inhibits the growth of 22Rv1 xenograft tumors in castrated mice. 22Rv1 cells were inoculated into castrated nude mice. When the tumors reached ∼50 mm^3^, the mice were treated with 20 mg/kg ASR-600 through oral gavage. **(A)**. Mean tumor volumes. **(B)**, Individual tumor weight was measured. **(C)**. Immunohistochemistry analyses of tumor samples were performed to evaluate the expression of Ki67, AR and PSA in 22Rv1 xenografted tumors.

Next, we evaluated the ability of ASR-600 to inhibit the growth of 22RV1 tumors in castrated mice. 22RV1 cells were injected into the right dorsal flank of castrated male nude mice. ASR-600 (20 mg/kg) was administered by oral gavage throughout the experiment period. ASR-600 significantly inhibited the growth of 22RV1 tumors and decreased the tumor weights of treated mice (Figures 10A, B). IHC analysis of the xenograft tumors revealed that ASR-600 decreased AR and PSA expression in treated tissues (Figure 10C). This confirms that ASR-600 effectively decreases AR levels and downregulates AR signaling. ASR-600 treated tumors also showed low Ki67 + nuclei, which confirms the growth inhibitory effect of ASR-600 in CRPC castrated xenograft models. Our results indicate that ASR-600 may be a useful treatment for CRPC.

## Discussion

We show here that ASR-600, an analog of UroA, is more potent than its parent compound and that it specifically targets and causes AR-FL and AR-V7 degradation in CRPC and enzalutamide resistant CaP cells *via* ubiquitin-mediated pathway. NMR and ITC studies revealed that ASR-600 binds to the AR-NTD domain and enhances ubiquitin-mediated degradation of both AR-FL and AR-V7 without inhibiting proteasome activity.

Upregulation or reactivation of AR signaling is a hallmark of CaP progression to CRPC (Chen et al., 2004; Schmidt and Tindall, 2013; Tan et al., 2015; Cato et al., 2019), which resulted in the development of anti-AR drugs (abiraterone and enzalutamide) targeting the AR axis (de Bono et al., 2011; Beer et al., 2014). Abiraterone reduces androgen production by blocking cytochrome P450 17 alpha-hydroxylase (CYP17) (Attard et al., 2009); enzalutamide has a three-fold effect it competitively inhibits androgen binding to AR, prevents AR translocation to the nucleus, and inhibits AR binding to androgen response elements in the nucleus (Tran et al., 2009; Watson et al., 2010). Although these modalities block AR signaling, they do not target AR (e.g., degradation of AR), which may continue to function through other stimuli (e.g., growth factors), resulting in disease relapse and progression. Moreover, the emergence of constitutively active AR-Vs that lack the LBD has contributed to the development of resistance in CRPC patients (Guo et al., 2009; Hu et al., 2009; Li et al., 2013; Scher et al., 2016; Welti et al., 2016). Hence, targeting both AR-FL and AR-Vs may not only disrupt these interactions but also curb the growth of CRPC.

Recently, a phase-1 clinical trial on the AR-NTD inhibitor (EP1-506) was terminated because it achieved a minor decline in serum PSA levels (a surrogate marker of disease progression) (4–29%), and only 3 out of 21 patients with metastatic CRPC (Ronan Le Moigne et al., 2019). Another agent (ARV-110), a PROTAC^®^ drug that uses an E3 ligase to tag and degrades “clinically relevant mutated AR proteins”, has been introduced in a limited Phase-I dose escalation for patients with metastatic CRPC. Hence, as of now, enzalutamide, abiraterone, and apalutamide are the US FDA-approved agents for treating CRPC. Considering that CRPC is the leading cause of 30,000 CaP-related mortalities every year in the US (American Cancer Society, 2019), there is an unmet clinical need for developing orally bioavailable and minimally toxic drug-like small molecules to effectively treat CRPC.

Previously, we reported that the anti-cancer properties of UroA occurred when the compound was administered in micromolar (IC_50:_35 µM) concentrations (Dahiya et al., 2018). However, in the present study, we found that novel UroA analog ASR-600 inhibited the growth of AR^+^ CRPC, including enzalutamide resistant cells, at nanomolar concentrations, a dose that is 40-times lower than that of UroA (Boakye et al., 2018). Moreover, ASR-600 was found to be non-toxic to normal prostate epithelial cells. Our results also revealed that ASR-600 specifically targets AR. It is well known that UroA inhibits AKT and mTOR while upregulating PTEN in many cancer types (Zhou et al., 2016; Liberal et al., 2017; Boakye et al., 2018). These target genes are also expressed by AR null PC-3 and DU-145 CaP cells, and ASR-600 failed to inhibit pAKT and mTOR in AR-null CaP cells. This compound did not affect on PTEN levels as well as the ER and PR receptors in T47D cells. Similarly, although both DU-145 and PC-3 cells express the glucocorticoid receptor (Guo et al., 2018), ASR-600 still failed to inhibit the growth of both cell lines. Recently, Sharp et al. (2019) reported that while AR-V7 is rarely expressed in primary CaP, it is predominantly expressed in CRPC and frequently detected in CaP cases following ADT treatment and further increased following treatment with abiraterone or enzalutamide. We observed that ASR-600 decreased the expression of AR-V7 in 22Rv1 and VCaP cells and ectopically overexpressed AR-V7 293T cells. These results suggest that ASR-600 can target both AR-FL and AR-V7.

The ubiquitin-proteasome pathway is the predominant mechanism for AR degradation. Liu et al. (2018) discovered that the ubiquitin-mediated proteolysis pathway and proteasome activity are suppressed in enzalutamide and abiraterone-resistant CaP cells, which may trigger the over-expression of oncoproteins such as the AR-Vs. They noted that the half-life of AR-V7 is significantly extended in enzalutamide-resistant CaP cells suggesting that the treatment may alter the CaP ubiquitin-proteolysis system and stabilize the AR-V7 protein. Similarly, our results show that co-treatment of ASR-600 and CHX, a protein synthesis inhibitor, induced a ∼90% decrease in AR protein levels, suggesting that ASR-600 mediated AR degradation may occur either by ubiquitin or lysosome pathways (Dikic, 2017). It is interesting to consider that AR downregulation in C4-2B cells was reversed by treatment with MG-132, a proteasome inhibitor (Steinhilb et al., 2001), but not with CQ, a lysosomal inhibitor. Thus, our mechanistic studies revealed that ASR-600 causes AR and AR-Vs degradation in CRPC and enzalutamide-resistant CRPC cells through a ubiquitin-mediated pathway. Biophysical analysis based on NMR and ITC studies indicated that ASR-600 binds to NTD and enhances ubiquitin-mediated degradation of both AR and AR-Vs without inhibiting proteasome activity. Our ongoing experiments may suggest the possible ASR-490 ubiquitin binding sites at NTD of AR. We believe that if we ubiquitinate AR, regardless of whether or how much androgen or other stimuli of AR signaling are present in CRPC, it would be a novel approach to eradicate the deadly disease.

Results assessing ASR-600 *in vivo* efficacy using castrated and non-castrated xenograft mice models showed that ASR-600 exhibited a high therapeutic index in that it was able to significantly inhibit tumor growth at just 20 mg/kg [<4% of the maximum tolerated dose (MTD)]. Considering that the MTD of the oral dose of ASR-600 in mice was >500 mg/kg of body weight, it is safe to assume that oral dose can be increased without any concerns of ASR-600 being systemically toxic.

In conclusion, despite progress in this field, many second-generation anti-androgens have limited clinical success owing to their inability to block AR-V7 signaling in CRPC. Thus, to develop more efficient and target-specific treatments, a greater understanding of the regulatory mechanisms of AR-V7, its upstream and downstream effectors, and target genes is essential. Our findings highlight the importance of targeting AR-Vs, particularly AR-V7 in CRPC, and provides mechanistic insight into how UroA analog ASR-600 can target both AR-FL and AR-V7 in CRPC.

## Data Availability

The original contributions presented in the study are included in the article/Supplementary Materials, further inquiries can be directed to the corresponding author.
