# Supplementary material for: Urolithin A analog inhibits castration-resistant prostate cancer by targeting the androgen receptor and its variant, androgen receptor-variant 7
Source: Front Pharmacol. 2023 Mar 3;14:1137783. doi: 10.3389/fphar.2023.1137783 (PMC10020188; doi:10.3389/fphar.2023.1137783)
Supplement: Supplementary file 1 [file DataSheet2.PDF]

In Vitro Metabolic Stability in Microsomes/S9

Scope of Service

The microsomal/S9 stability assay screens the in vitro metabolic stability of test compounds in microsomes or S9.

Assay Summary

Final Test Compound Concentration	0.5 µM
Final Protein Concentration	0.5 mg/mL(microsomes)<br>1 mg/mL (S9)
Time Points	0, 5, 15, 30, 45, 45 NC*
Positive Control	Midazolam<br>Umbelliferone (Phase II)
Replicates	n=1

*NC = No Cofactor

Solutions

1. Compound Working Solutions
   Dilute compound stock solutions to 0.05 mM in DMSO: Water (1:1, *v/v*)

2. Potassium Phosphate Buffer (KPi)
   KPi prepared at 100 mM , pH 7.4

3. NADPH Regenerating System, Cofactor (5X)
   Prepare a solution containing NADP (1.7 mg/mL, 2.22 mM) and G6P (7.8 mg/mL, 27.6 mM) in 100 mM KPi. Aliquot and store at -20º C. Warm to 37º C and add G6PDH (2.0 U/mL) before use.

   Optional cofactors (5X): UDPGA (2.6 mg/mL), PAPS (0.32 mg/mL), alamethicin (125 µg/mL)

4. Stop Solution
   ACN containing 100 ng/mL tolbutamide

General Procedures

1. Thaw microsomes/S9 in the water bath (37° C), and then keep on ice once thawed.

2. Add 200 µL stop solution/well to a 96-deep well sample collection plate. Cover plate with a sealing mat and keep on ice.

3. Prepare 0.625 mg/mL microsomal/S9 mixtures in KPi.

4. Set up a base plate with 1.0 mL glass vials and add 7.5 µL compound working solution/vial. Add 592.5 µL microsomal/S9 mixture/vial and mix thoroughly by pipetting.

5. Transfer 250 µL of the spiked microsomal/S9 mixtures into two parallel sets of 1.0 mL glass vials. One set will serve as the cofactor incubation (+NADPH) and the other set as the no-cofactor incubation (-NADPH).

6. Add 62.5 µL KPi/vial to the no-cofactor incubation vials (-NADPH), mix by pipetting and transfer 50 µL into the T=0 wells of the sample collection plate.

7. Add 62.5 µL cofactor solution/vial to the cofactor incubation vials (+NADPH), mix by pipetting and place into the incubator (37°C, 5% CO_2) with shaking at 200 rpm.

8. For the cofactor incubation (+NADPH), mix by pipetting and transfer 50 µL at 5, 15, 30, and 45 minutes into the corresponding wells of the sample collection plate.

9. For the no-cofactor incubation (-NADPH), mix by pipetting and transfer 50 µL at 45 minutes into the corresponding wells of the sample collection plate.

10. Vortex sample collection plate at 1700 rpm for 3 minutes.

11. Centrifuge sample collection plate at 1380 x g for 10 minutes.

12. Take a new 96-deep well plate and add 100 µL ultrapure water/well. Transfer 100 µL supernatant/well from the sample collection plate into the corresponding wells of the final plate.

13. Vortex the final plate at 1700 rpm for 1 minute and analyse samples by LC-MS/MS.

Data Analysis

The elimination rate constant (k, min^{-1}) is calculated using nonlinear regression fitting with the following equation:

$$C_t = C_{t=0} \times e^{(-k \times t)}$$

where,

$C_{t=0}$ = initial concentration represented as the peak area ratio (test compound peak area/ internal standard peak area)

C_t = concentration at t represented as the peak area ratio (test compound peak area / internal standard peak area)

e = base of the natural logarithm

t = time (min)

k = elimination rate constant (min^{-1})

The half-life ($t_{1/2}$, min) is calculated using the following equation:

$$t_{1/2} = \frac{0.693}{k}$$

where,

k = elimination rate constant (min^{-1})

The in vitro intrinsic clearance is calculated using the following equation:

$$CL_{int} = \frac{k}{n} \times \text{scaling factor}$$

where,

k = elimination rate constant (min^{-1})
n = protein concentration (mg/mL)

The scaling factors for mL/min/g liver are as follows:

48 mg protein/g liver for mouse,
46 mg protein/g liver for rat
36.7 mg protein/g liver for dog
39.7 mg protein/g liver for human
52.5 mg protein/g liver for monkey
52.5 mg protein/g liver for minipig
52.5 mg protein/g liver for rabbit

The scaling factors for mL/min/kg body weight are as follows:

2448 mg liver microsomal protein/kg body wt for mouse
1656 mg liver microsomal protein/kg body wt for rat
1192.7 mg liver microsomal protein/kg body wt for dog
972.6 mg liver microsomal protein/kg body wt for human
1575 mg liver microsomal protein/kg body wt for monkey
876.7 mg liver microsomal protein/kg body wt for minipig
1617 mg liver microsomal protein/kg body wt for rabbit

The percent remaining is calculated using the following equation:

$$\%\ Remaining = C_{t=0} \div C_{t=45} \times 100$$

where,

$C_{t=0}$ = initial concentration represented as the peak area ratio (test compound peak area/ internal standard peak area)
$C_{t=45}$ = concentration at t=24 minutes represented as the peak area ratio (test compound peak area/internal standard peak area)

Data Report

A spreadsheet summary report (Microsoft Excel file) including raw data, methodology, elimination rate constant, half-life, intrinsic clearance, and percent remaining is provided.

In Vitro Stability in Plasma or Blood

Scope of Service

The stability assay screens the in vitro stability of test compound in plasma or blood.

Assay Summary

Final Test Compound Concentration	1 µM
Time Points	0, 15, 30, 60, 120
Positive Control	Eucatropine: M, MK, H<br>Enalapril: R<br>Aclidinium: MP, D
Anticoagulant	K2-EDTA

Solutions

1. Compound Working Solutions
   Dilute test compound stock solutions to 0.1 mM with DMSO: Water (1:1, *v*/*v*)

2. Ultrapure Water

3. Stop Solution
   ACN containing 100 ng/mL tolbutamide

General Procedures

1. Thaw and pre-warm plasma or blood in the water bath (37°C).

2. Add 400 µL stop solution/well for plasma or 50 µL ultrapure water/well for blood to a 96-deep well sample collection plate. Cover plate with a sealing mat and keep on ice.

3. Set up a base plate with 1.0 mL glass vials and add 495 µL blood/vial.

4. Add 5 µL compound working solution/vial to the plasma or blood, mix by pipetting and transfer 50 µL into the T=0 wells of the sample collection plate. Allow blood to sit in the water for 1 minute and then add 200 µL stop solution/well.

5. Place the base plate into the incubator (37°C, 5% CO_2) with shaking at 200 rpm.

6. For remaining time points, mix plasma or blood by pipetting and transfer 50 µL at 15, 30, 60 and 120 minutes into the corresponding wells of the sample collection plate. For each time point, allow blood to sit in the water for 1 minute and then add 400 µL stop solution/well.

7. Vortex sample collection plate at 1700 rpm for 3 minutes.

8. Centrifuge sample collection plate at 1380 x g for 10 minutes.

9. Take a new 96-deep well plate and add 100 µL ultrapure water/well. Transfer 100 µL supernatant/well from the sample collection plate into the corresponding wells of the final plate.

10. Vortex the final plate at 1700 rpm for 1 minute.

11. For blood samples, centrifuge the final plate at 1380 x g for 10 minutes

12. Analyse samples by LC-MS/MS.

Data Analysis

The elimination rate constant (k, min^{-1}) is calculated using nonlinear regression fitting with the following equation:

$$C_t = C_{t=0} \times e^{(-k \times t)}$$

where,

$C_{t=0}$ = initial concentration represented as the peak area ratio (test compound peak area/ internal standard peak area)

C_t = concentration at t represented as the area ratio (test compound peak area / internal standard peak area)

e = base of the natural logarithm

t = time (min)

k = elimination rate constant (min^{-1})

The half-life ($t_{1/2}$, min) is calculated using the following equation:

$$t_{1/2} = \frac{0.693}{k}$$

where,

k = elimination rate constant (min^{-1})

Data Report

A spreadsheet summary report (Microsoft Excel file) including raw data, methodology, elimination rate constant, half-life, and percent remaining is provided.
