# Supplementary material for: Urolithin A analog inhibits castration-resistant prostate cancer by targeting the androgen receptor and its variant, androgen receptor-variant 7
Source: Front Pharmacol. 2023 Mar 3;14:1137783. doi: 10.3389/fphar.2023.1137783 (PMC10020188; doi:10.3389/fphar.2023.1137783)

Full Blots

Fig.1e
LNCaP
AR

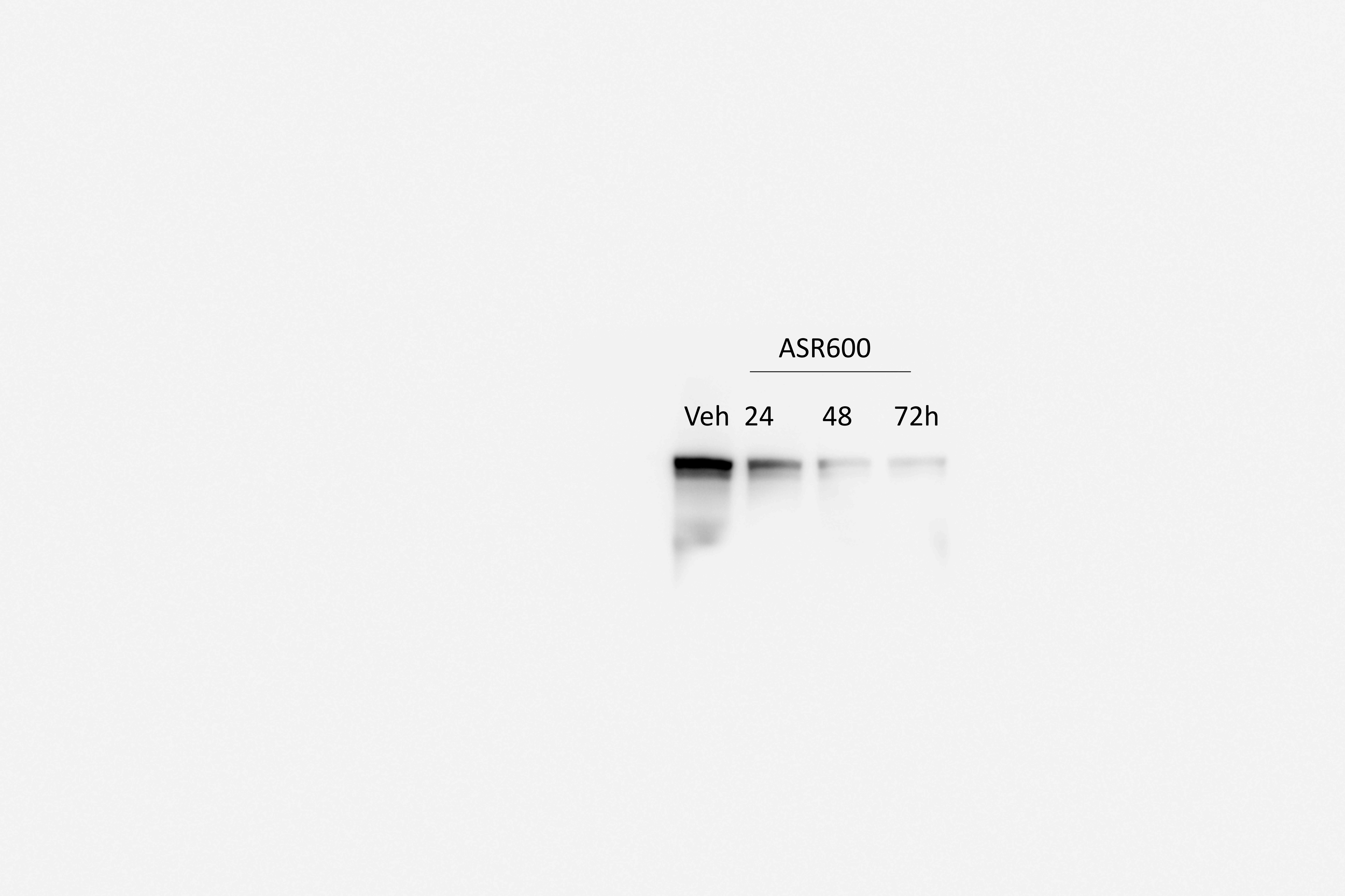

Fig.1e
LNCaP
PSA

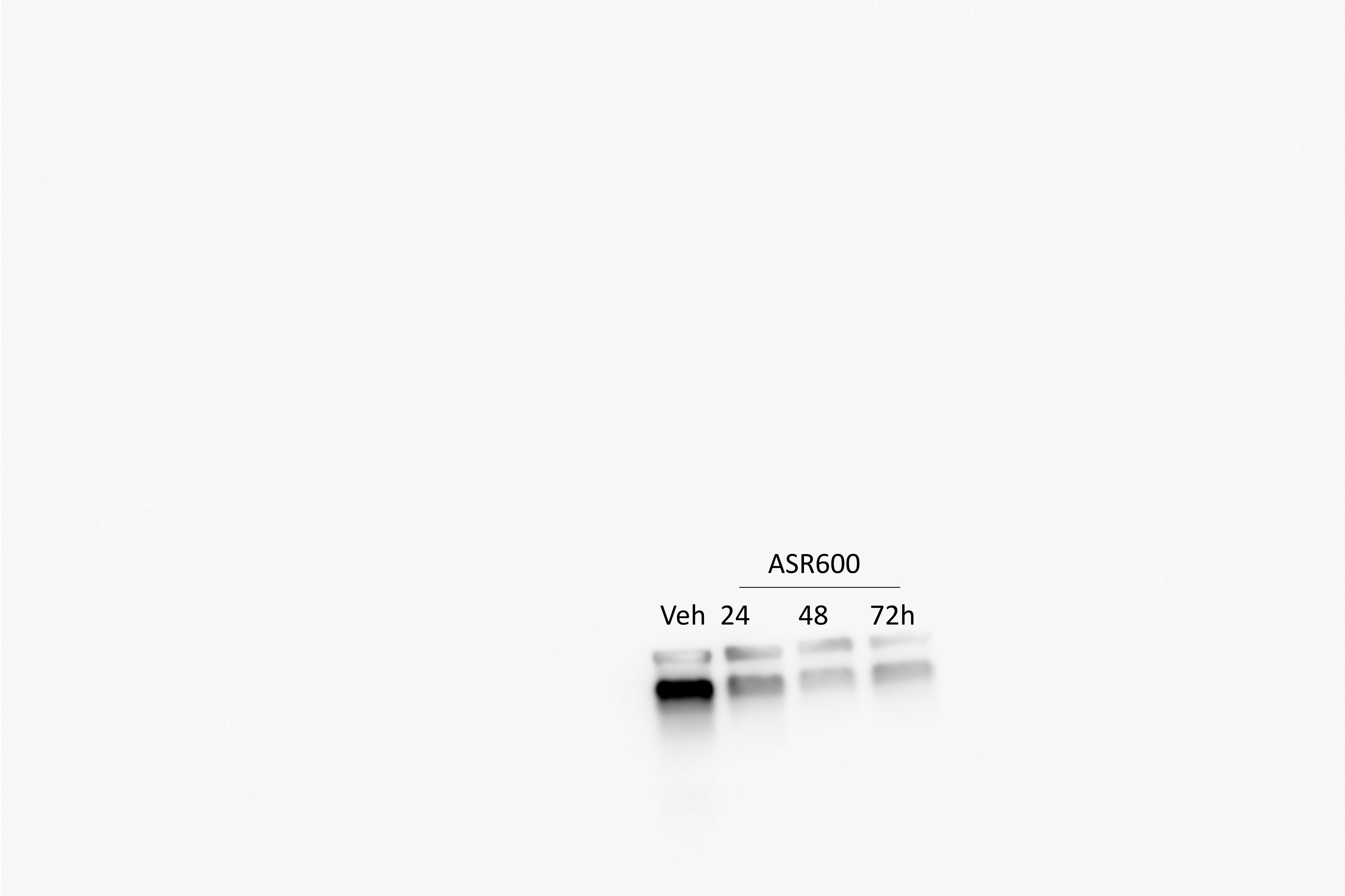

Fig.1e
LNCaP
Actin

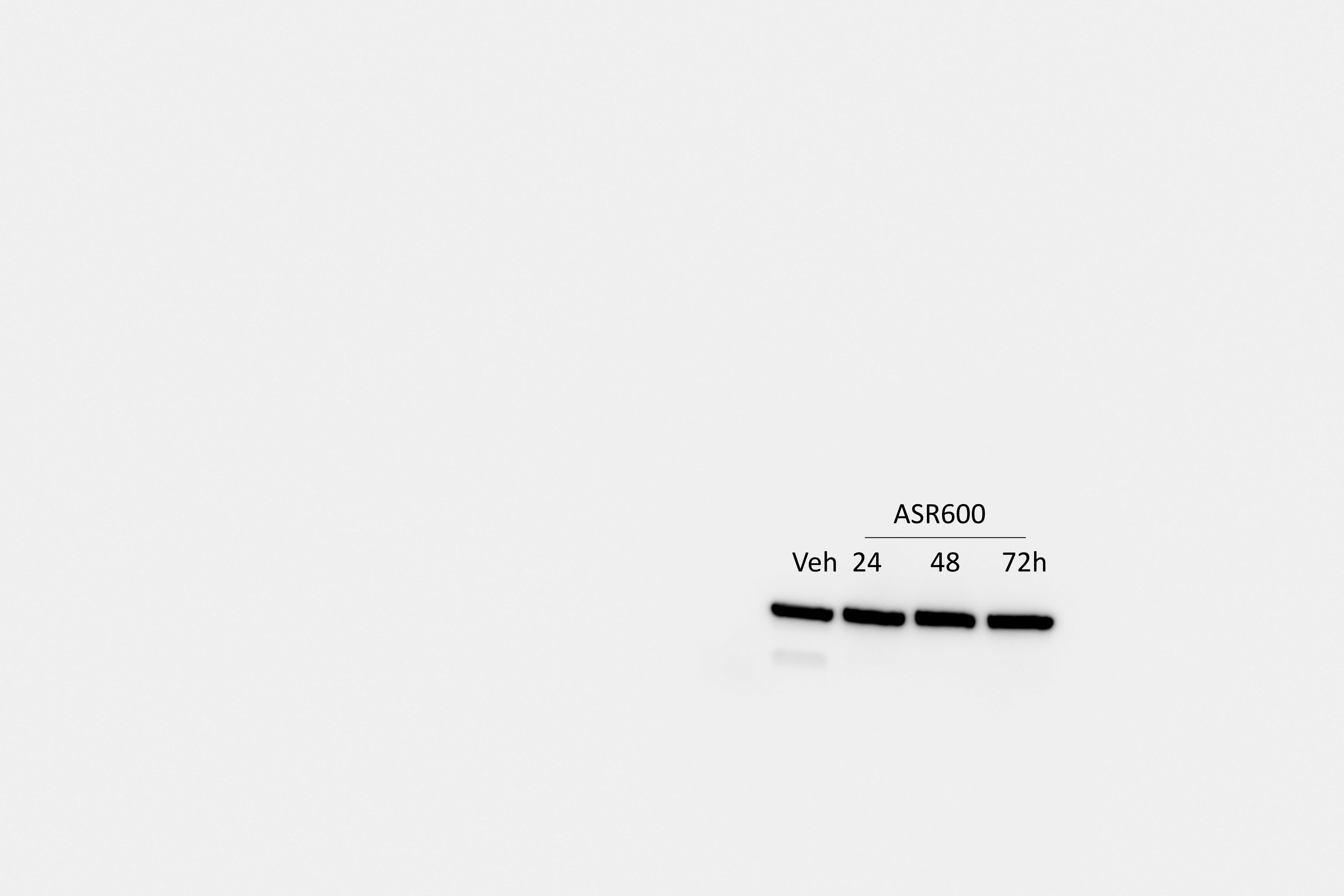

Fig.1f
C4-2B
AR

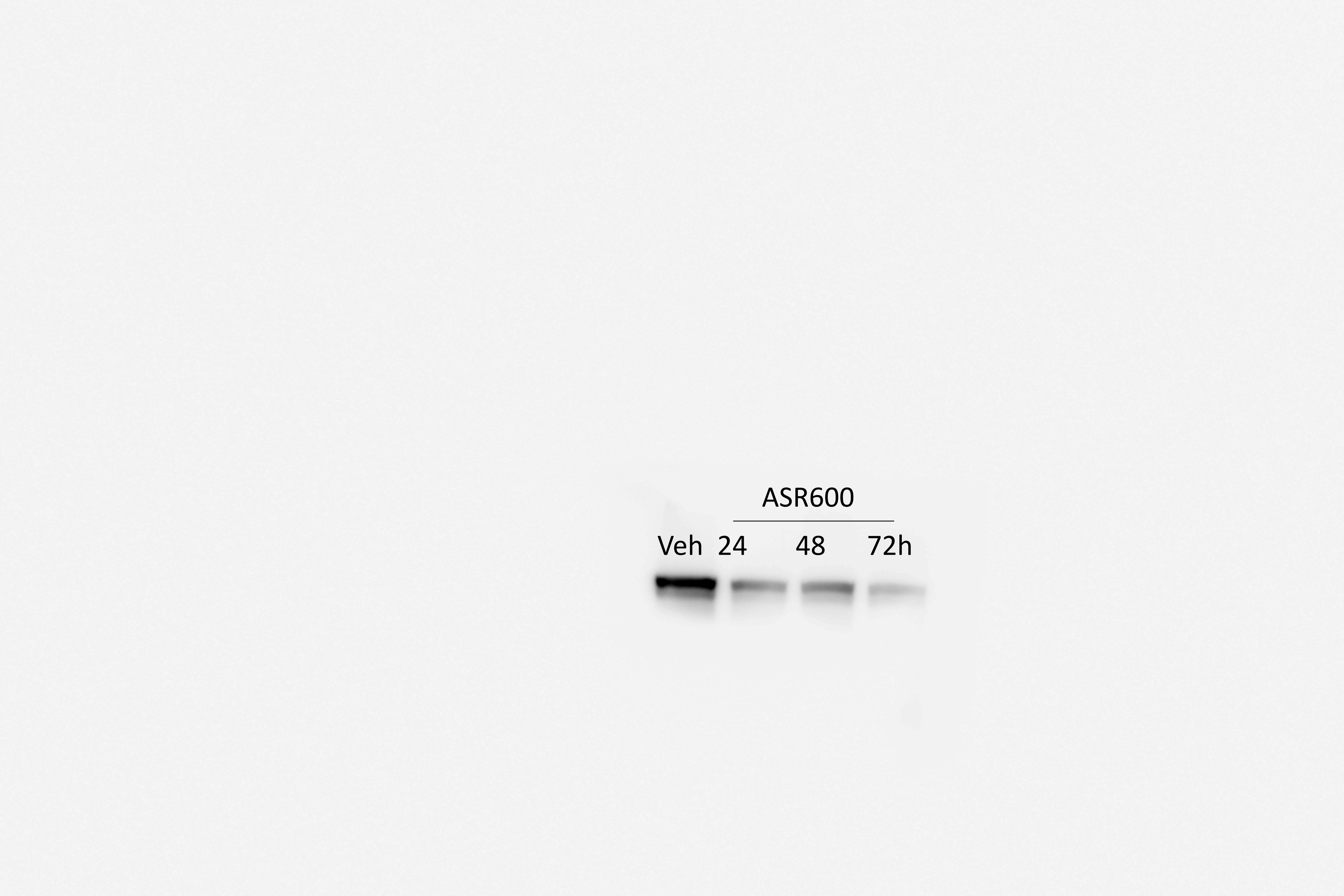

Fig.1f
C4-2B
PSA

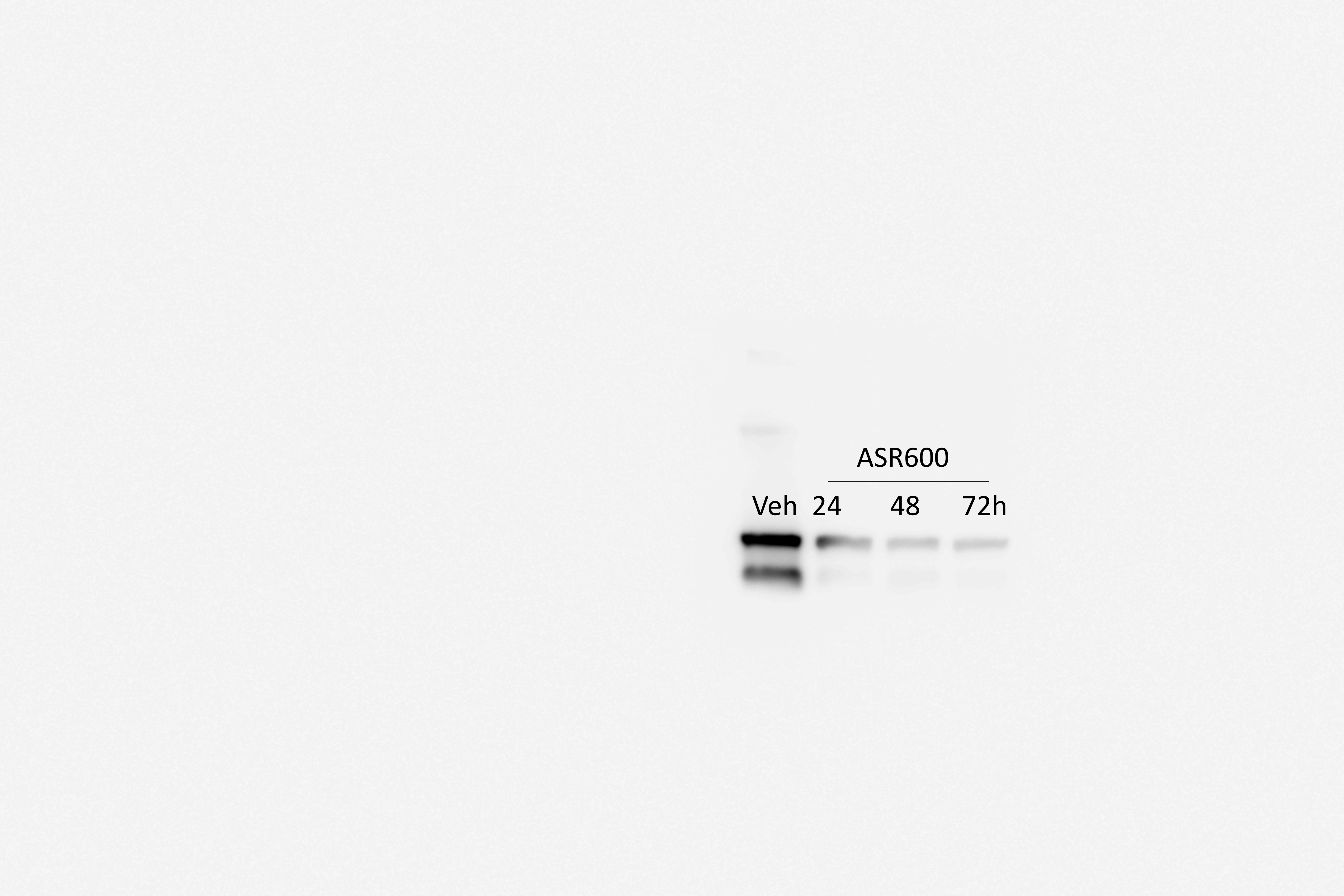

Fig.1f
C4-2B
aCTIN

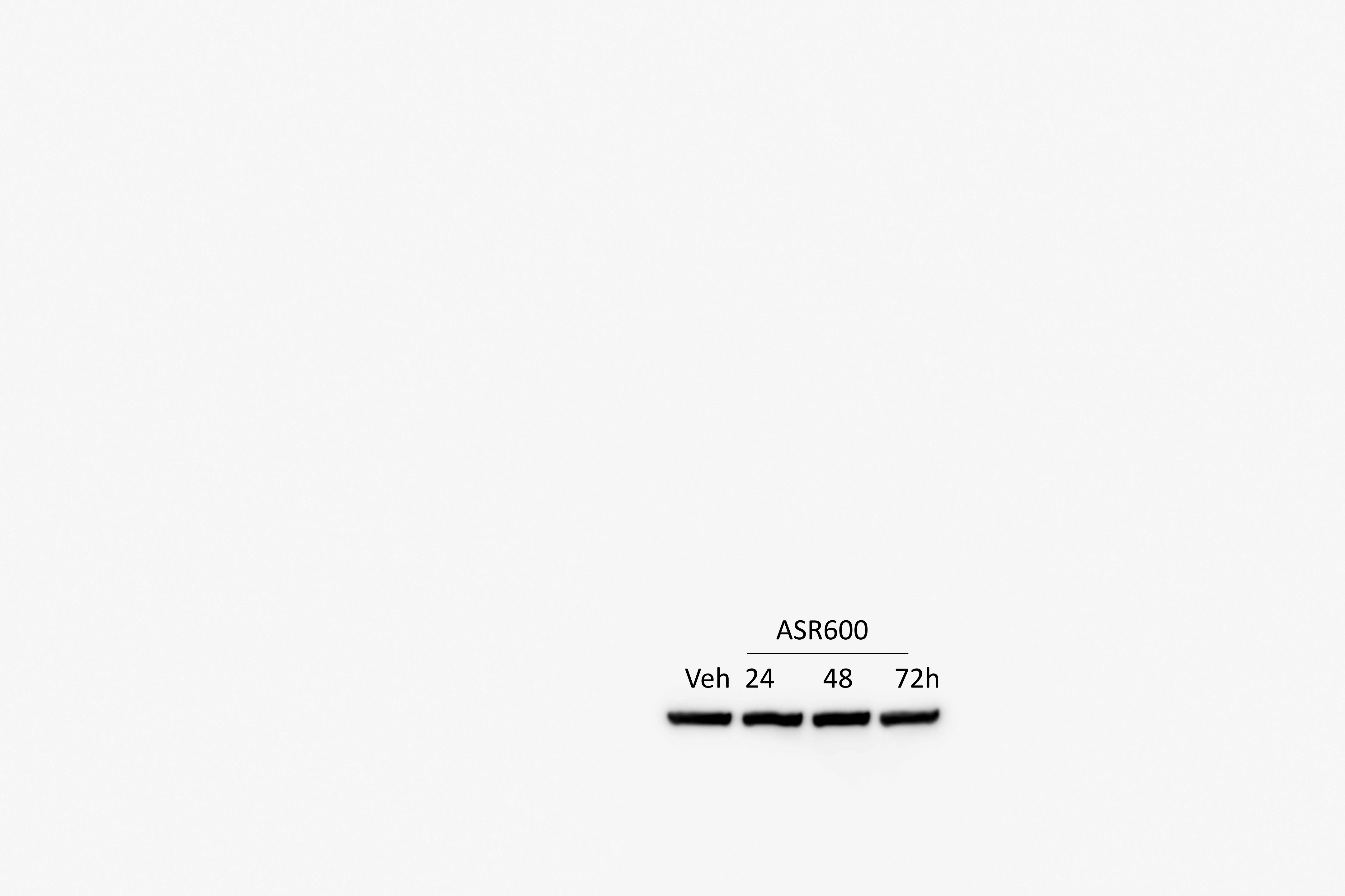

Fig.1g
ENZ-
C4-2B
AR

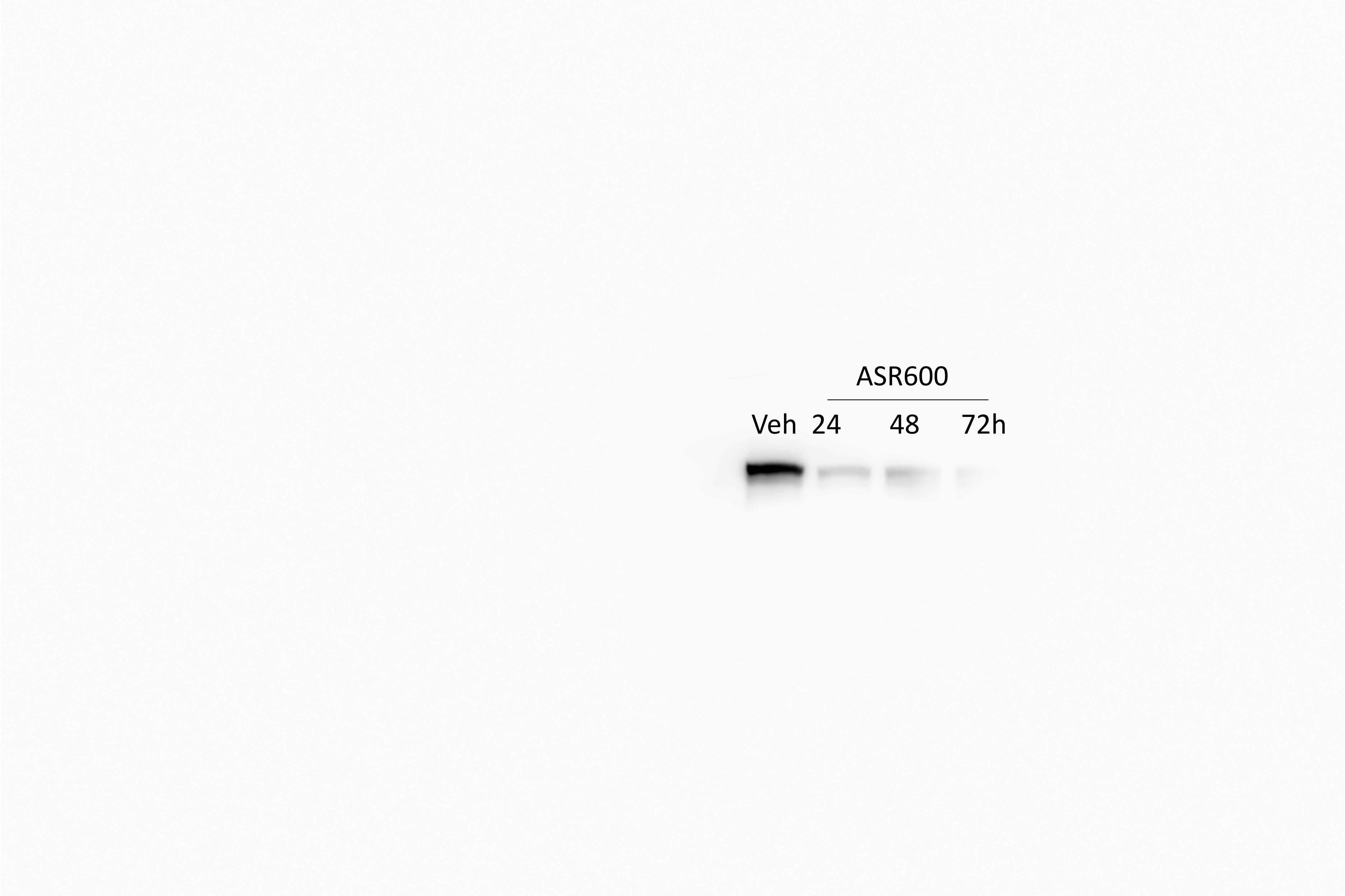

Fig.1f
ENZ-
C4-2B
PSA

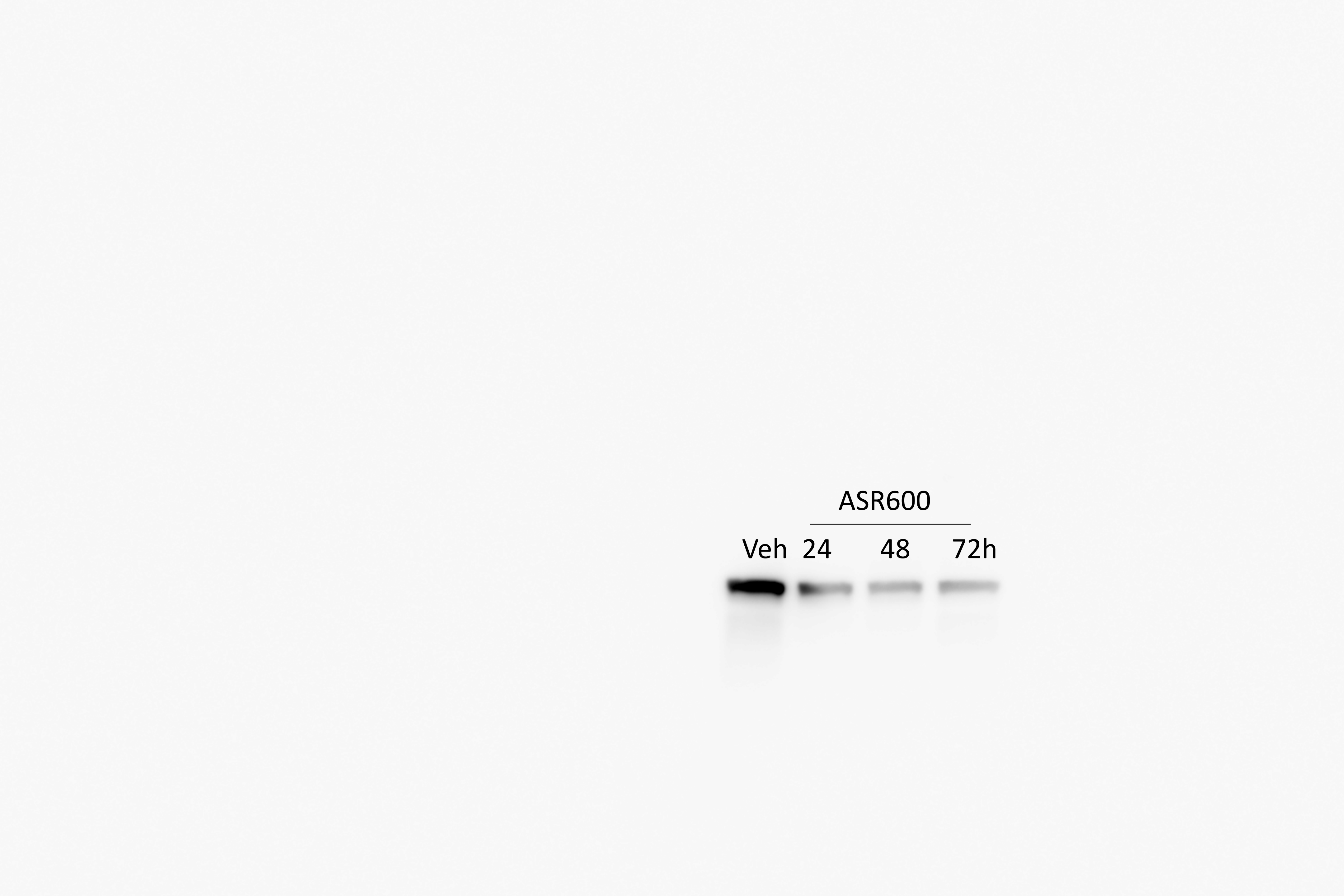

Fig.1g
ENZ-
C4-2B
PSA

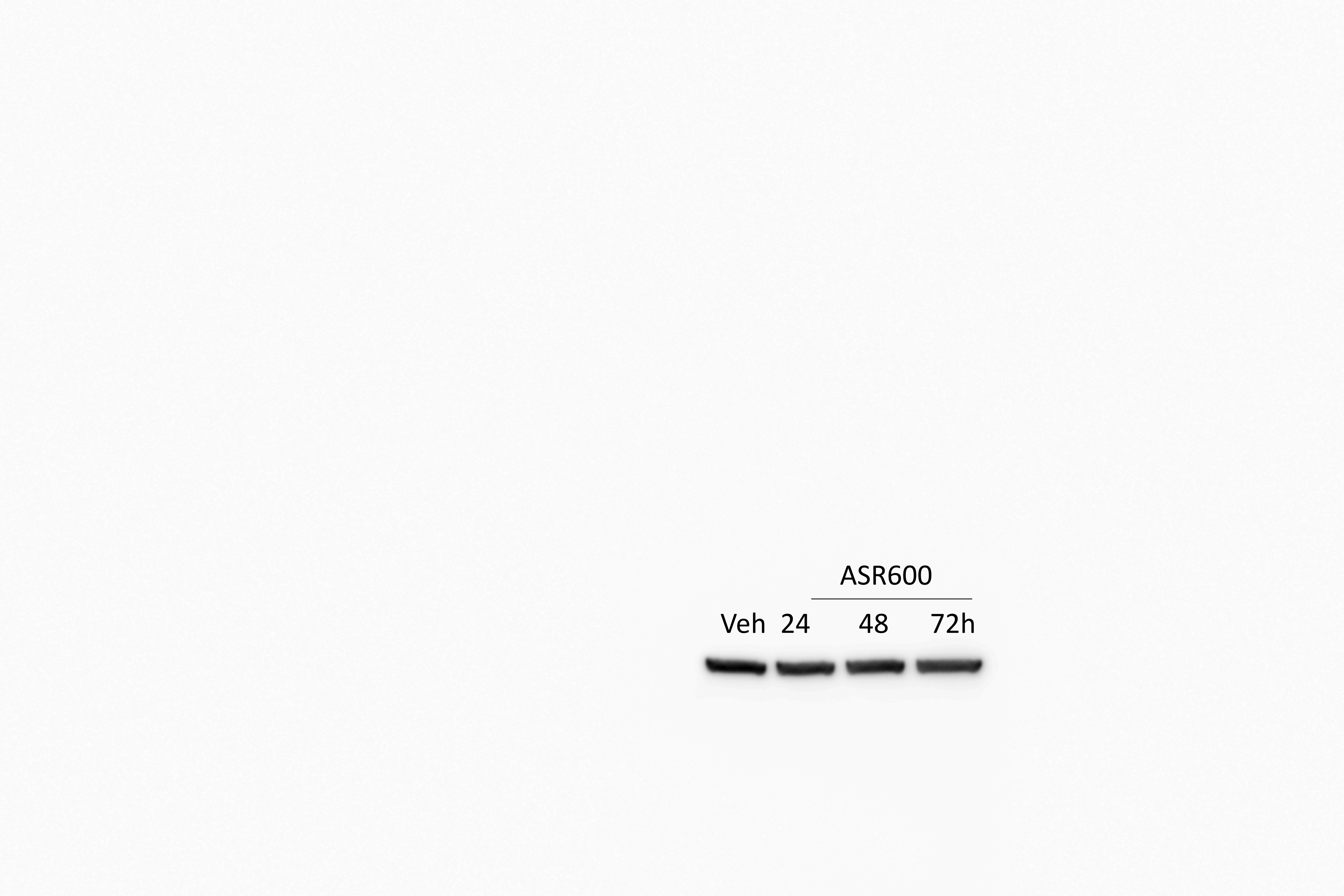

Fig.1H
AR

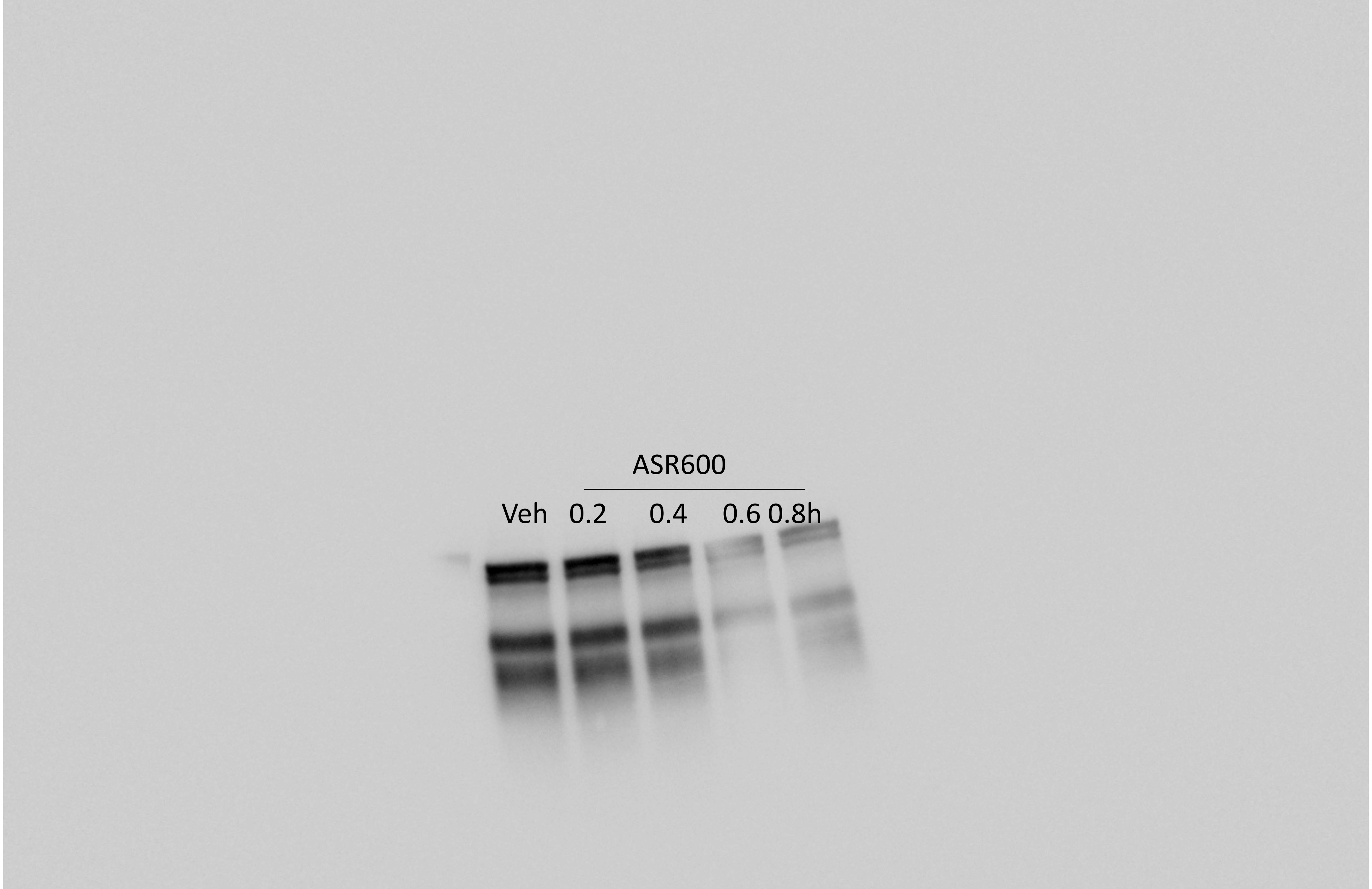

Fig.1H

PSA

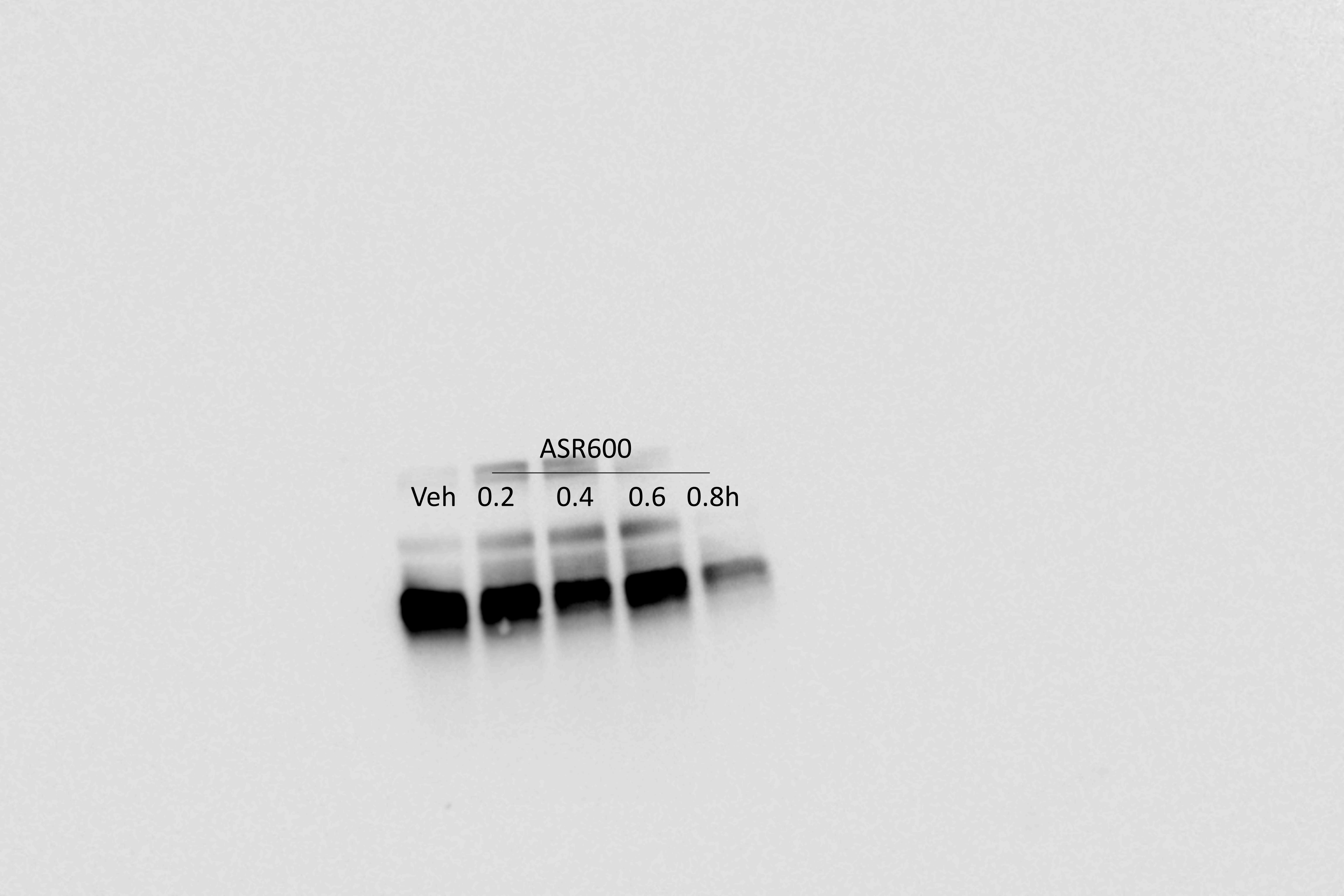

Fig.1H
Actin

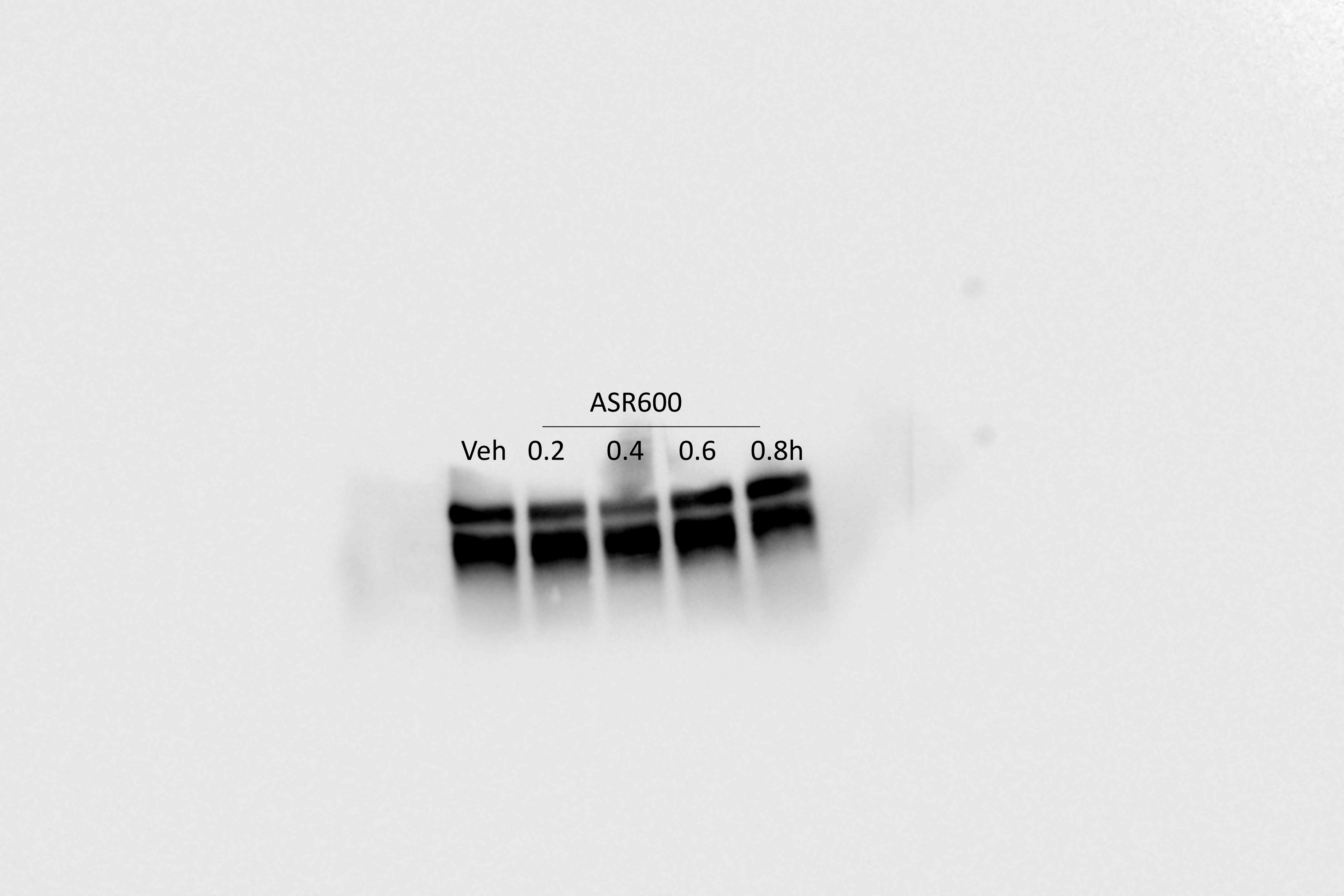

Fig.1i
22RV1
AR

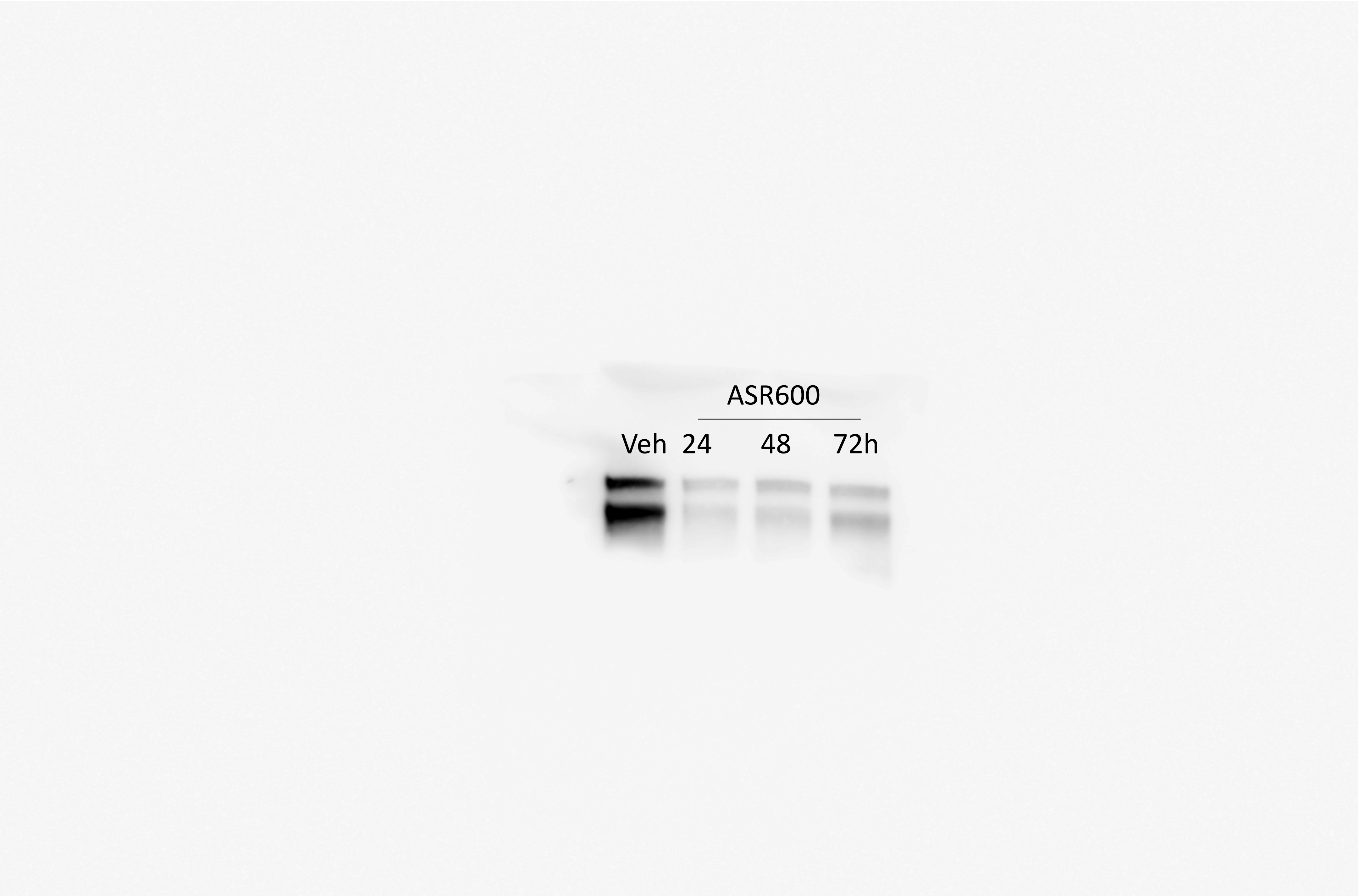

Fig.1i
22RV1
PSA

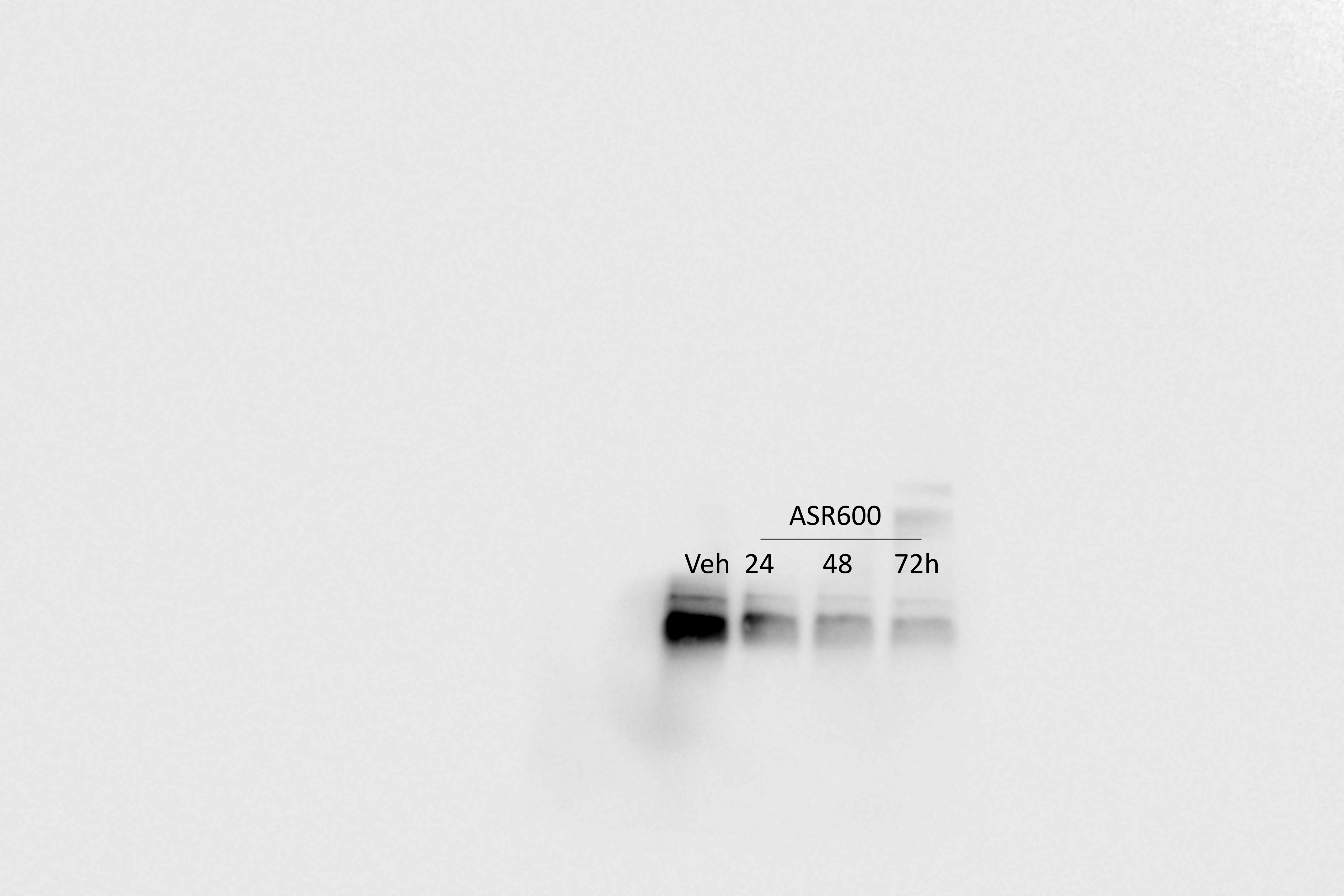

Fig.1i
22RV1
Actin

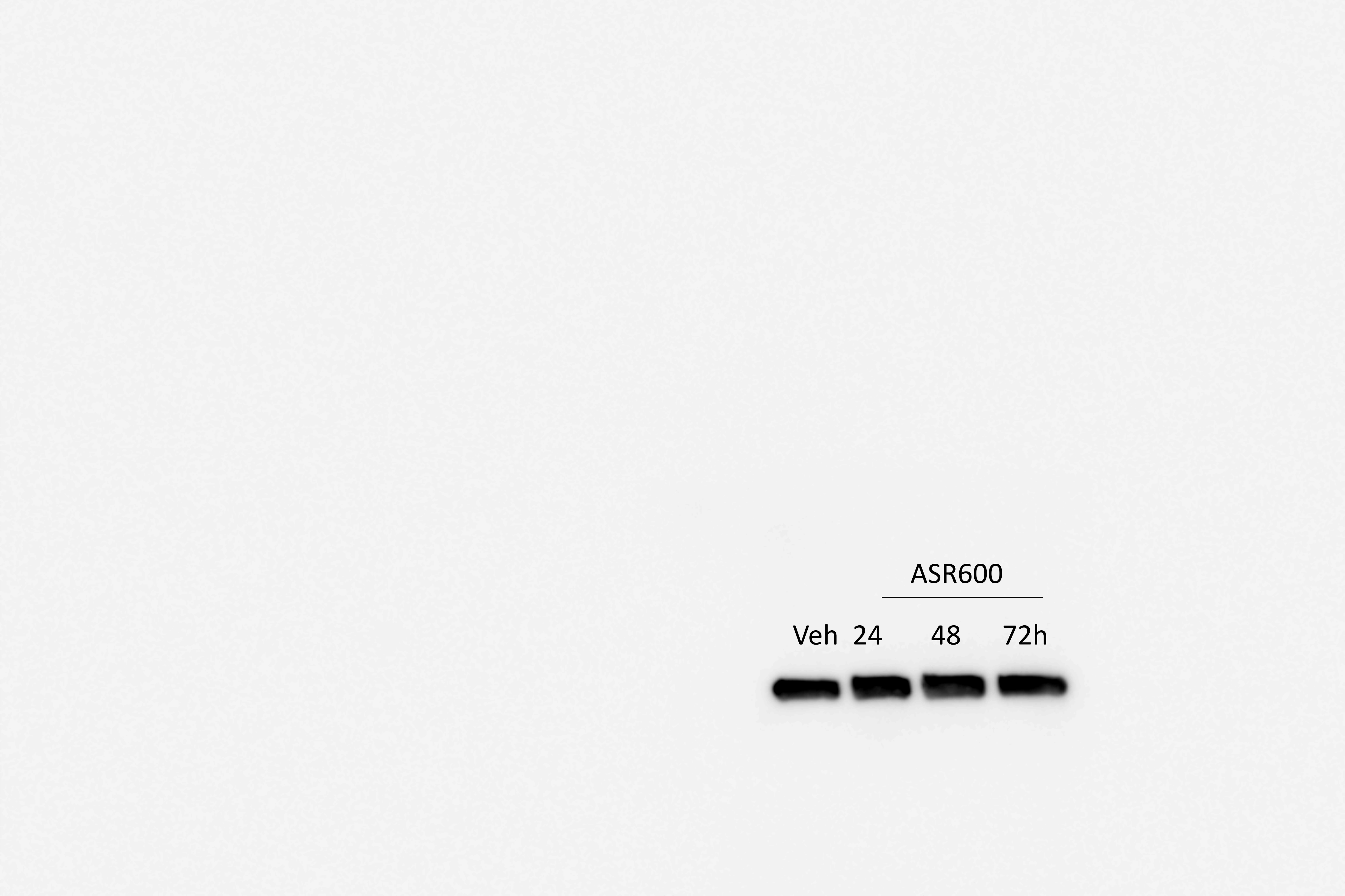

Fig.1j
VCaP
AR

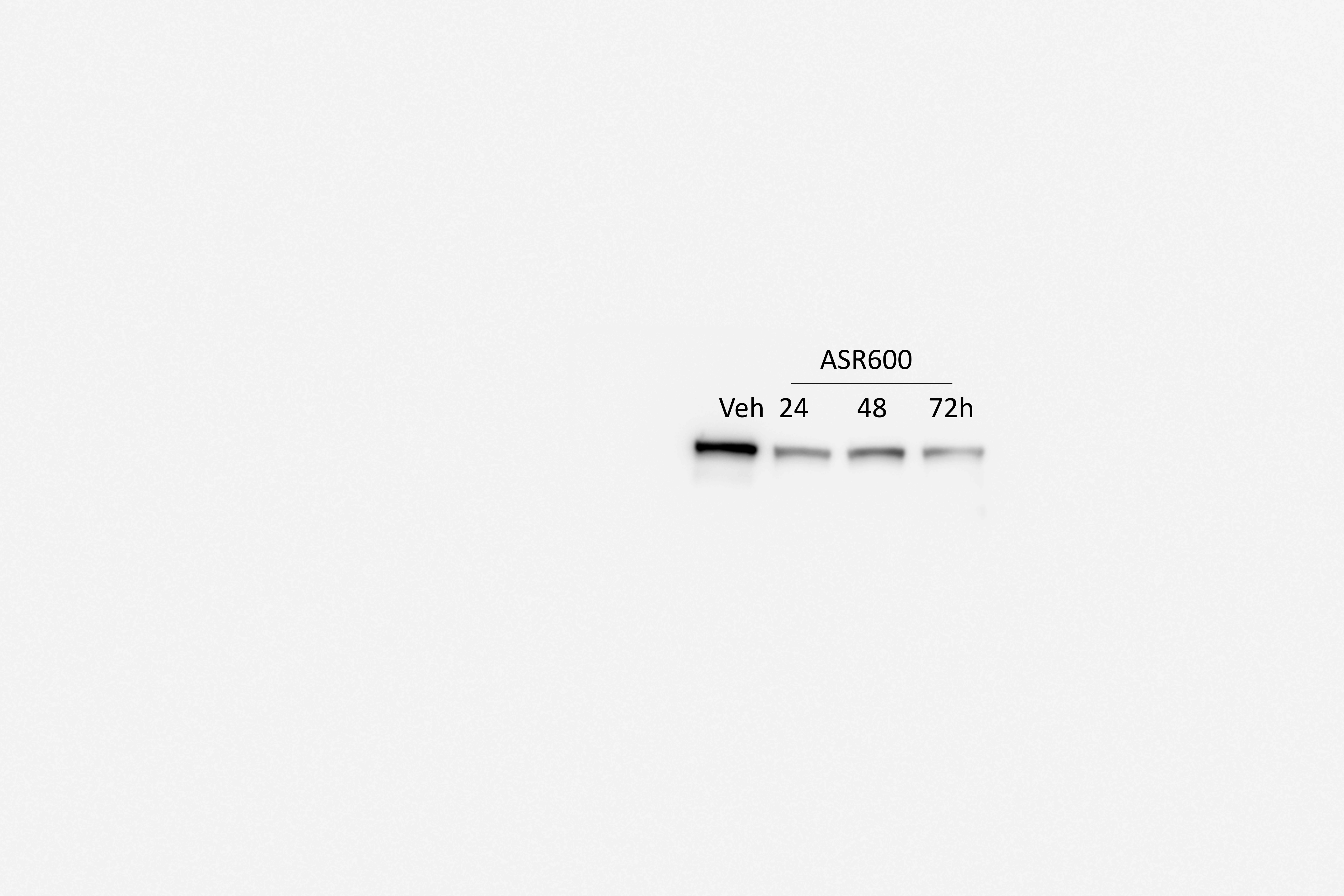

Fig.1j
VCaP
ARV7

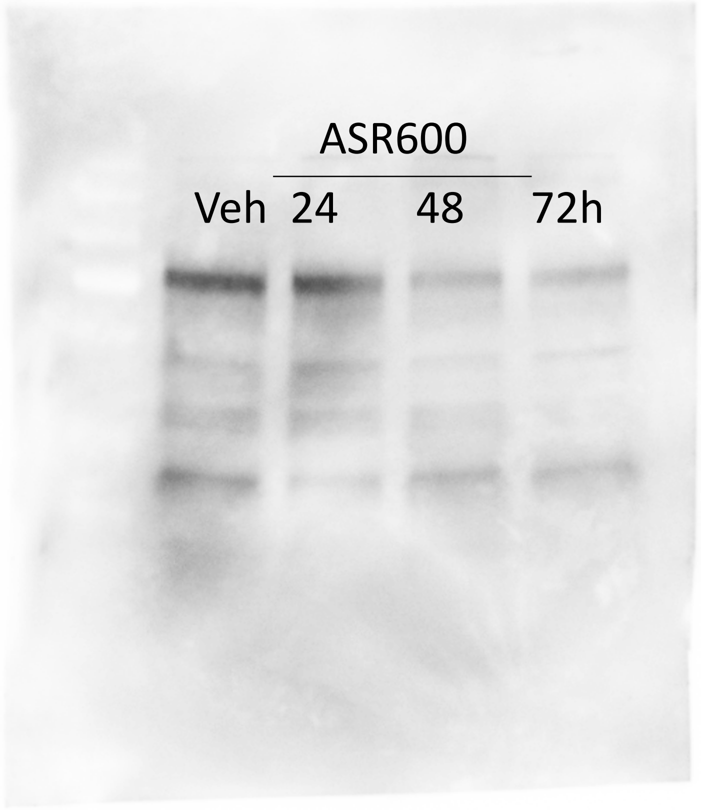

Fig.1j
VCaP
PSA

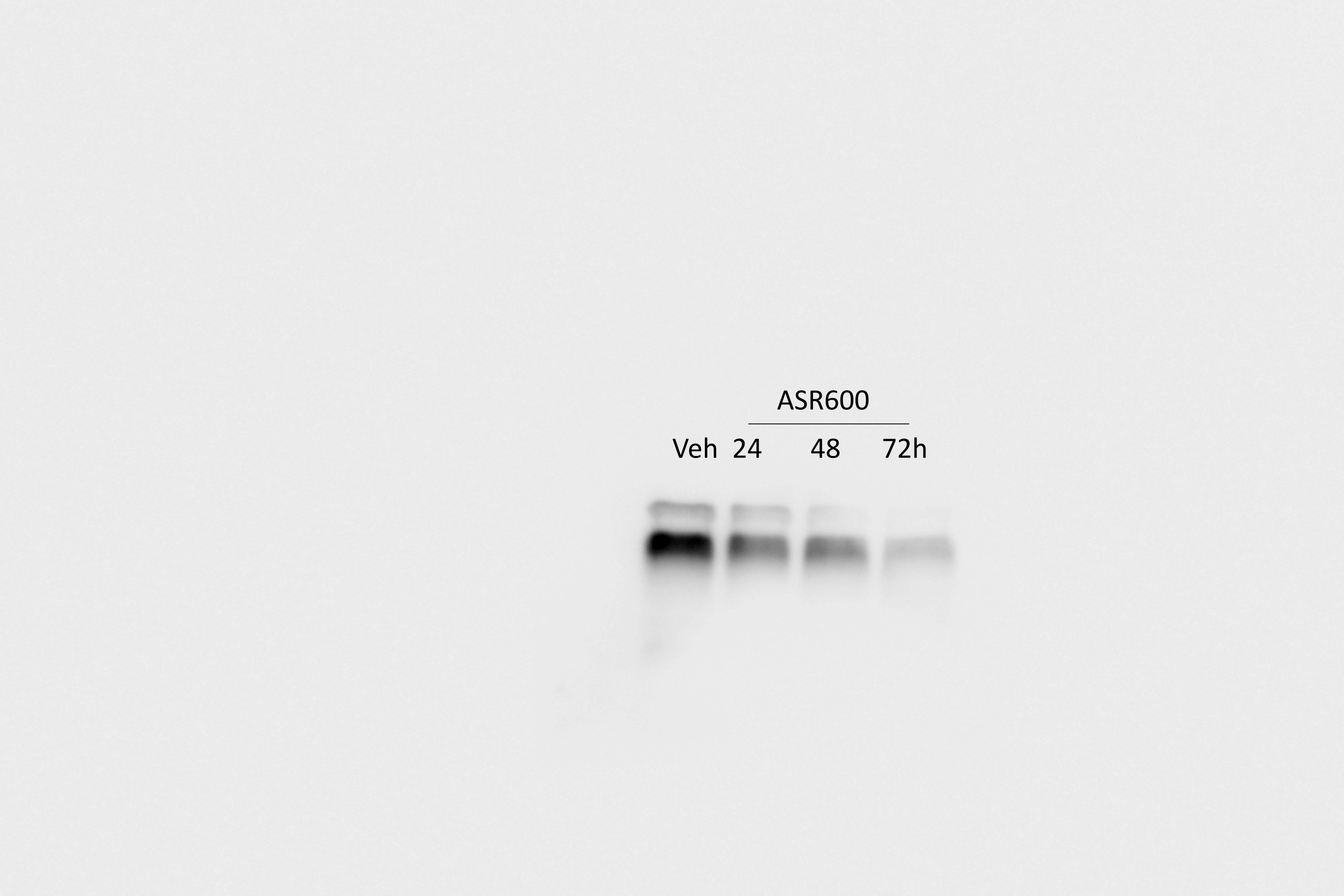

Fig.1j
VCaP
Actin

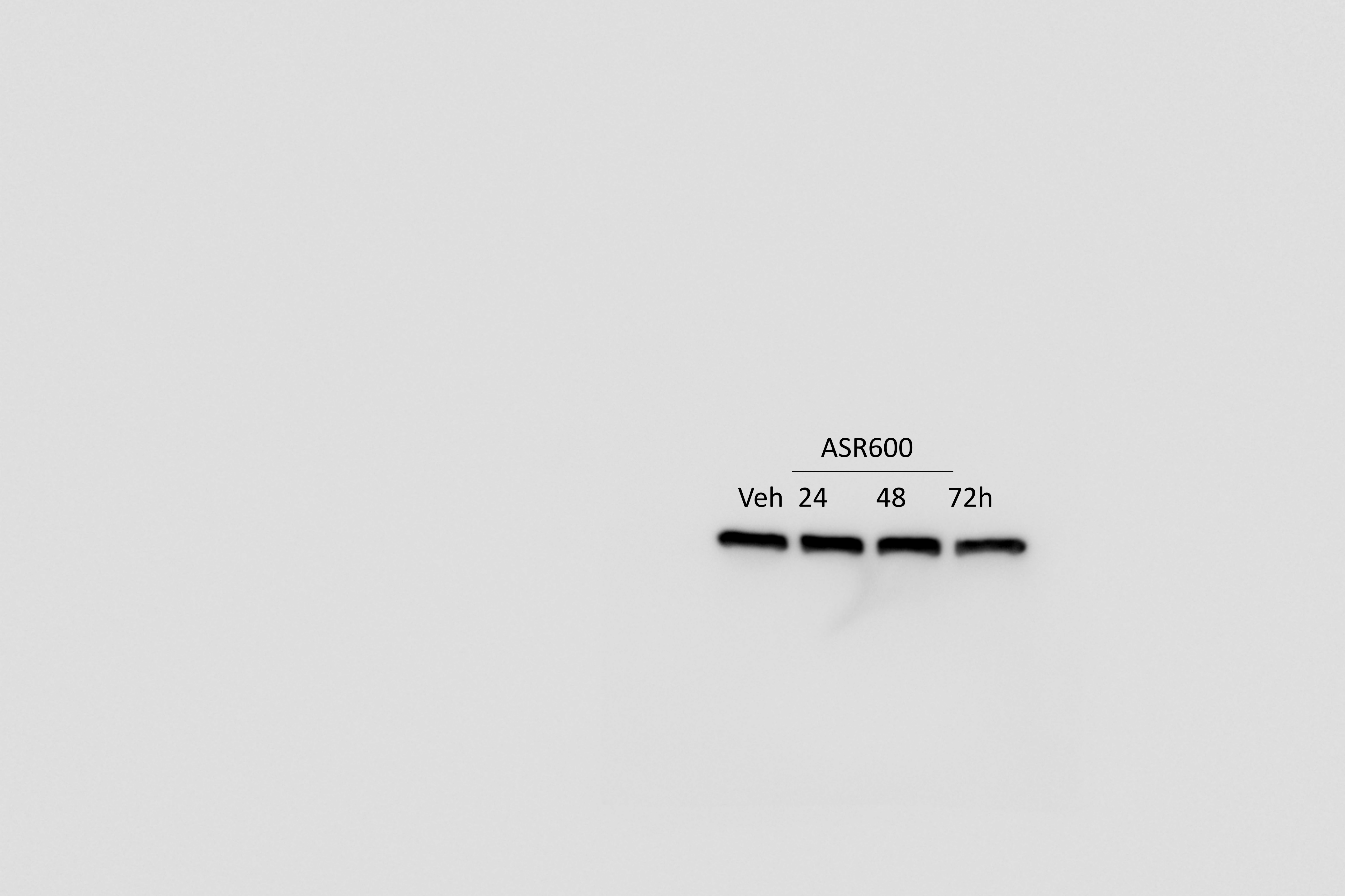

Fig.2a
22RV1
DHT
AR

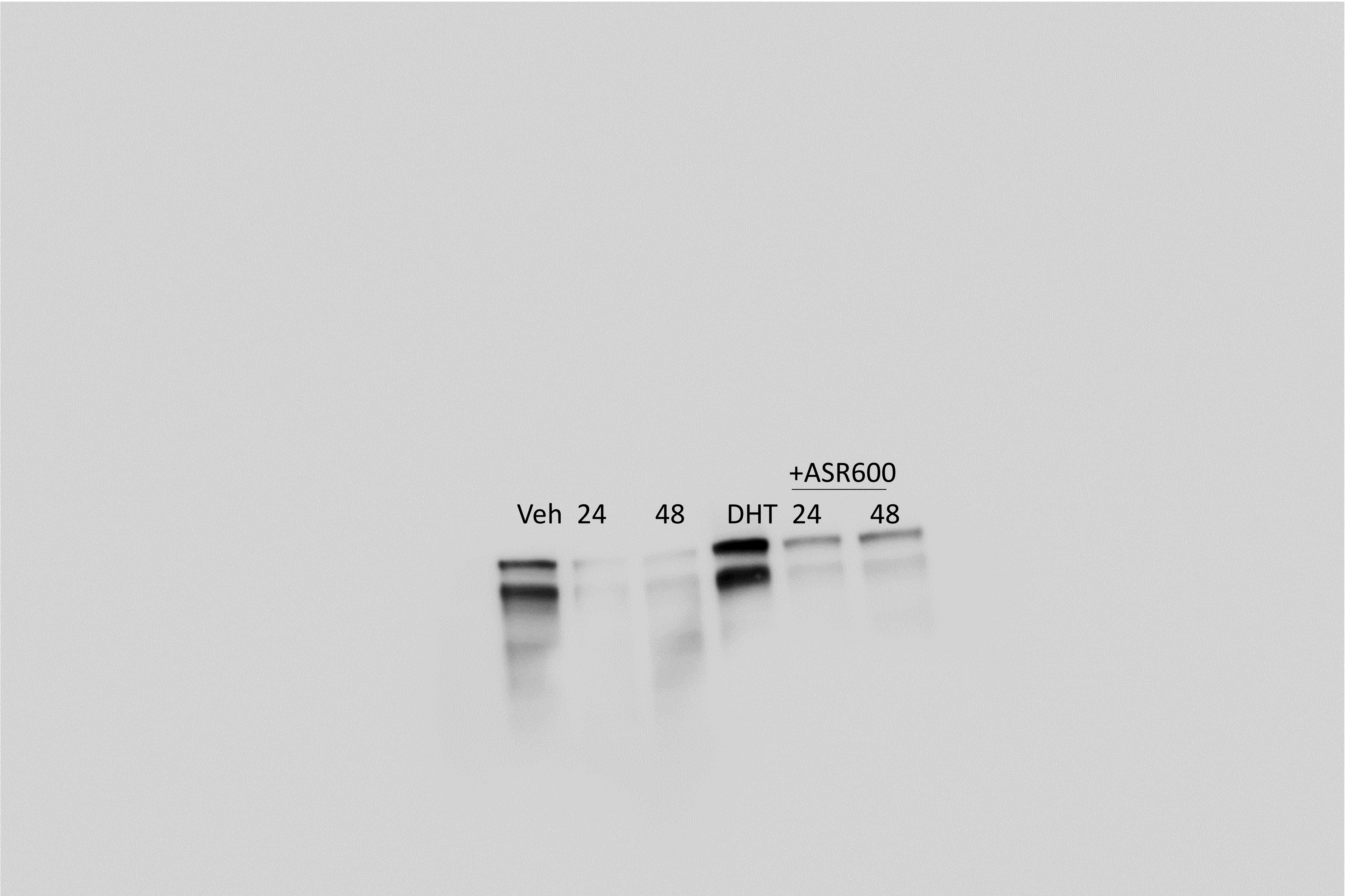

Fig.2a
22RV1
DHT
PSA

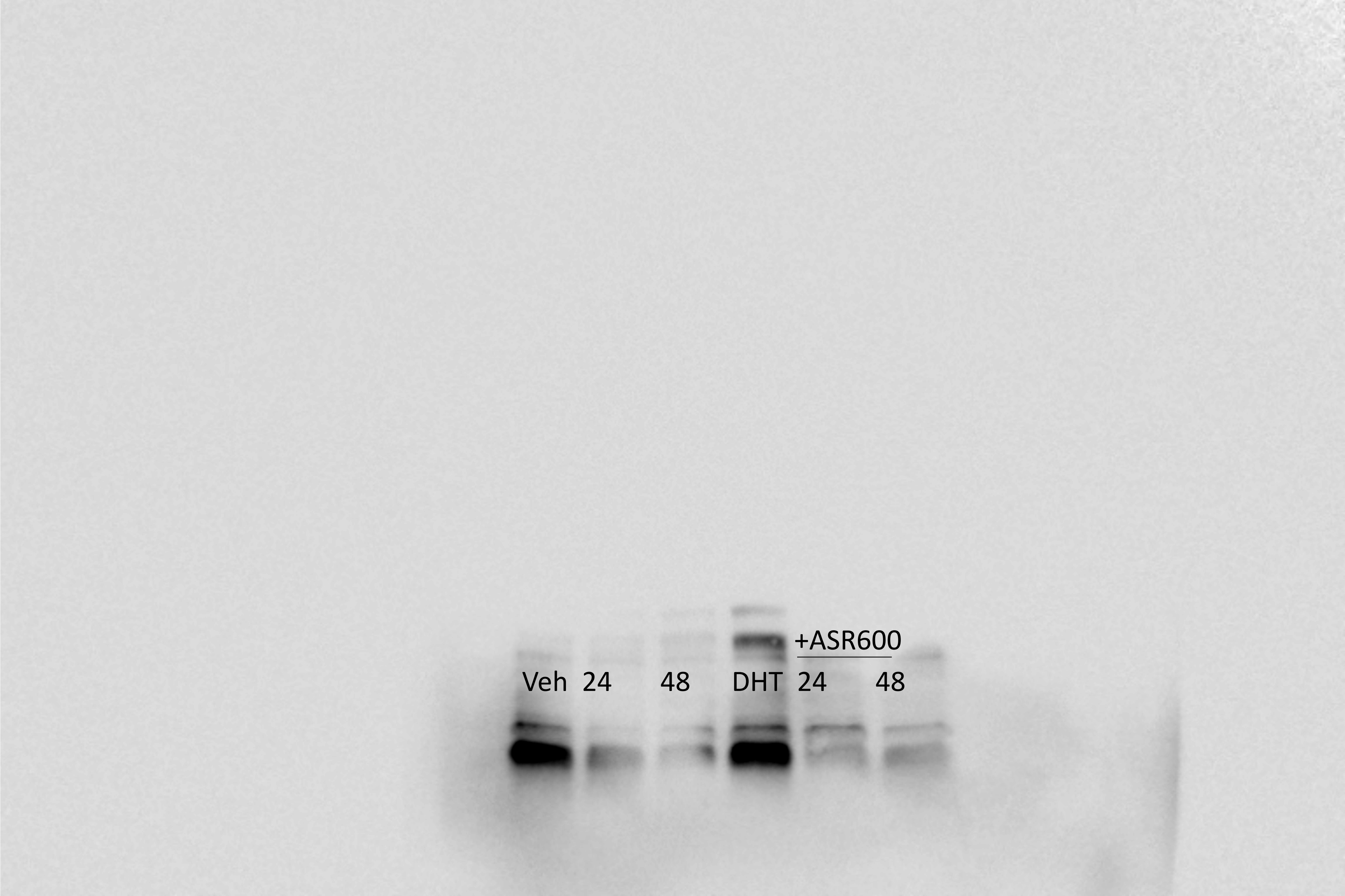

Fig.2a
22RV1
DHT
ACtin

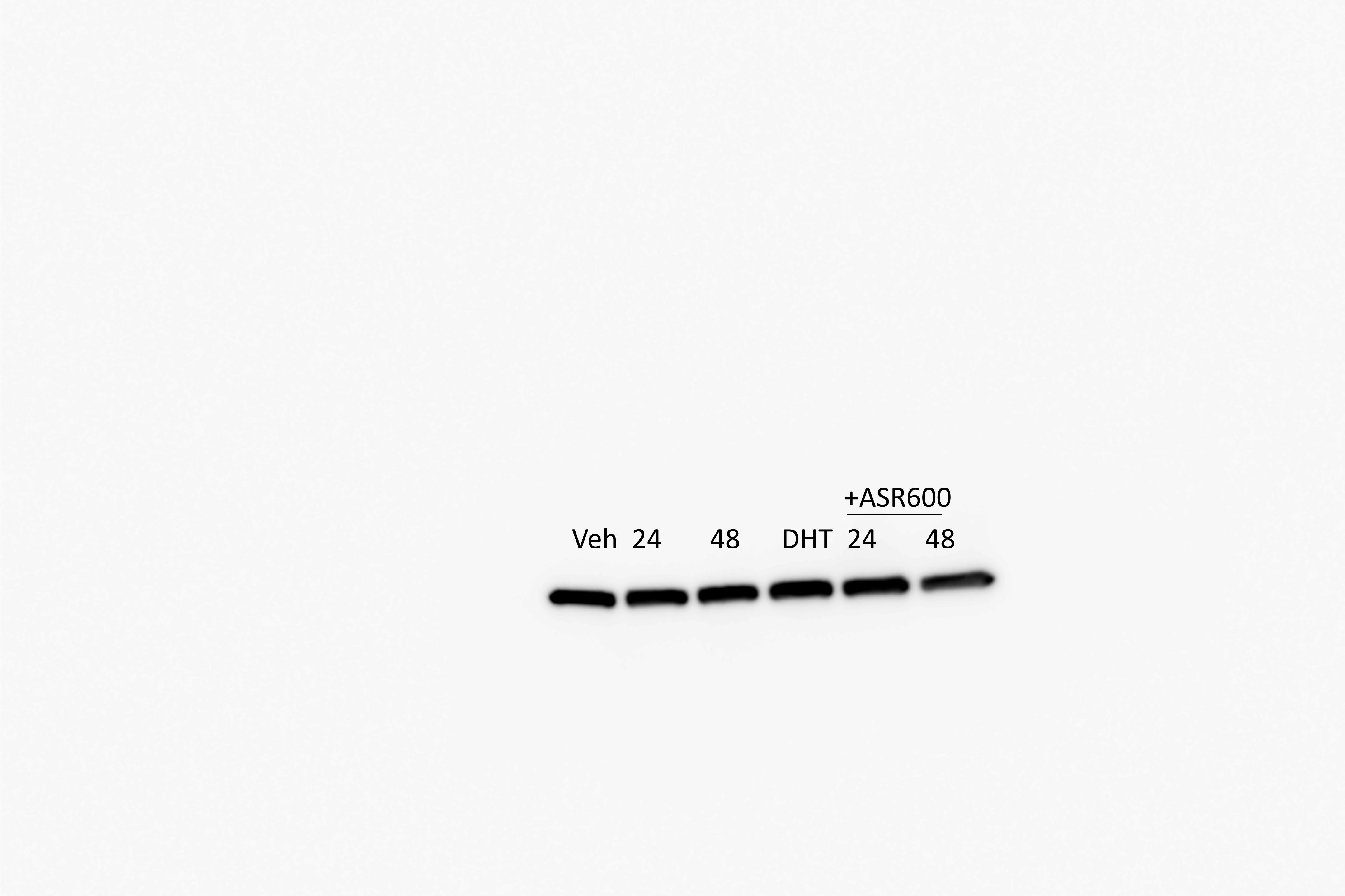

Fig.2b
C4-2B
DHT
AR

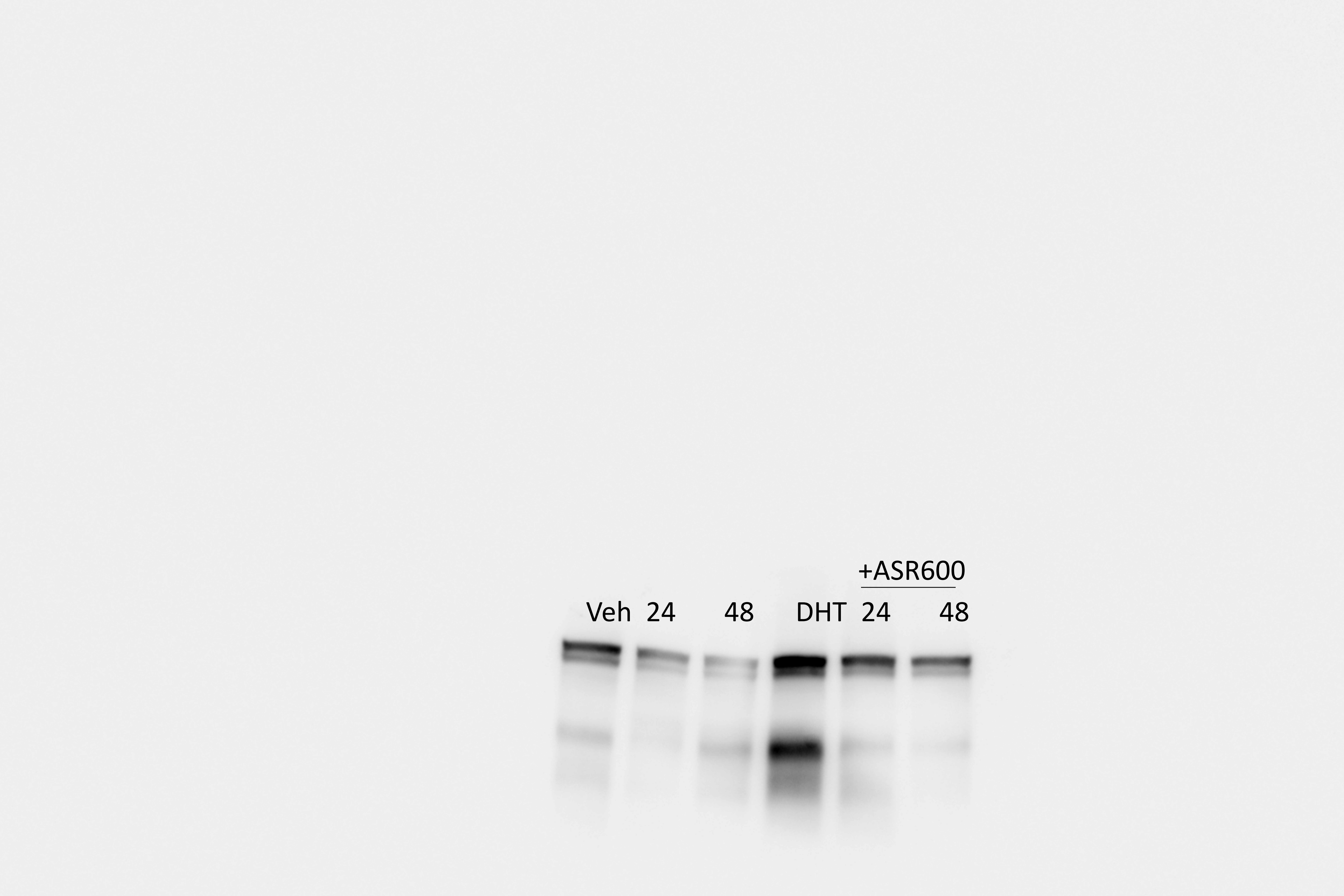

Fig.2B
C4-2B
DHT
PSA

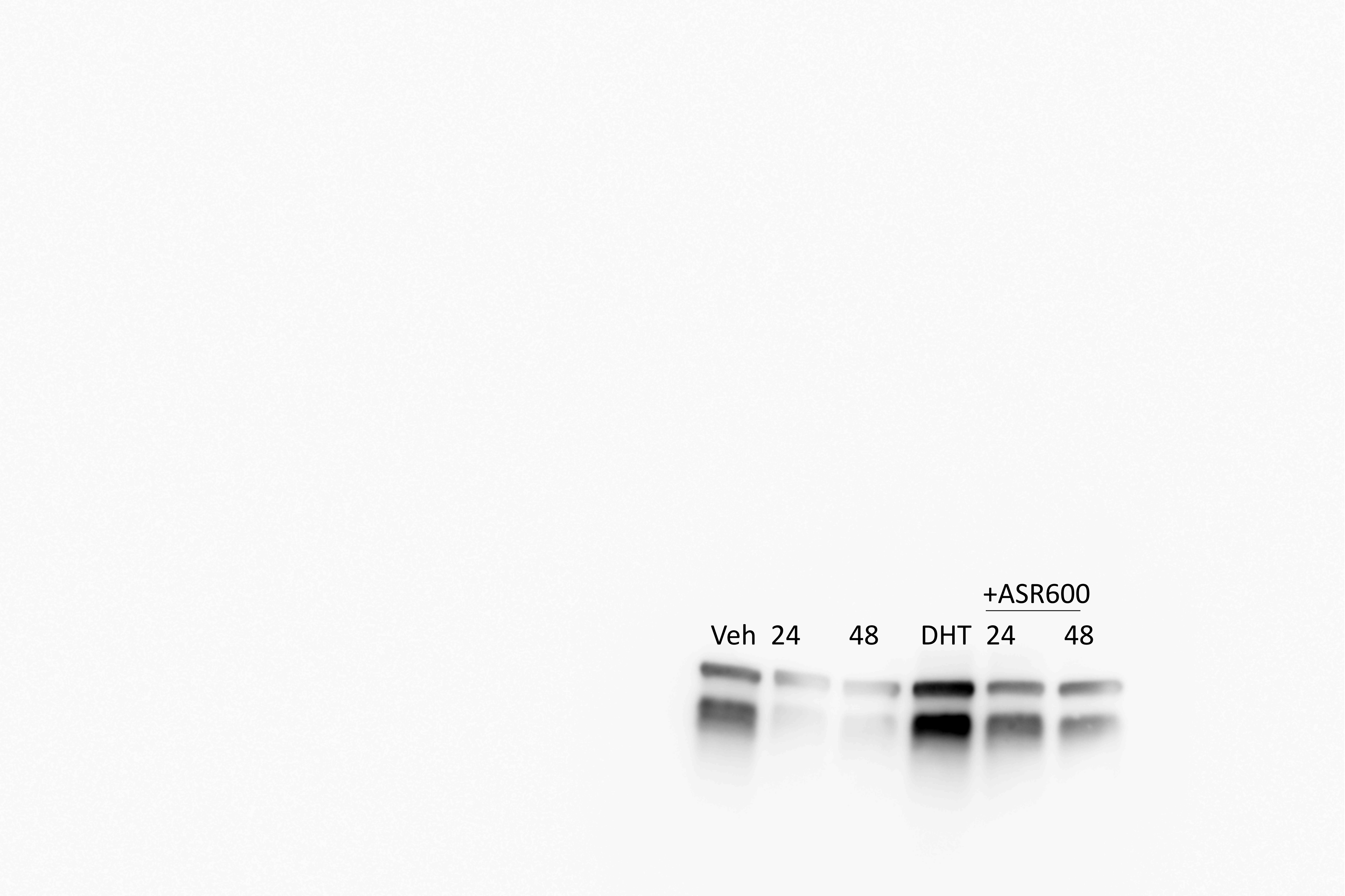

Fig.2b
C4-2B
DHT
Actin

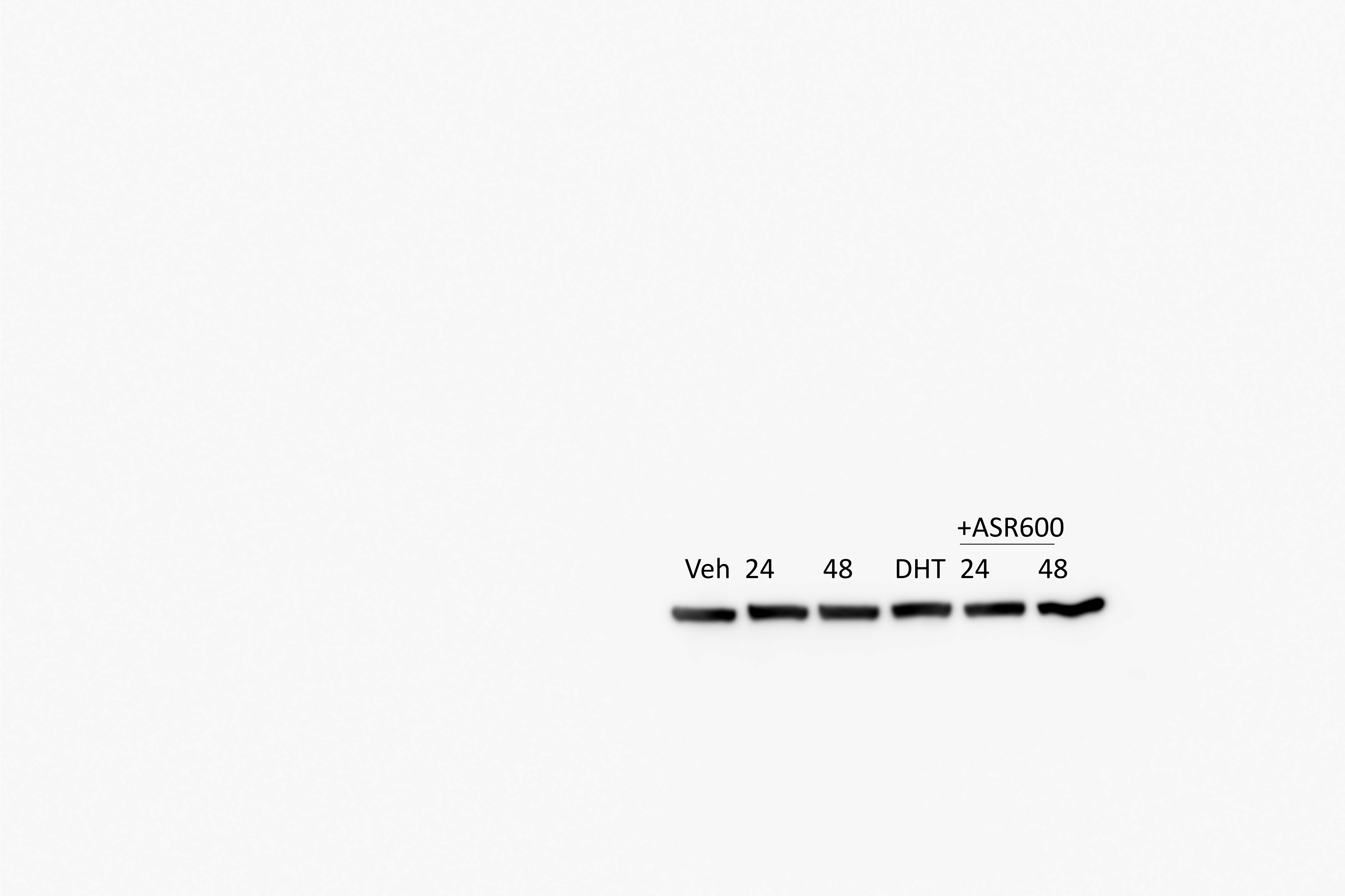

Fig.2C
hAR

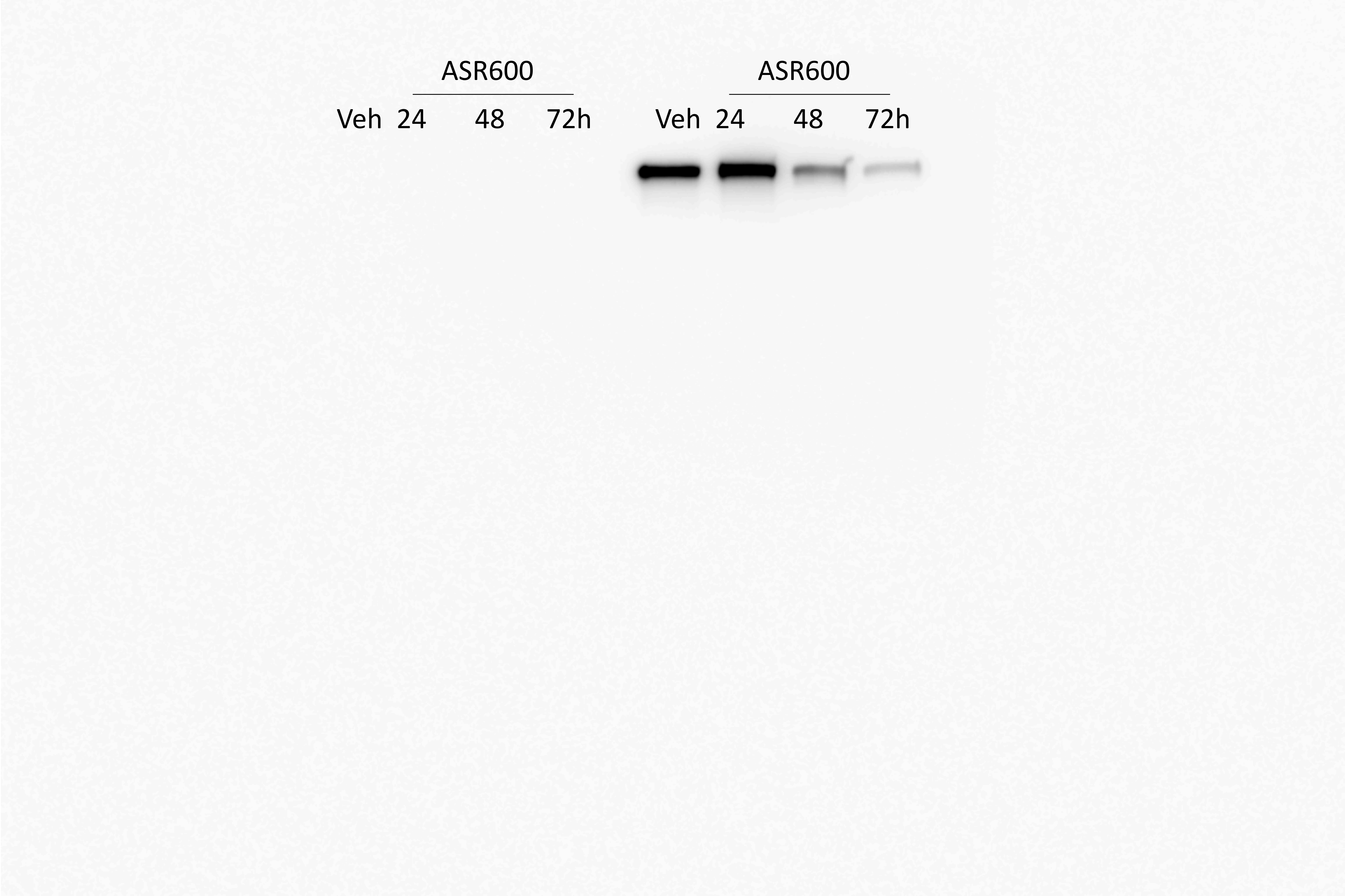

Fig.1e
hAR
Actin

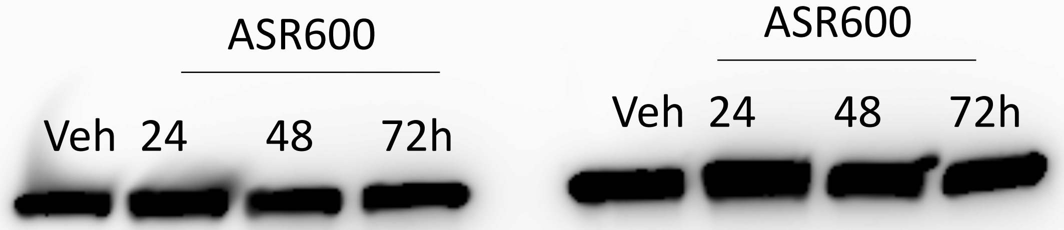

Fig.2d
hAR

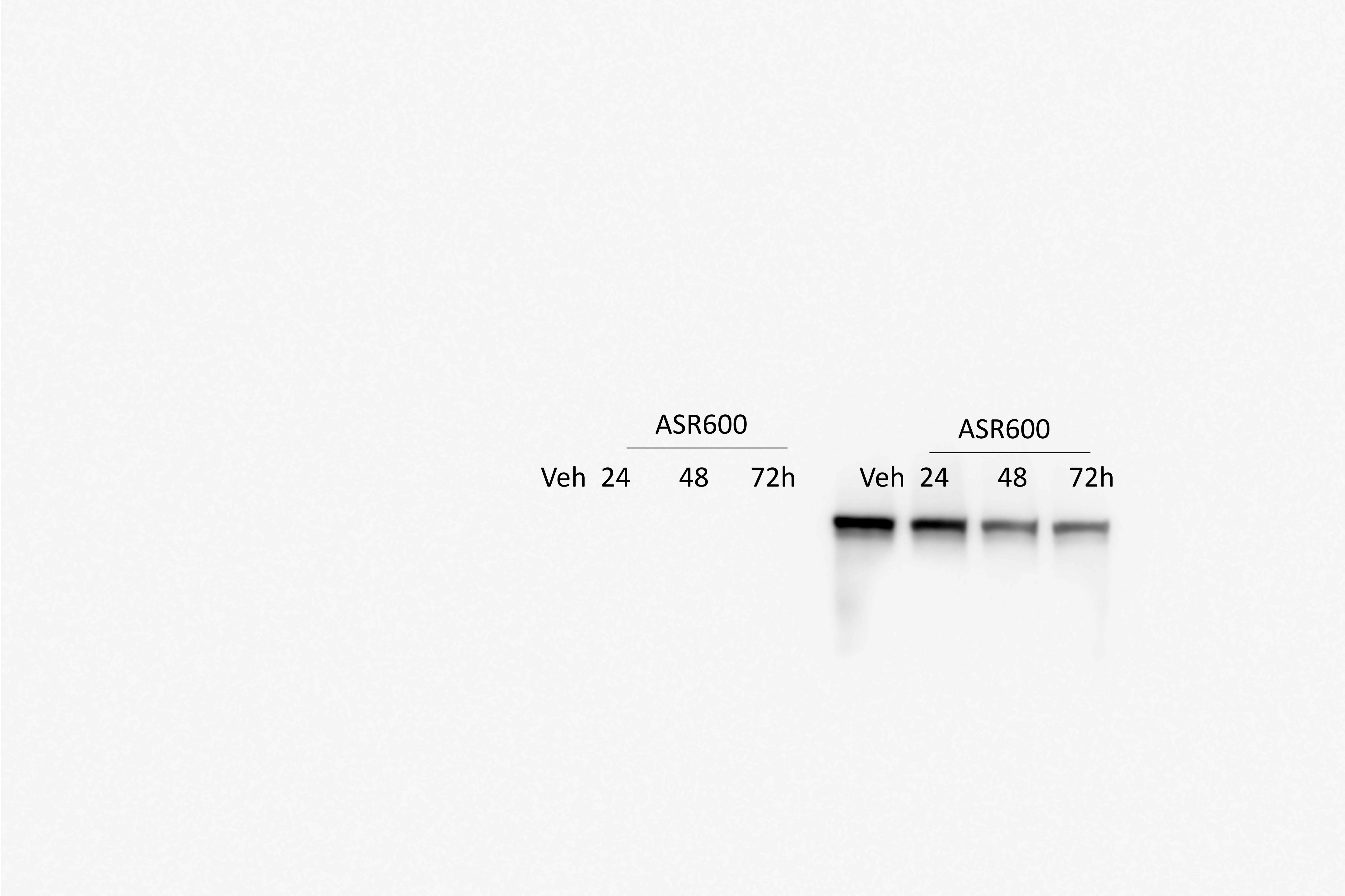

Fig.2d
hAR
Actin

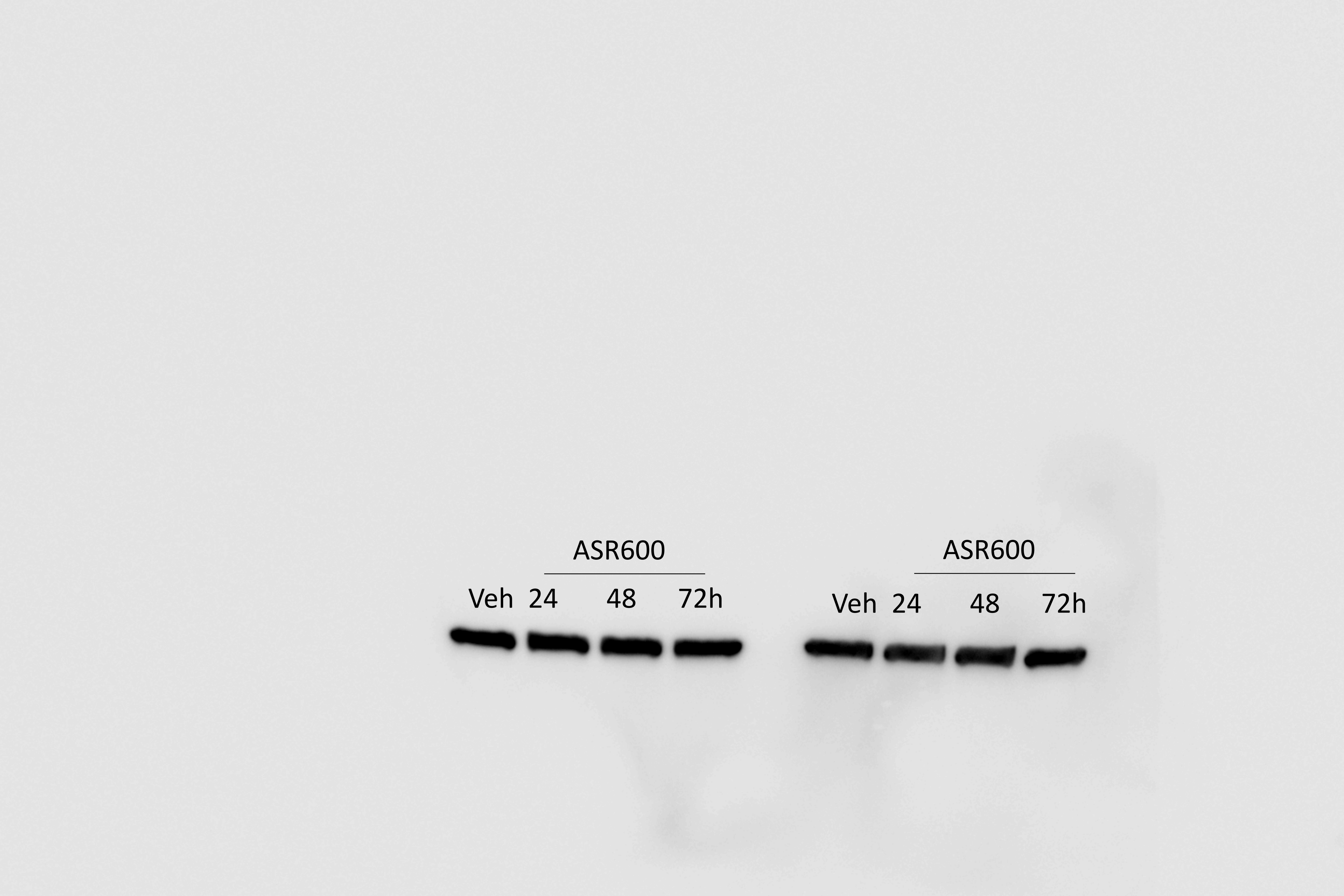

Fig.4a
AR-V7

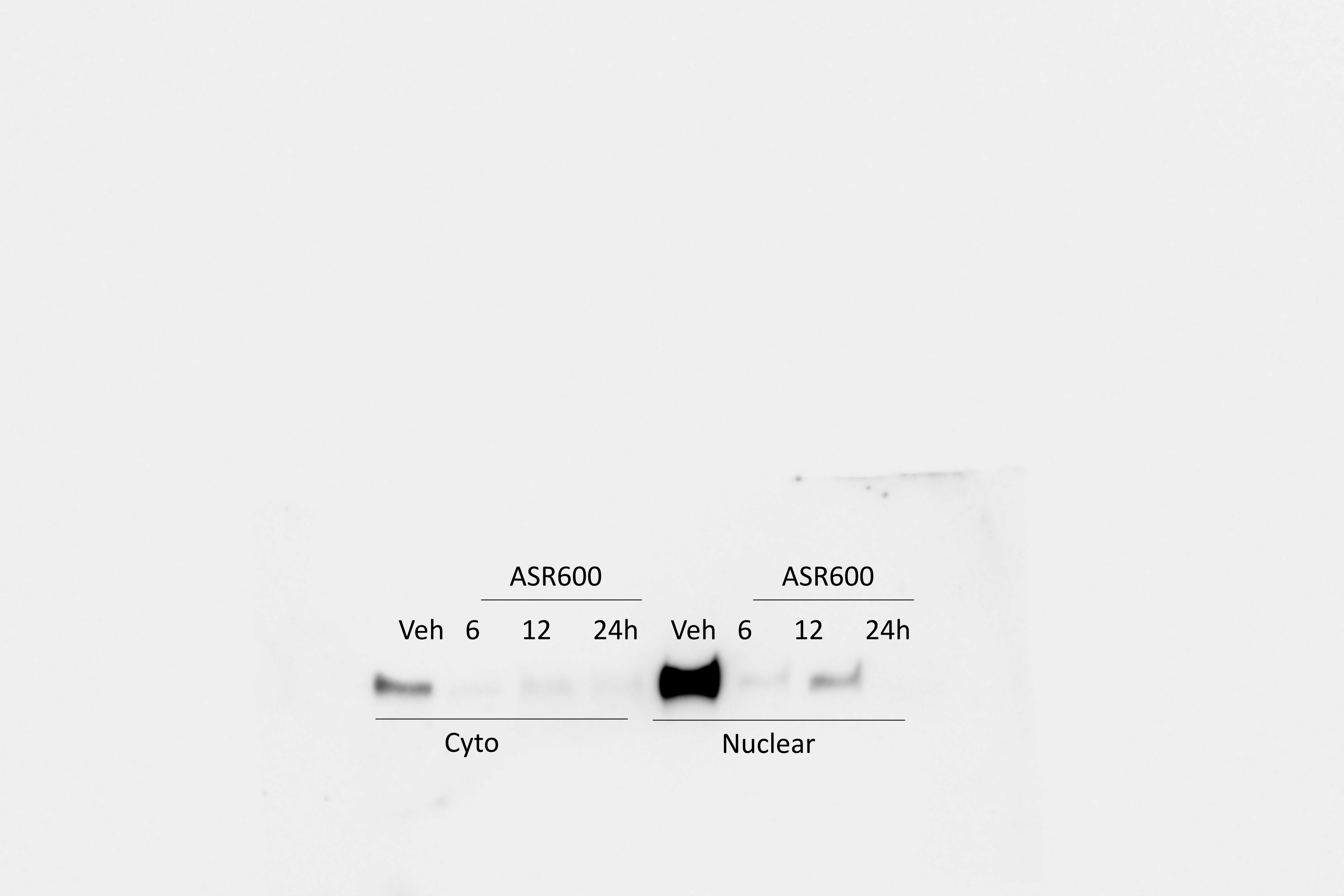

Fig.4a
Lamin A

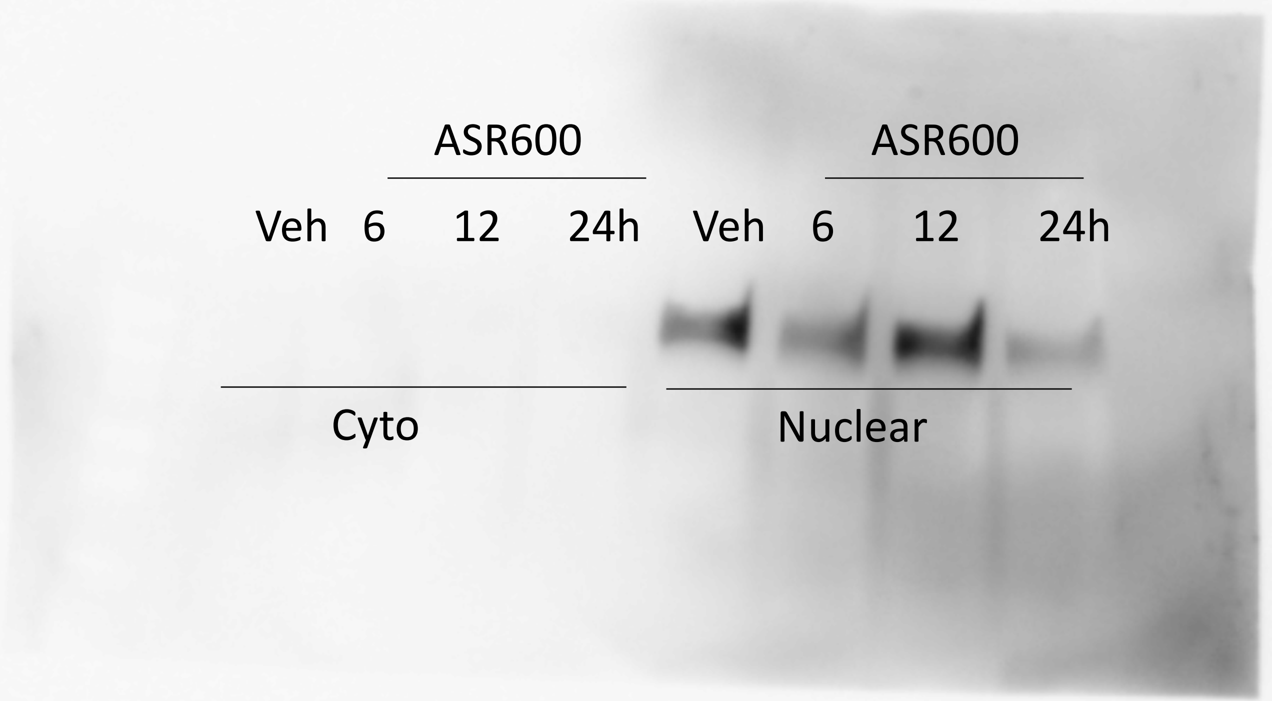

Fig.4a
Actin

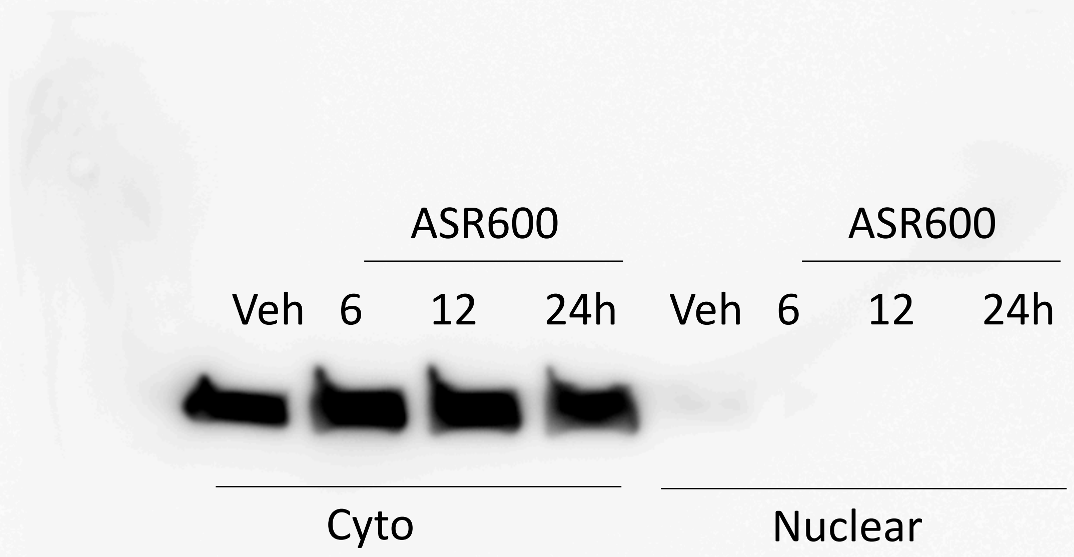

Fig.4d
AR-V7

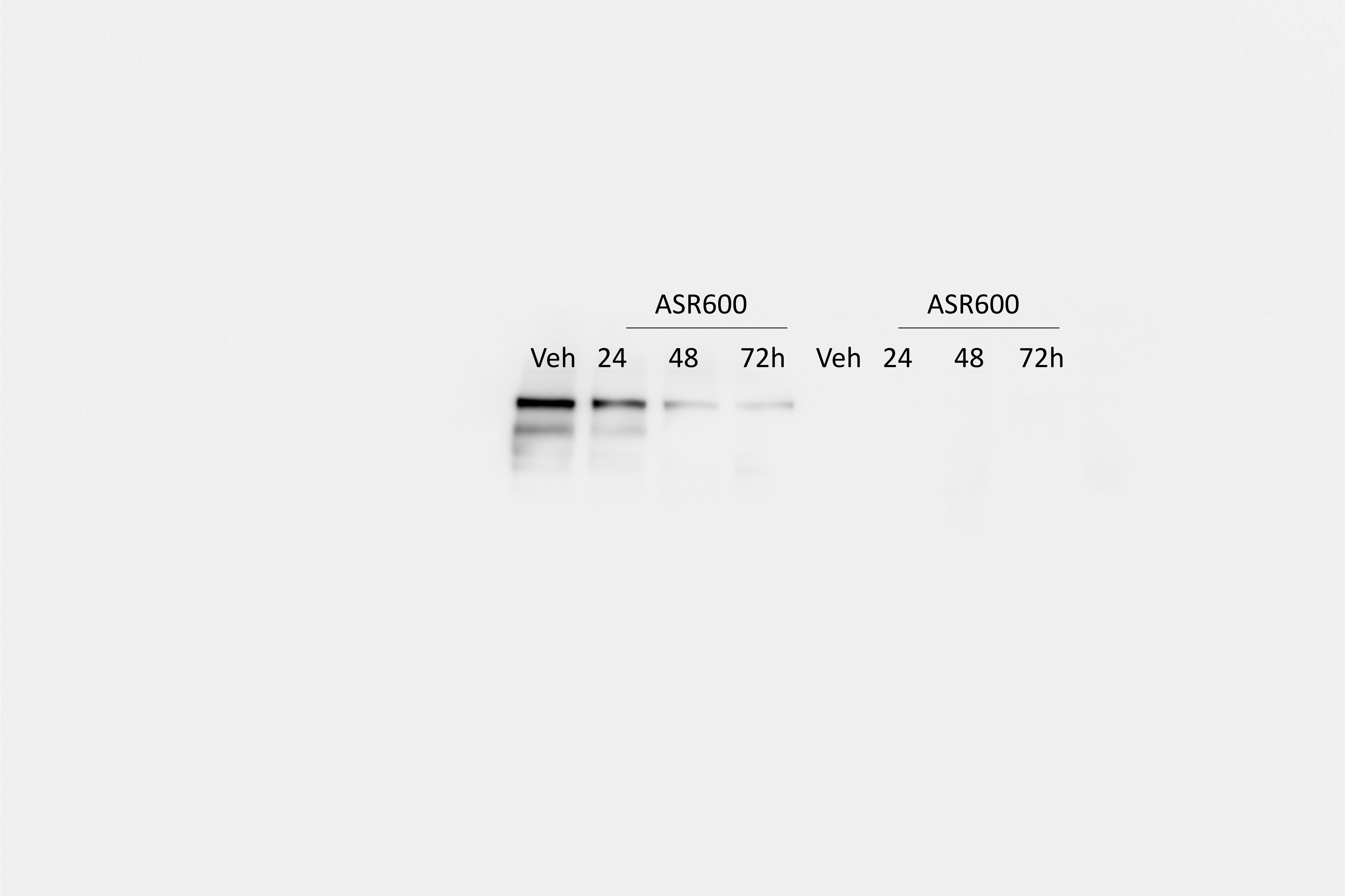

Fig.4d
Actin

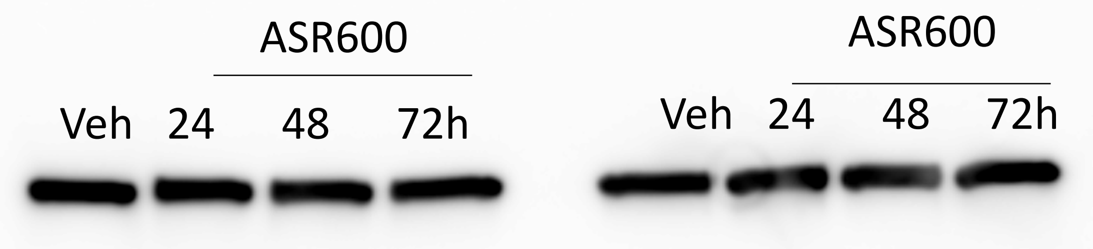

Fig.4e
AR-V7

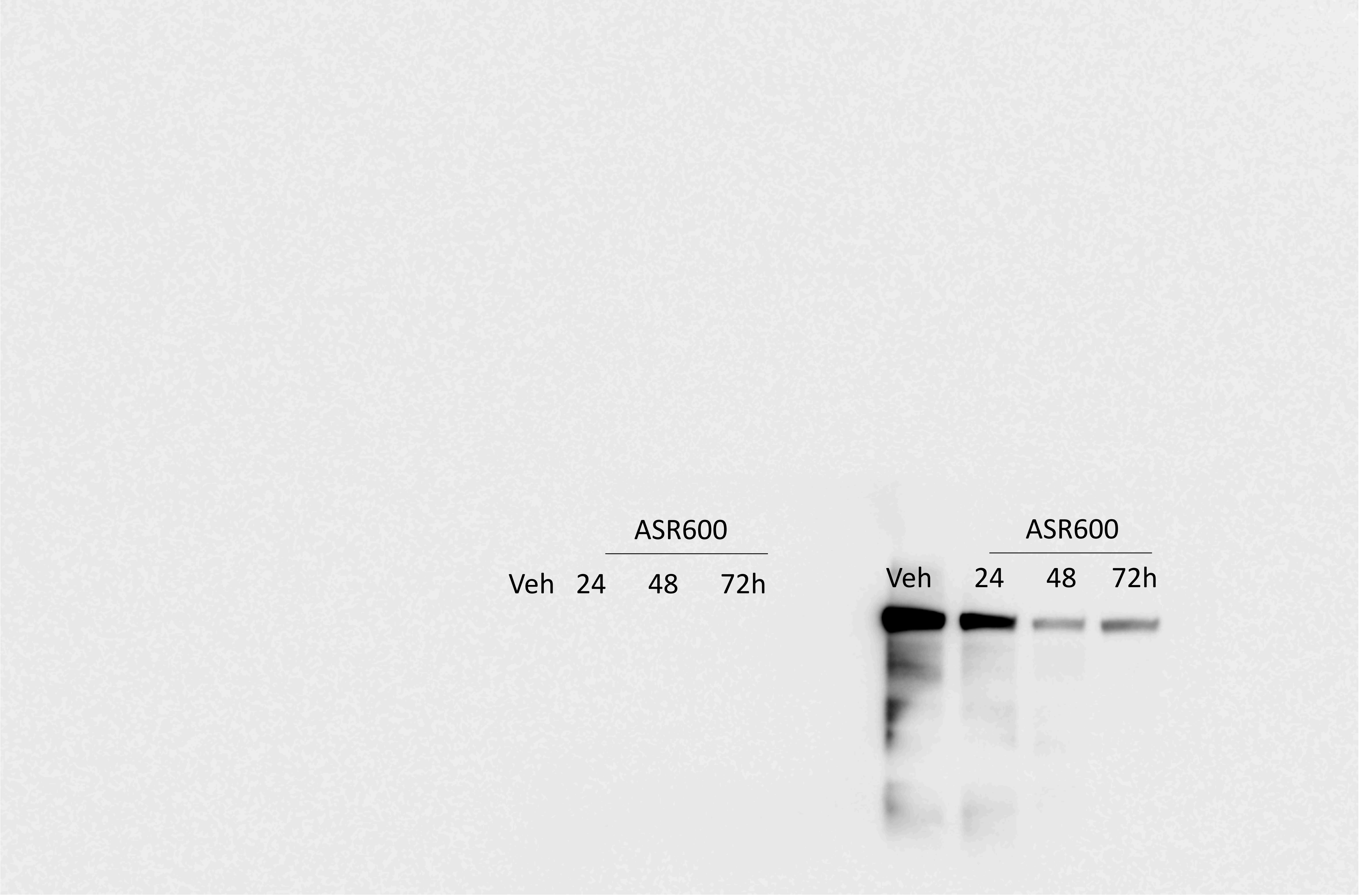

Fig.4e
Actin

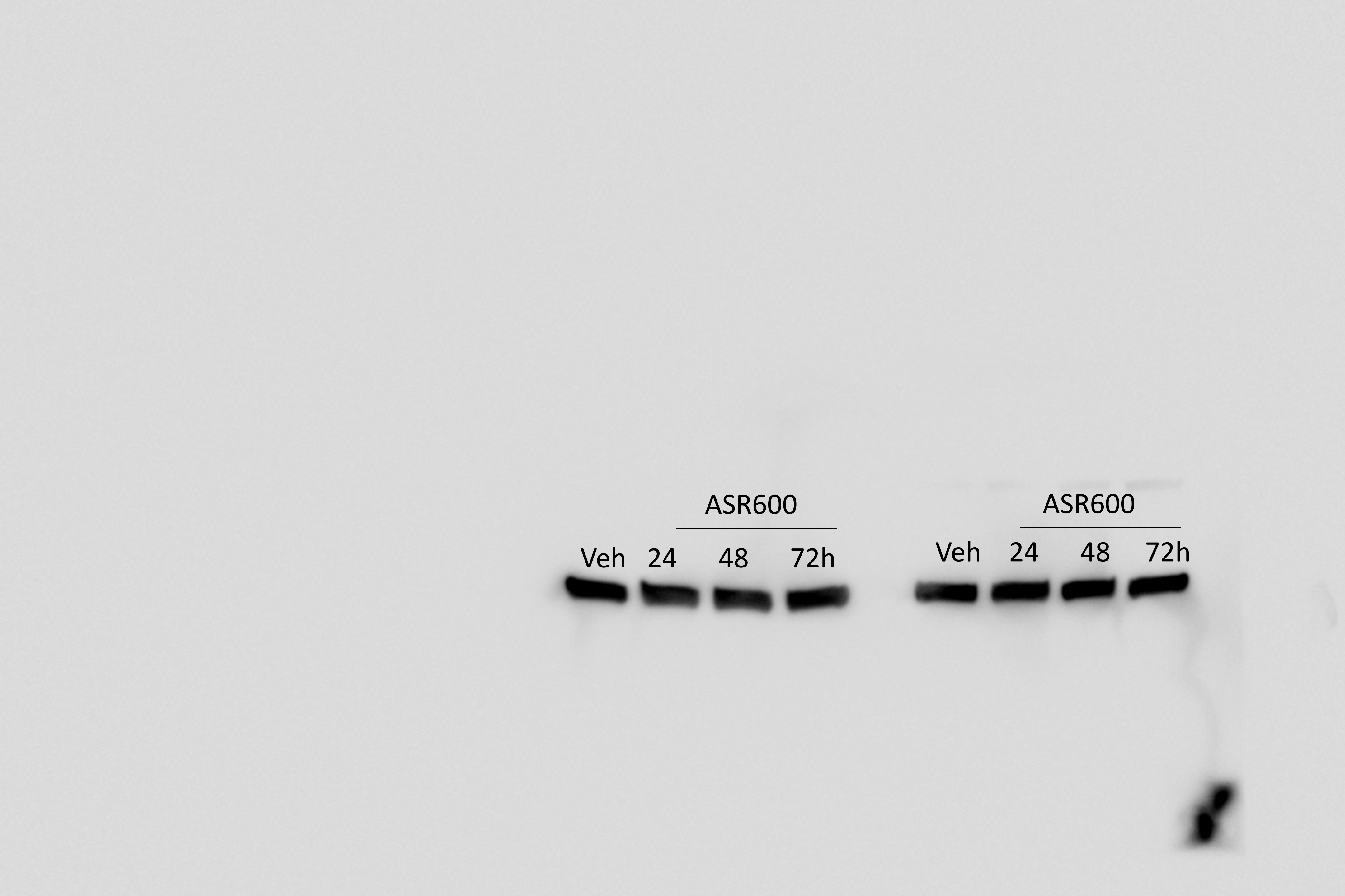

Fig.5a
ASR600

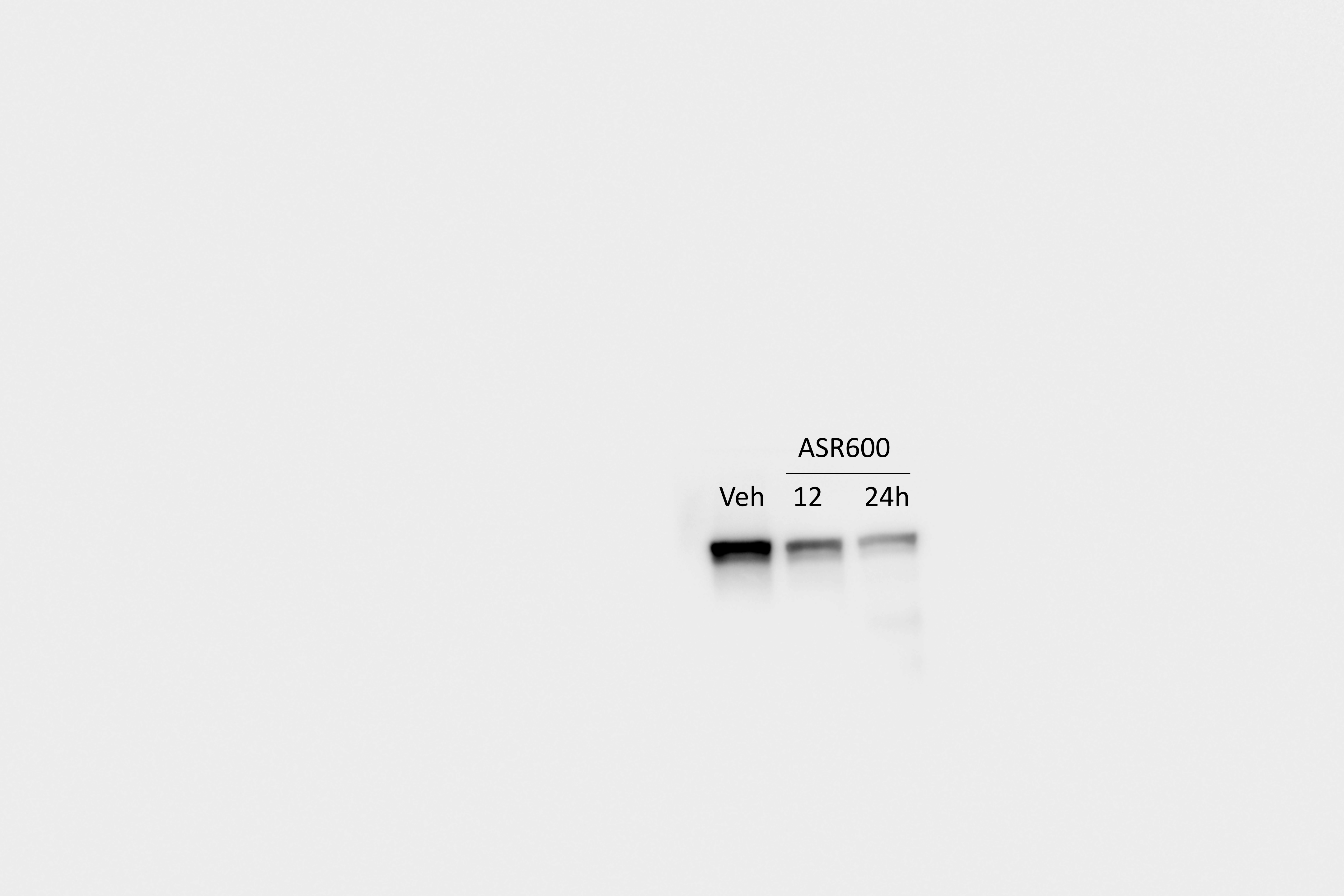

Fig.5a
ASR600 Actin

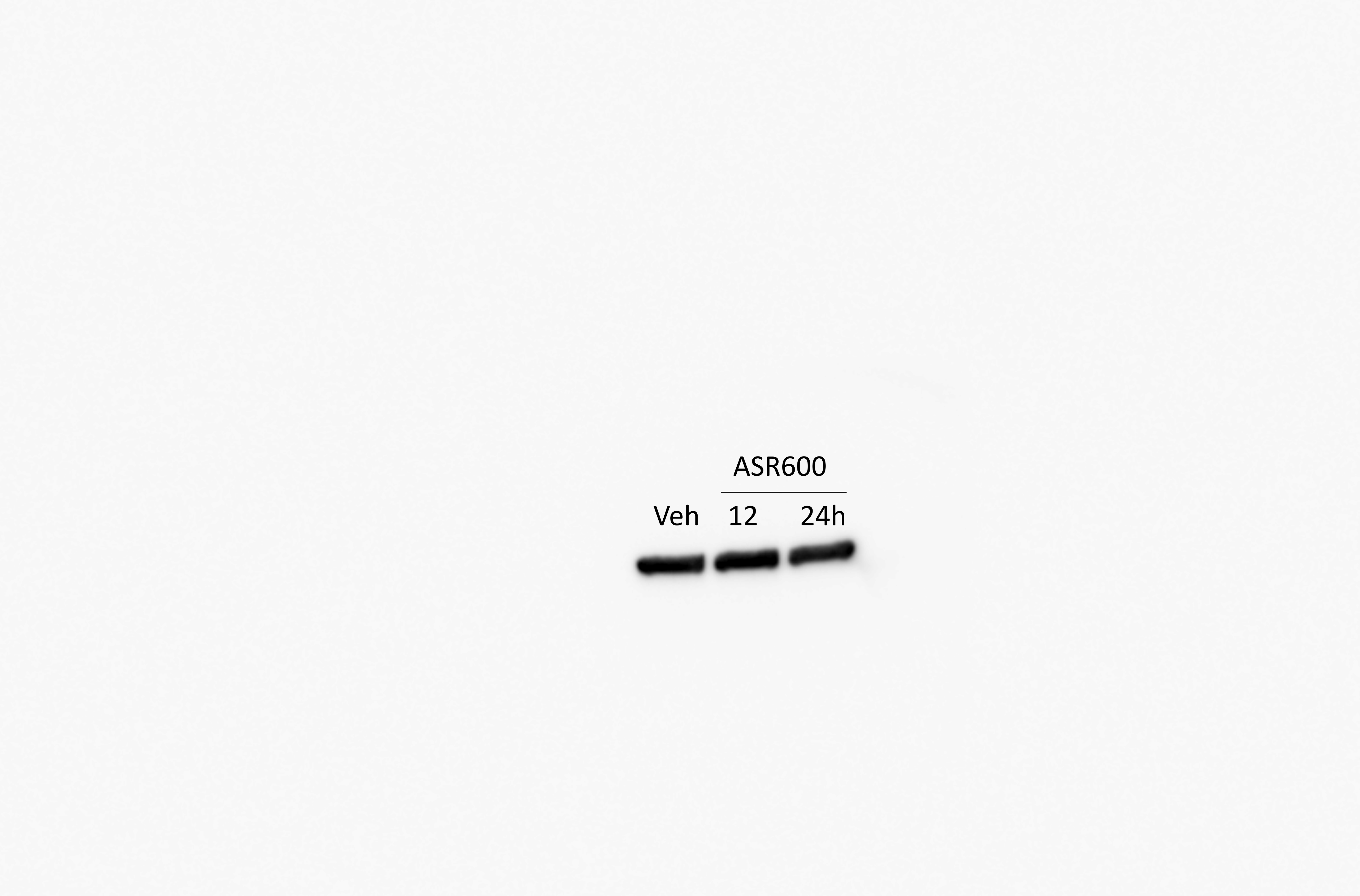

Fig.5b
CHX AR

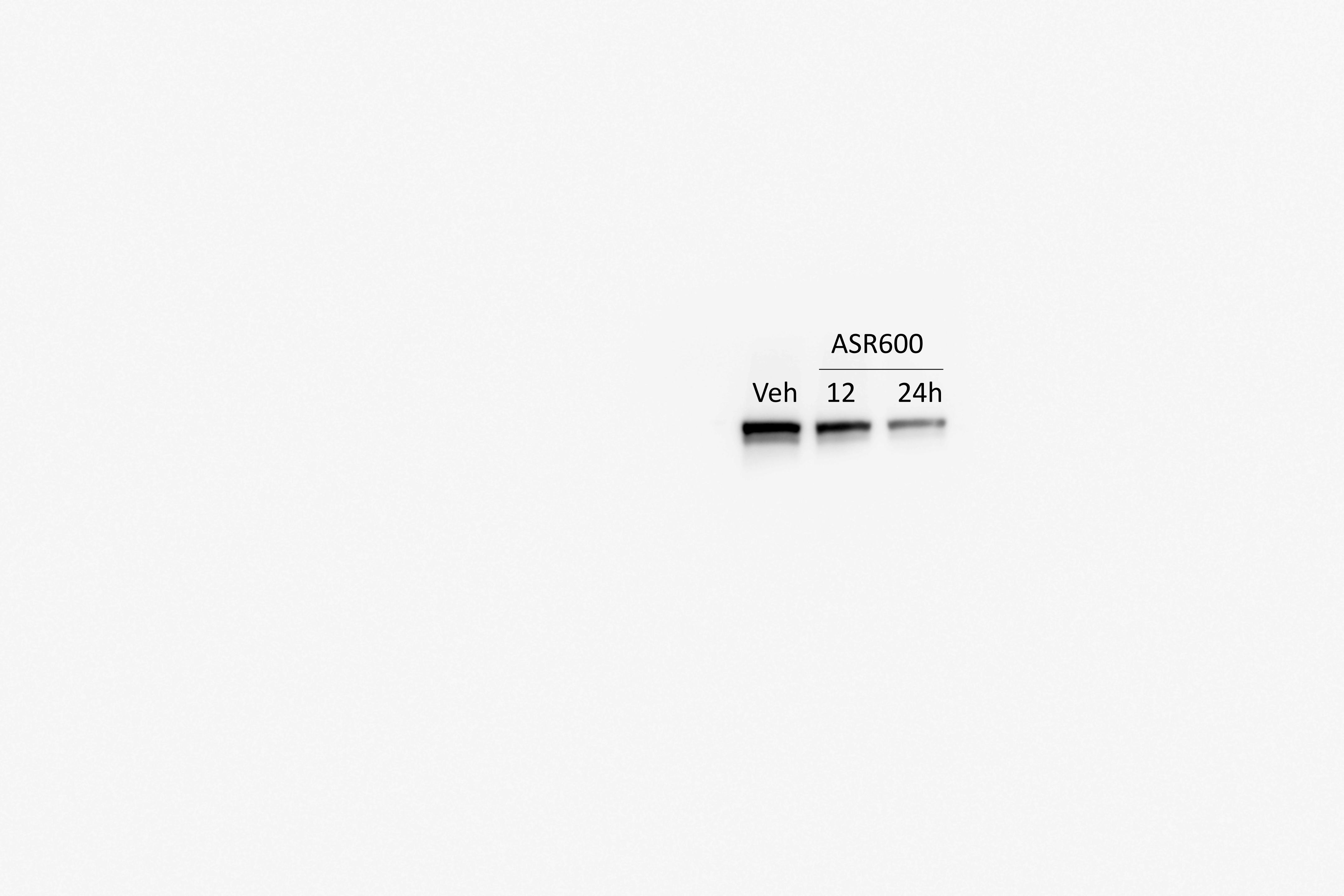

Fig.5b
CHX Act

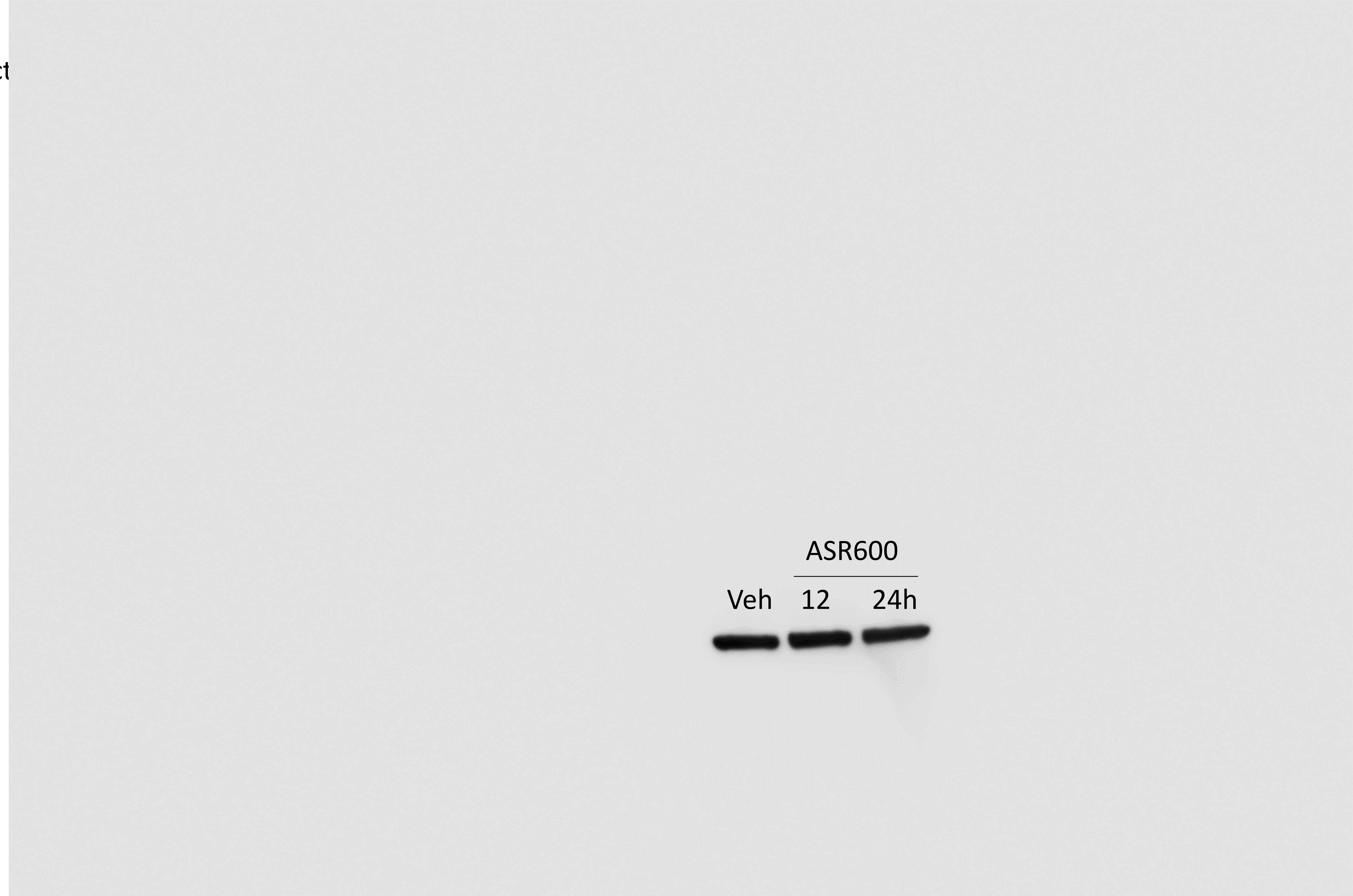

Fig.5c
CHX+ASR600
AR

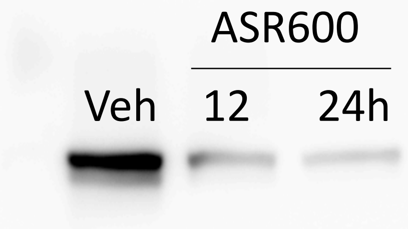

Fig.5c
CHX+ASR600
Actin

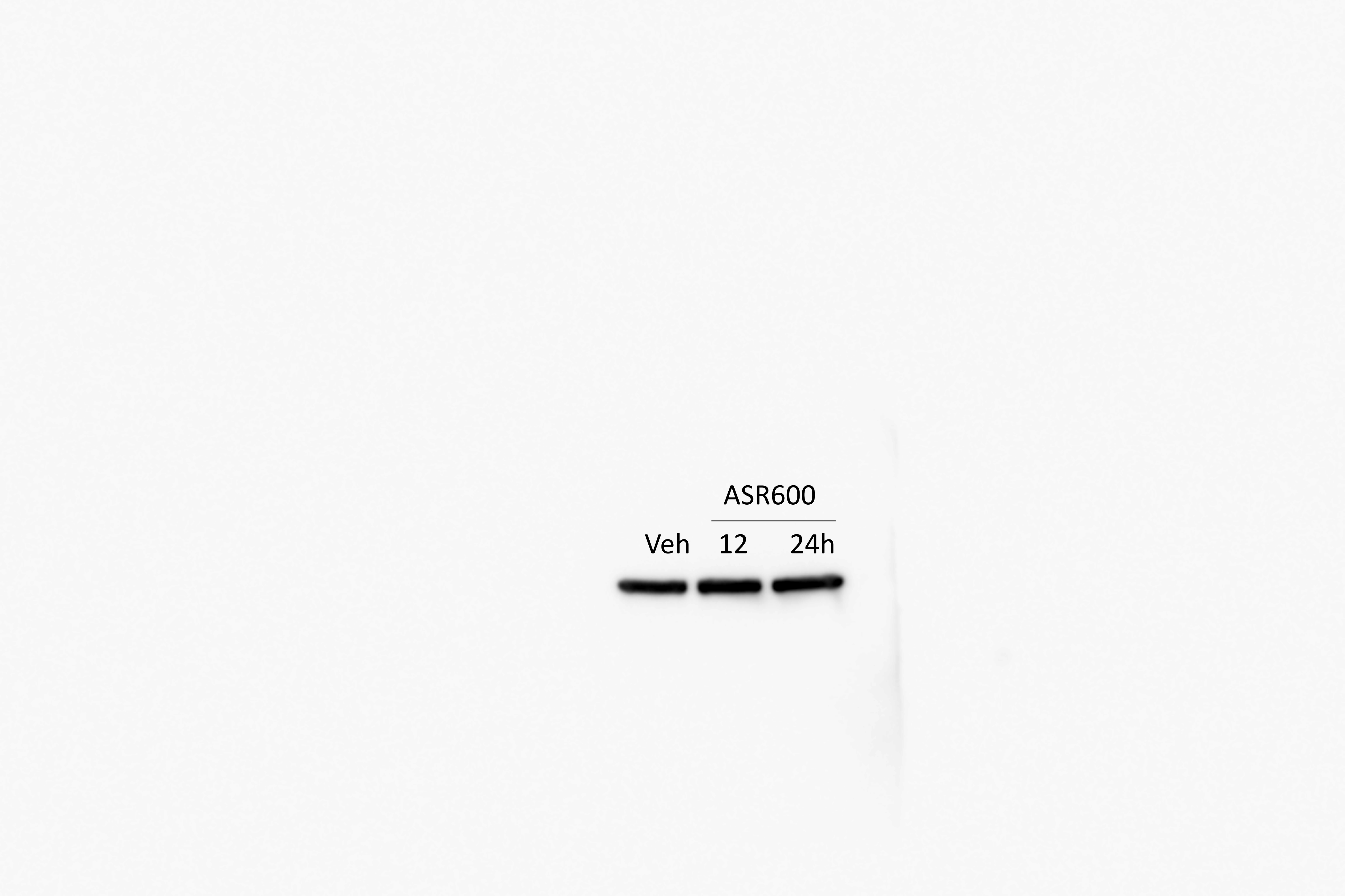

Fig.5d
MG132 AR

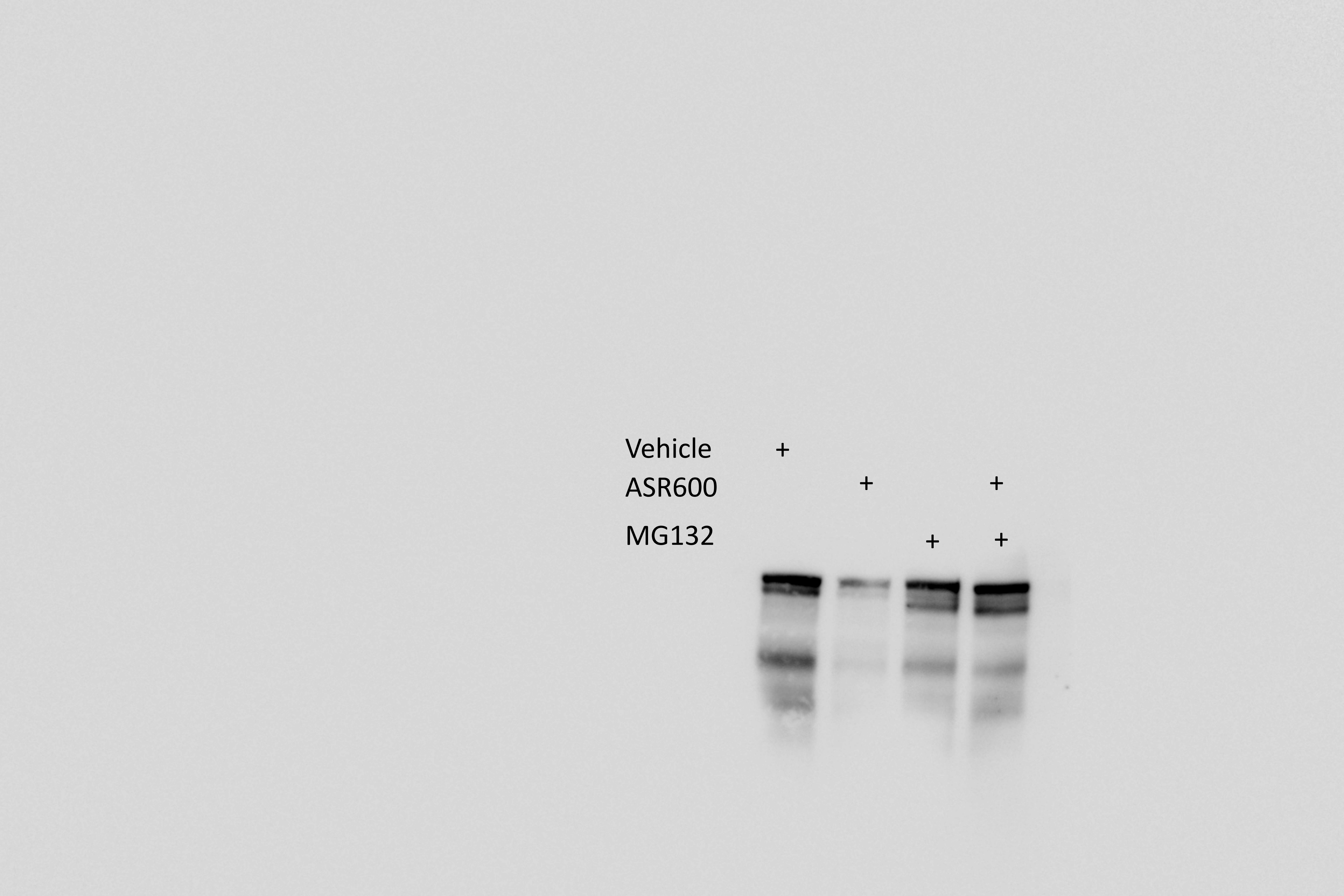

Fig.5d
MG132_
Actin

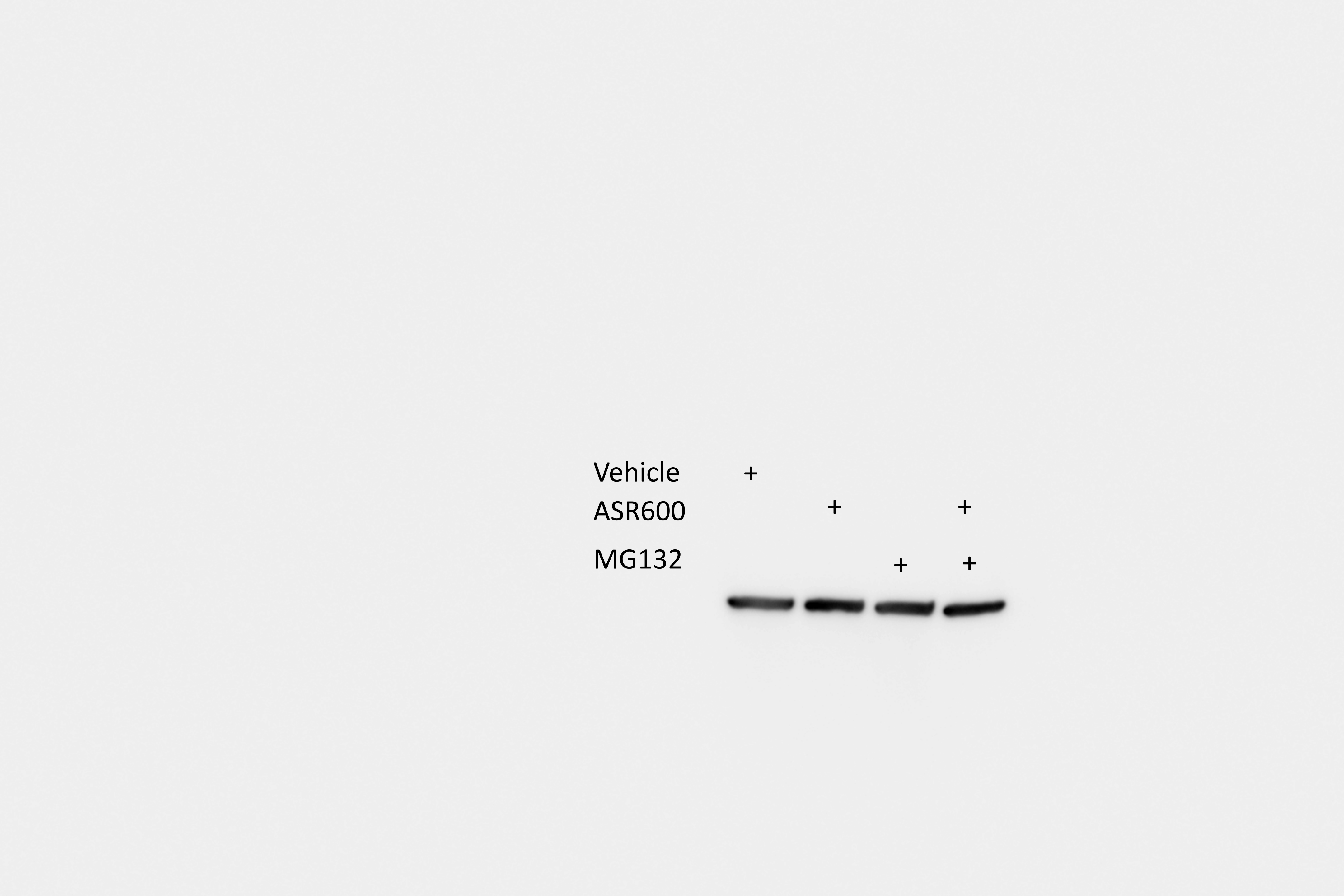

Fig.5e
CQ

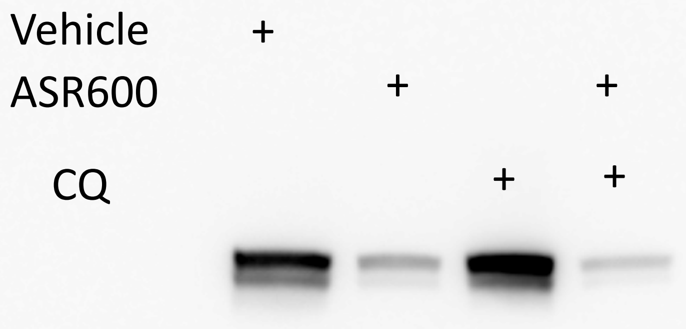

Fig.5e
CQ_Actin

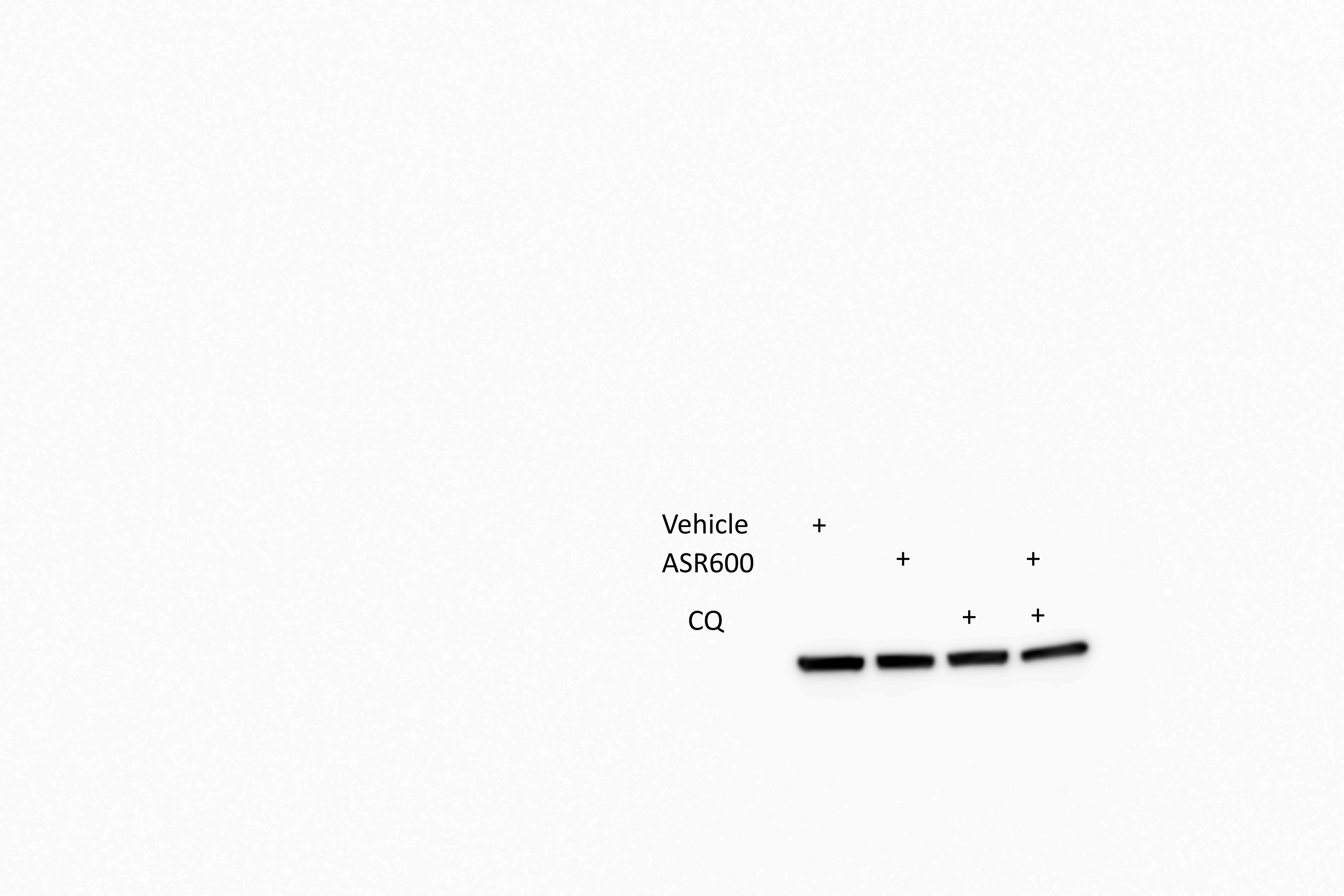

Fig.5f
C4-2B
ubi

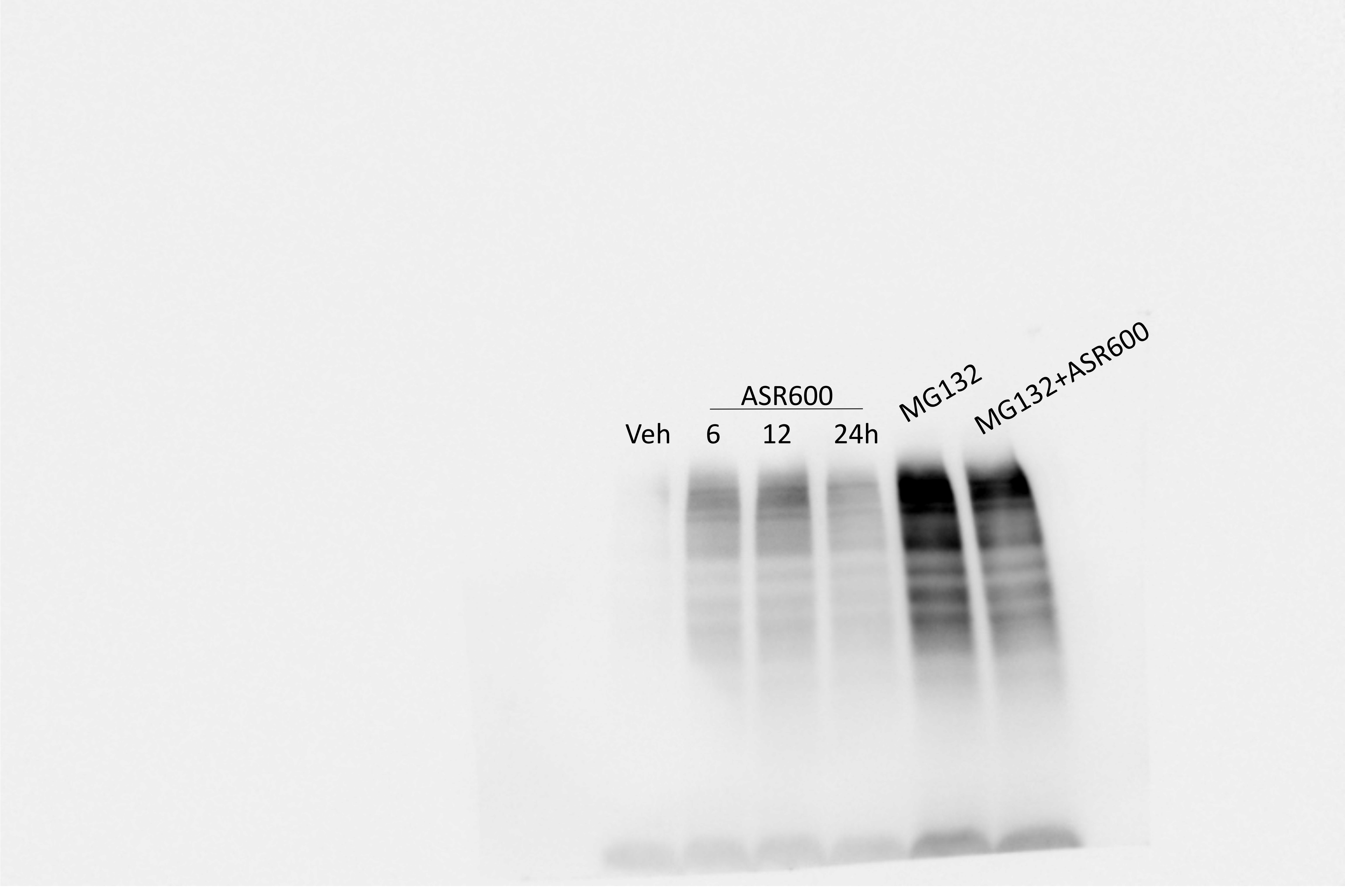

Fig.5f
C4-2B
Actin

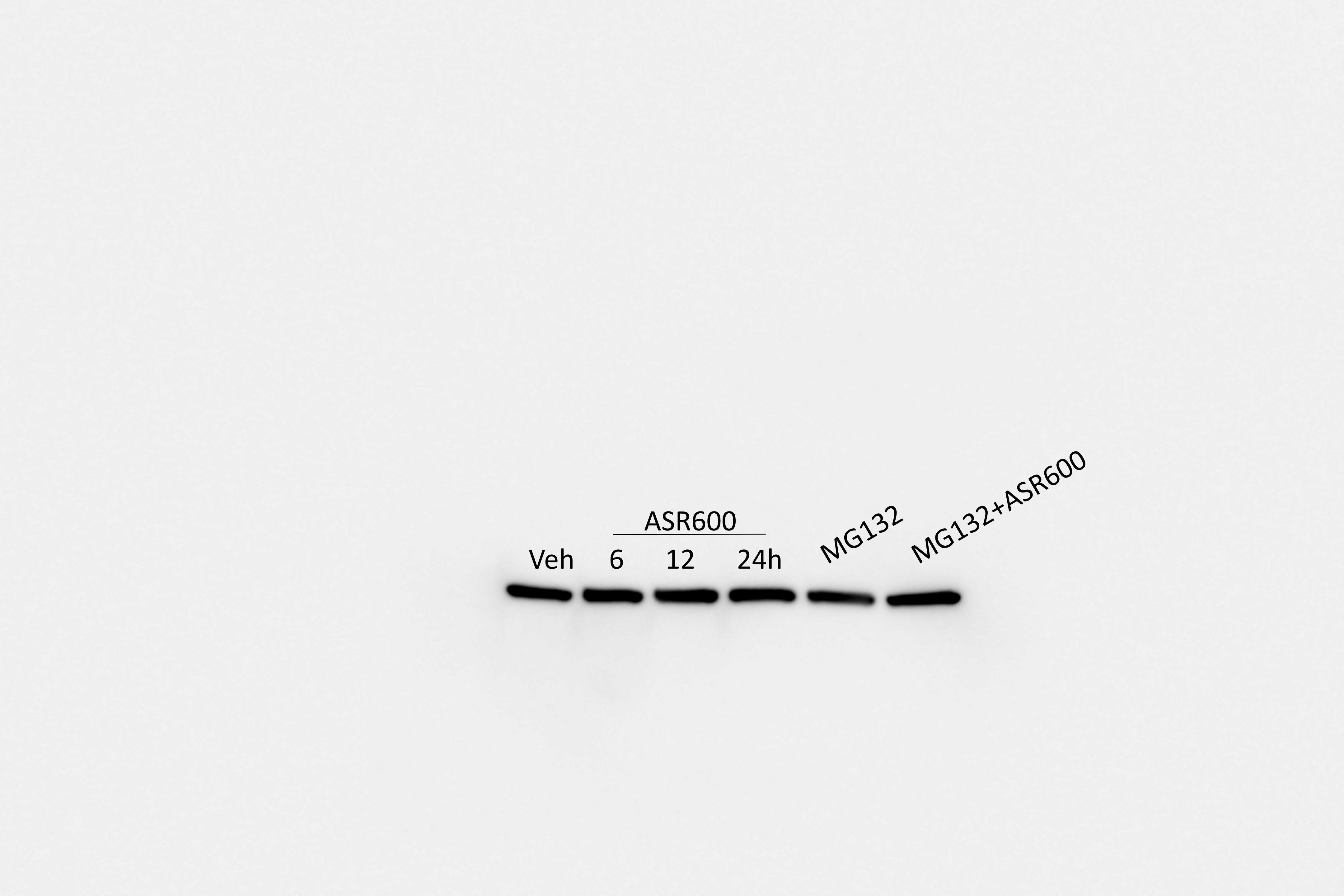

Fig.5g
22RV1

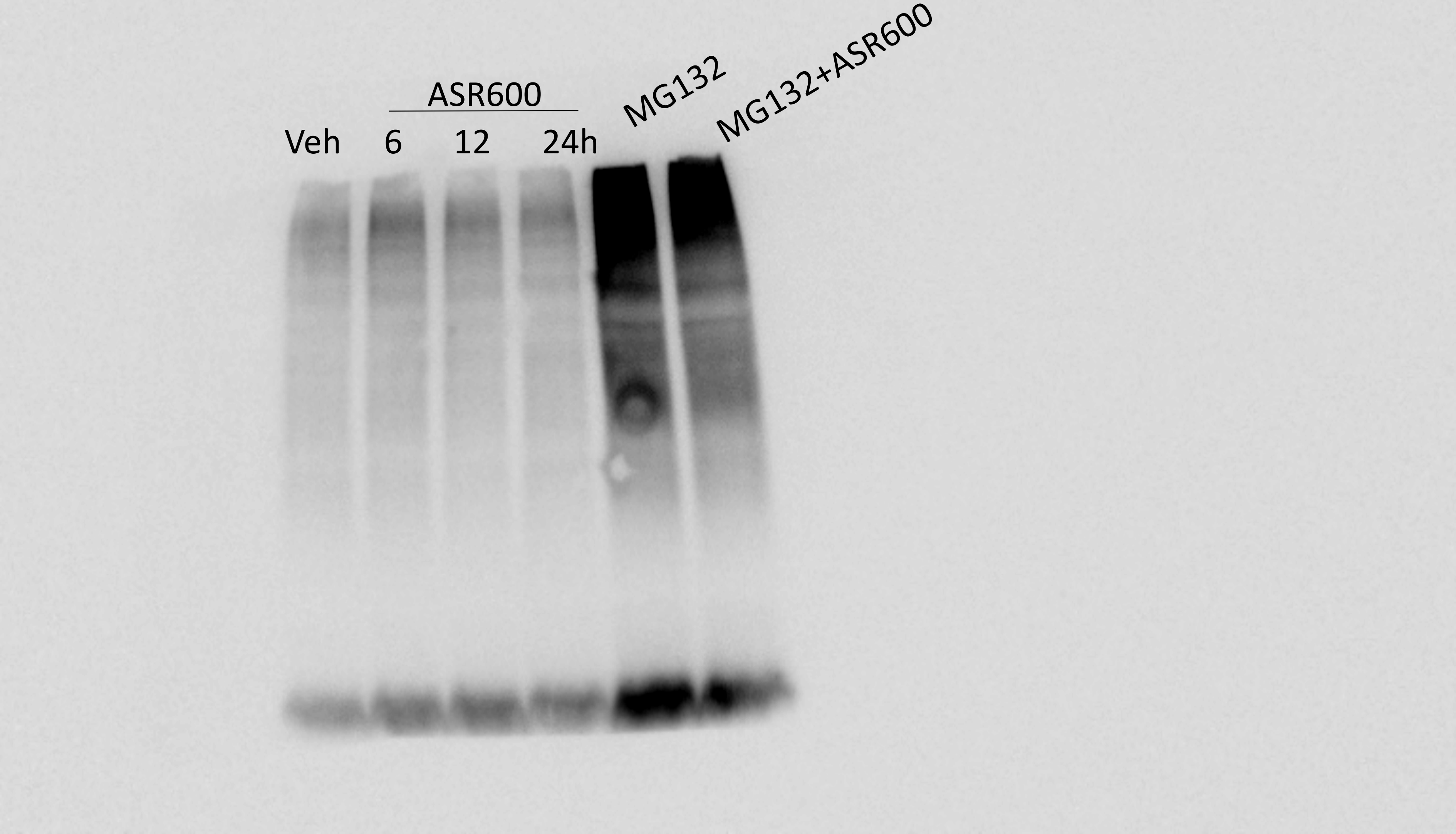

Fig.5g
22RV1
Actin

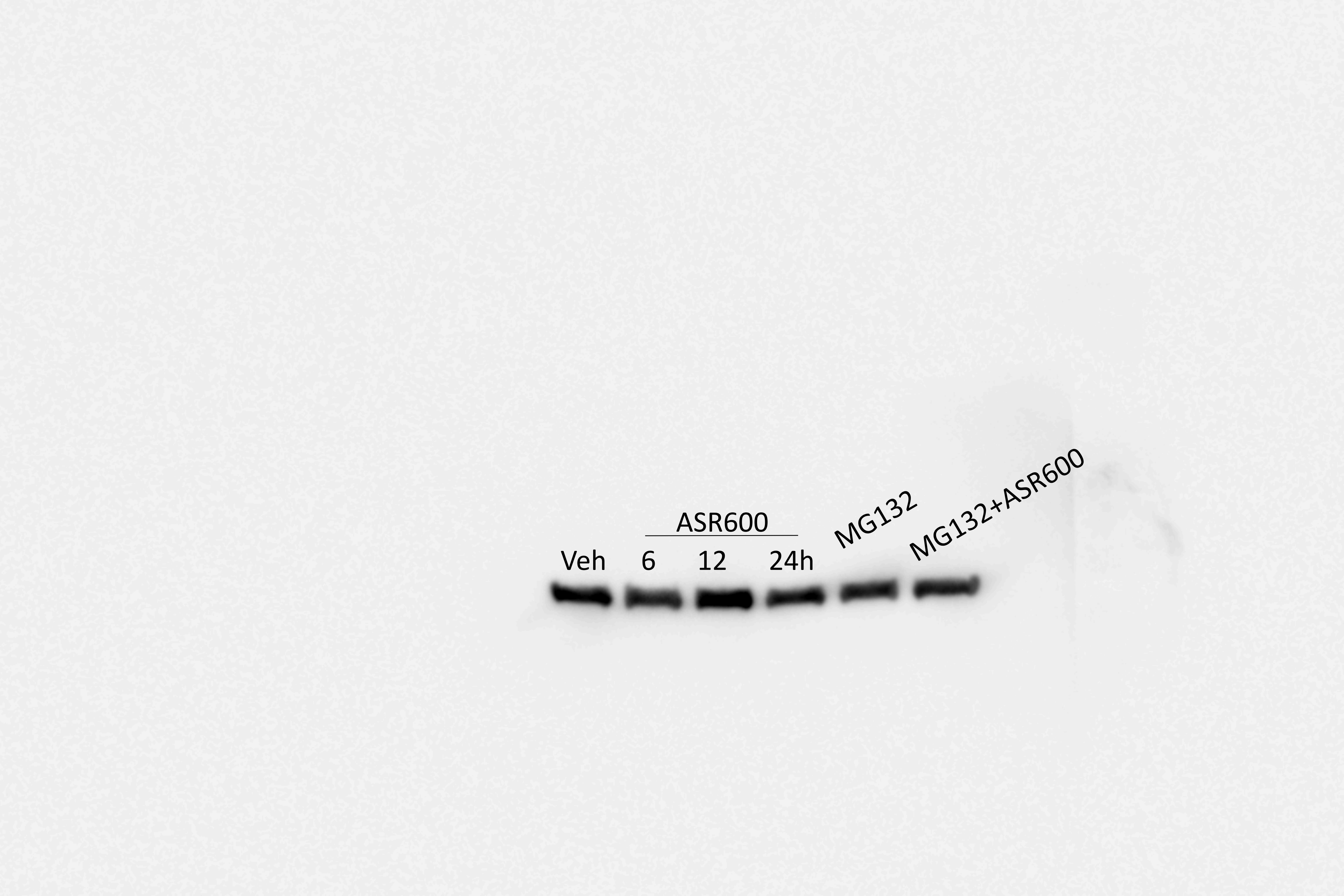

Fig. 5h
C4-
2B+IP
ubi

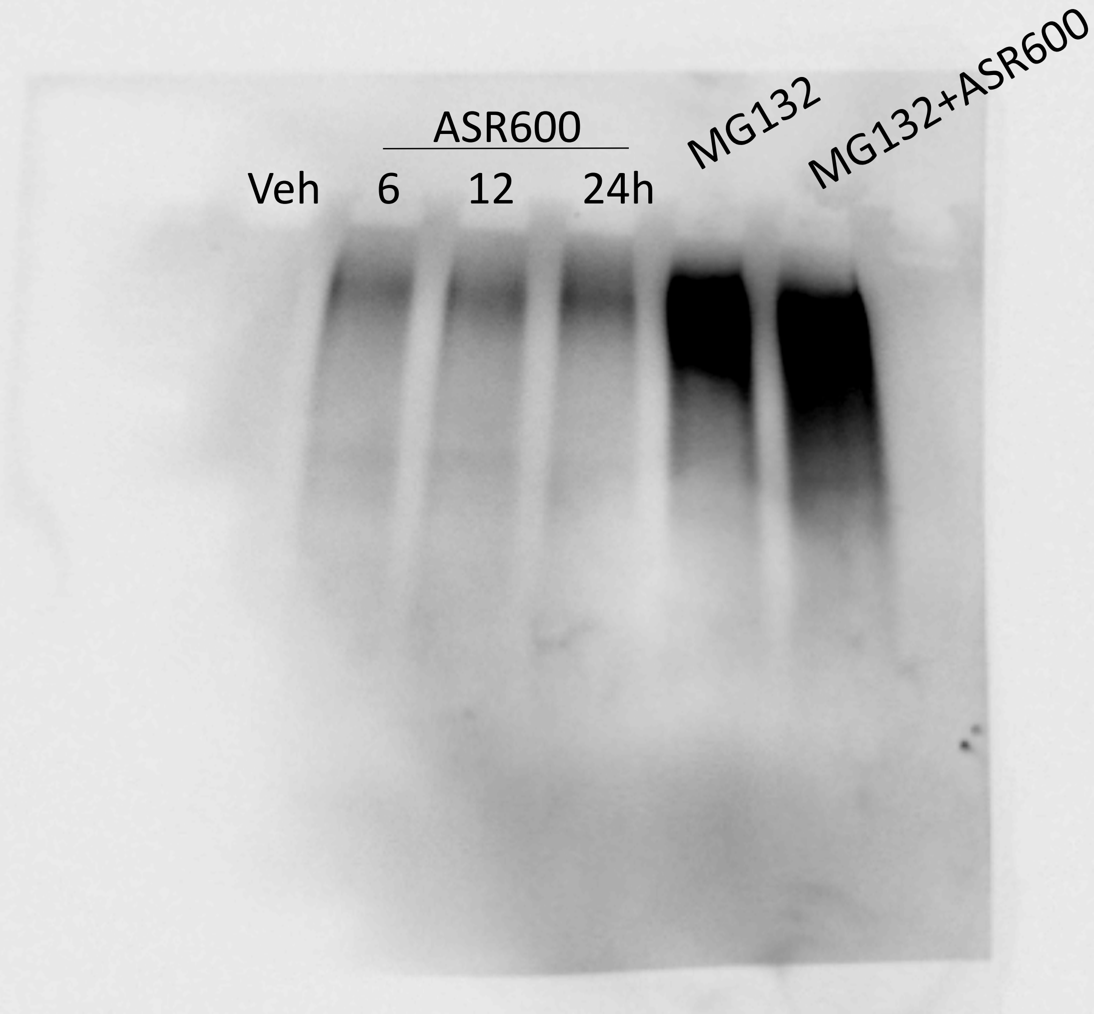

Fig. 5h
C4-2B
Input AR

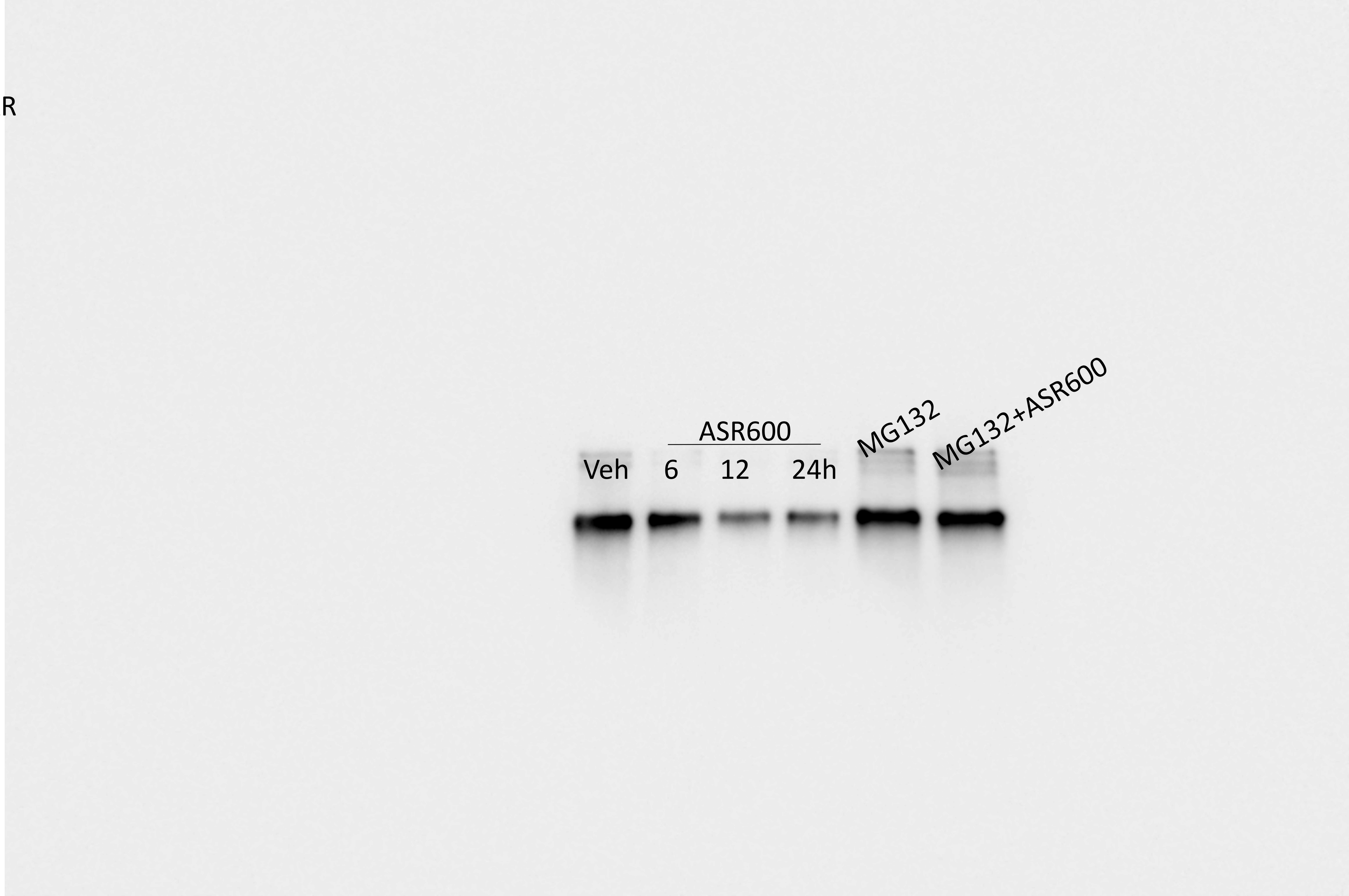

Fig. 5i
22Rv1+IP
Ubi

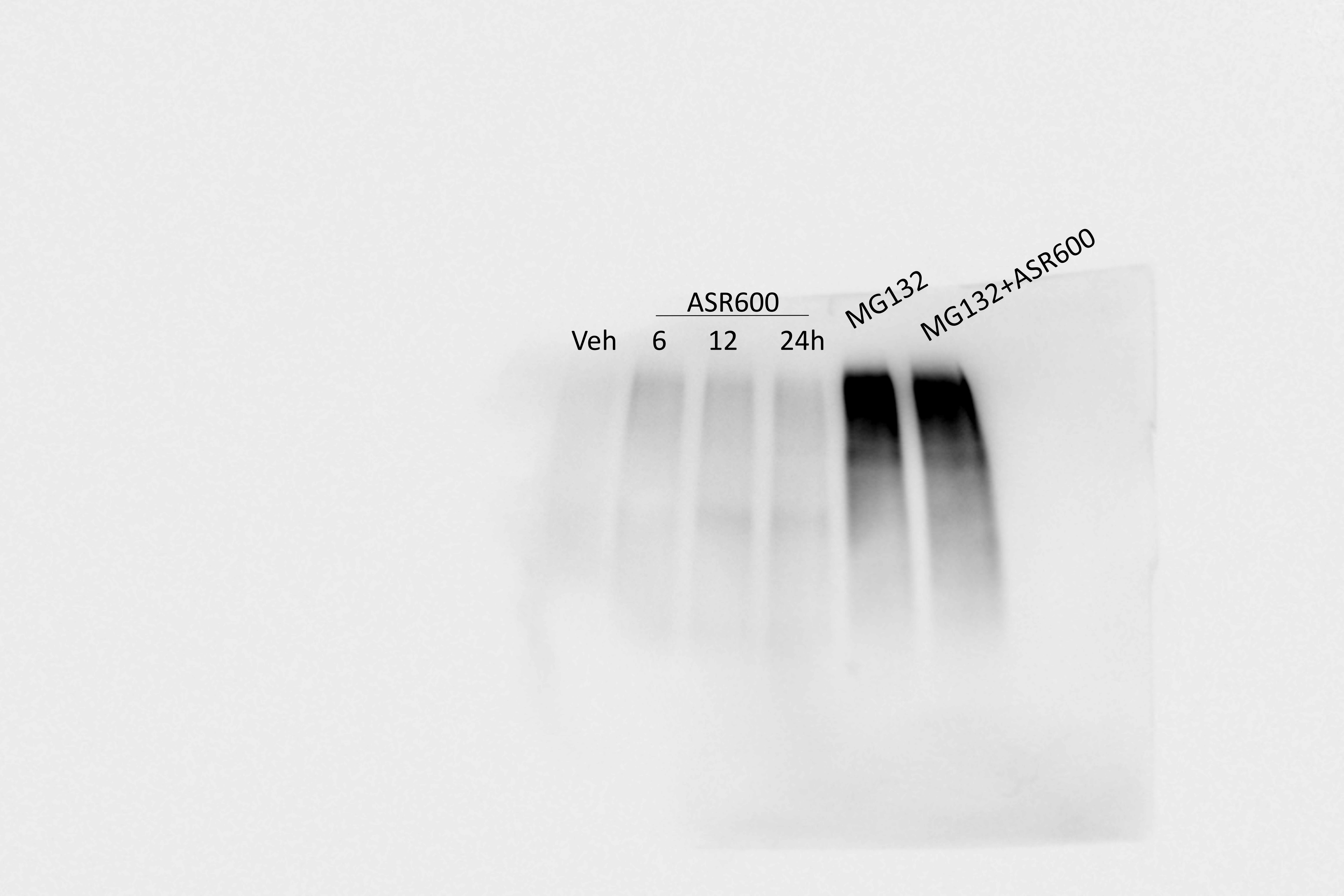

Fig. 5i
Input

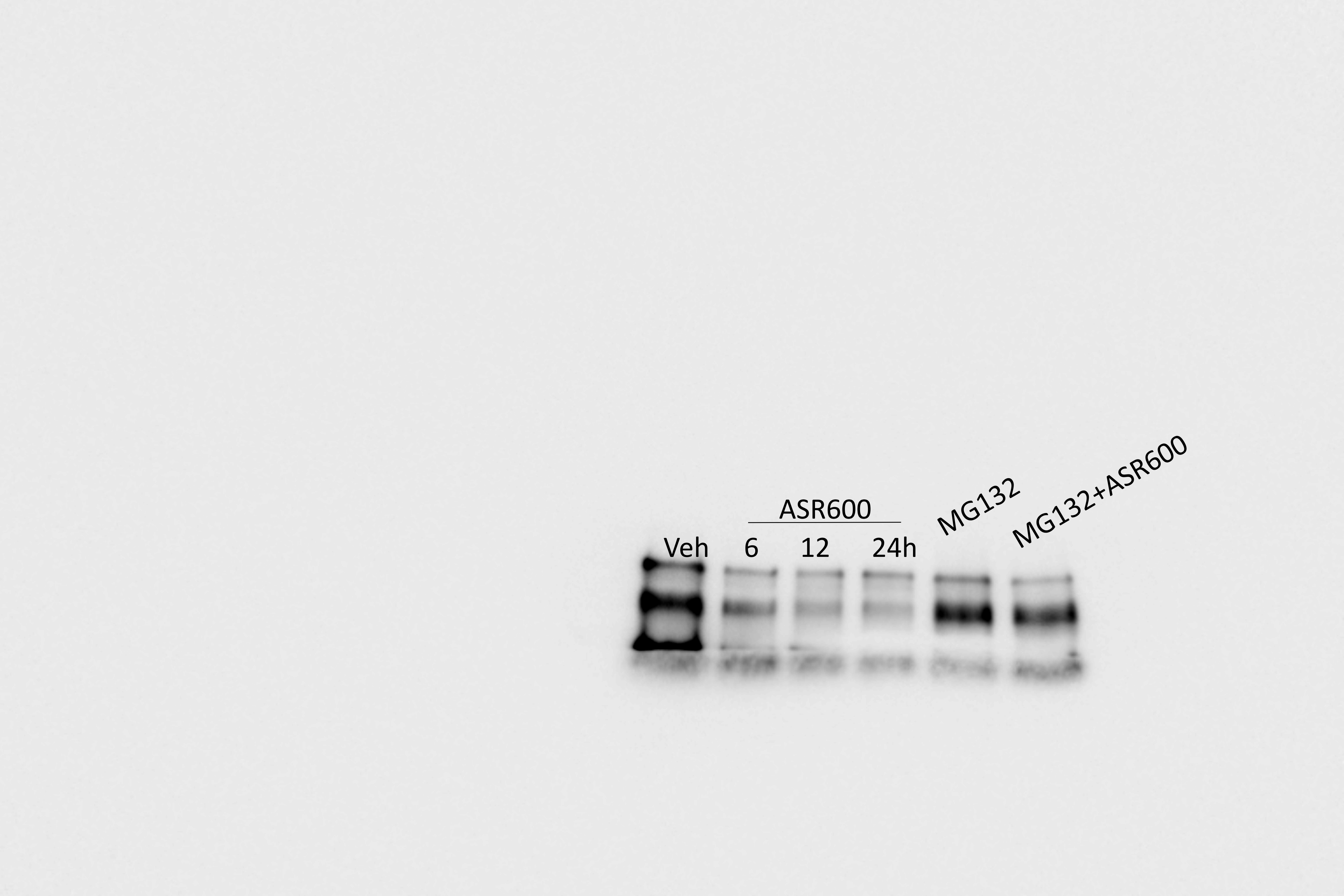

Fig. 5K &J

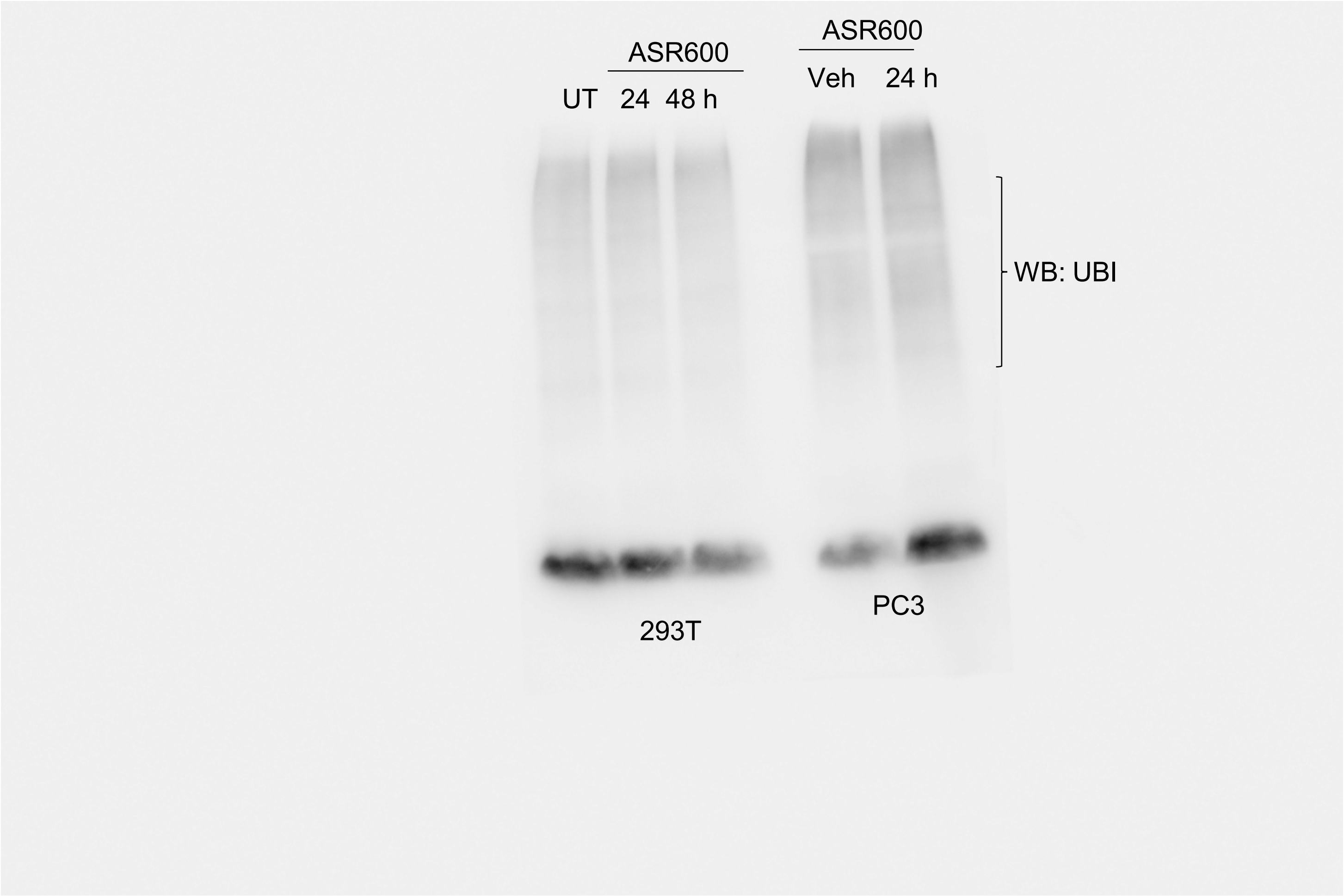

Fig. 5J &K

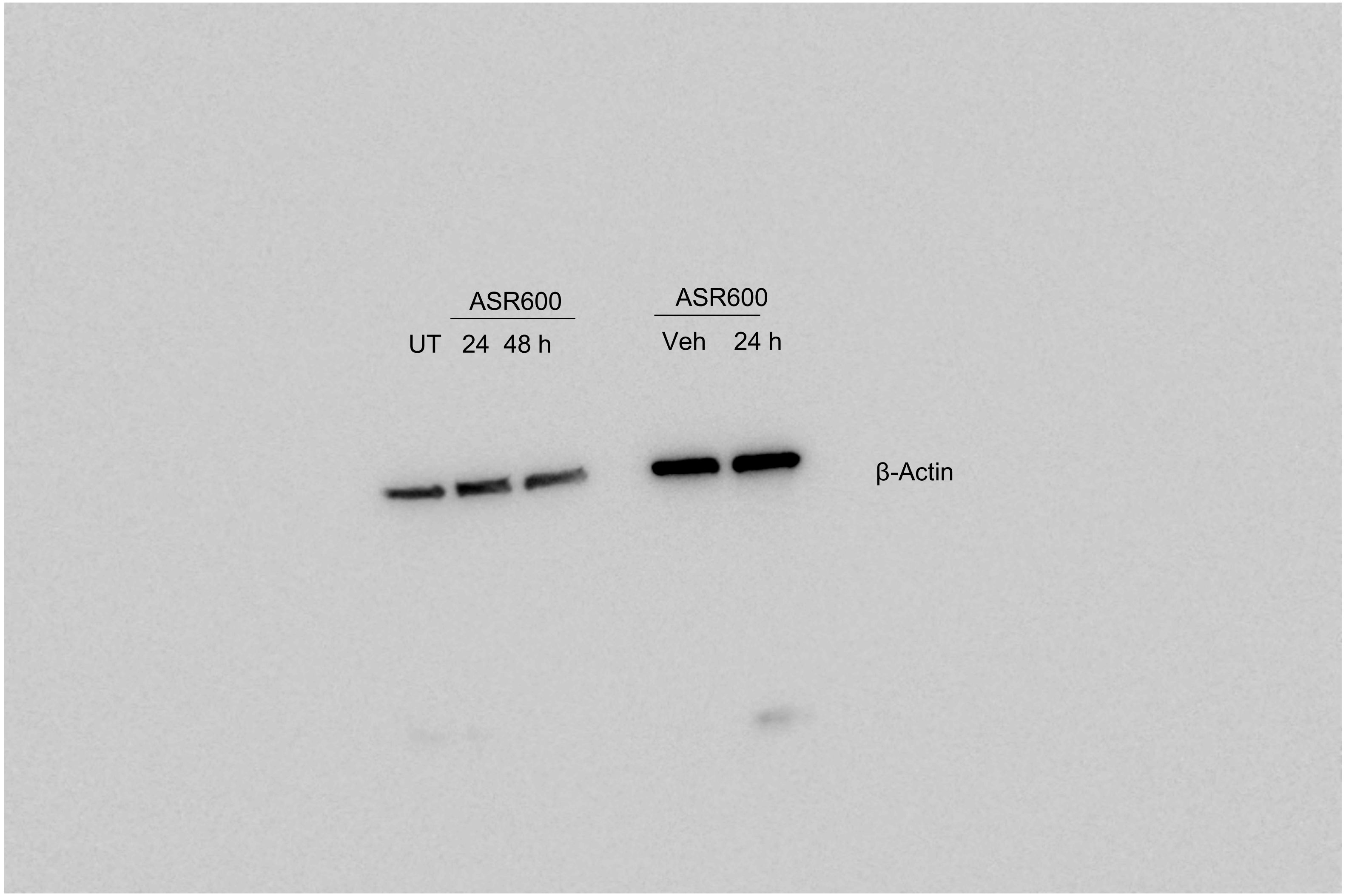

Fig. 5L

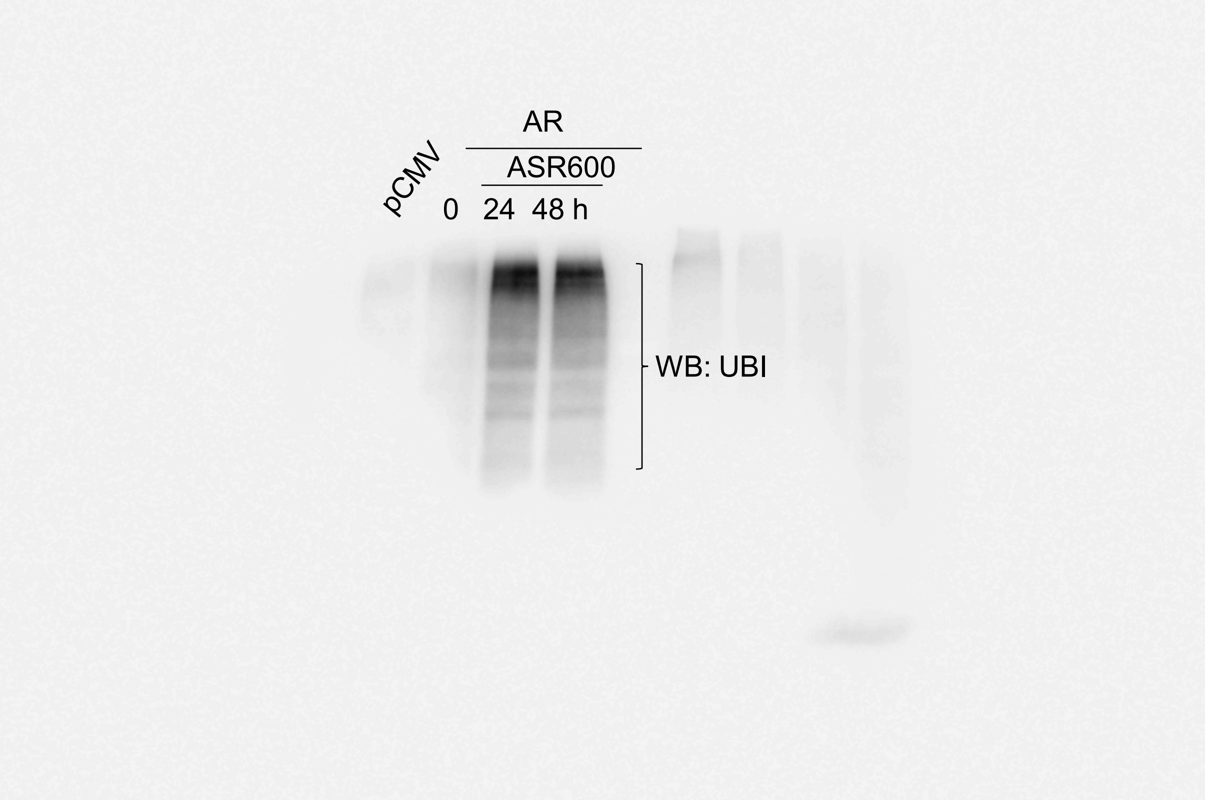
AR
pCMV
ASR600
0 24 48 h
WB: UBI

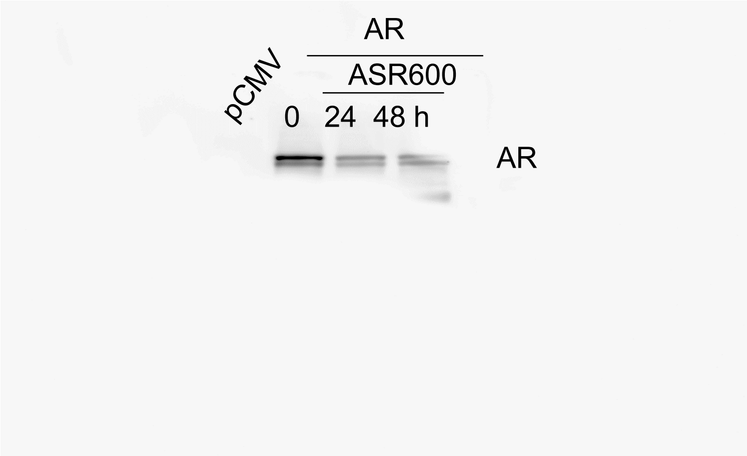
AR
pCMV
ASR600
0 24 48 h
AR

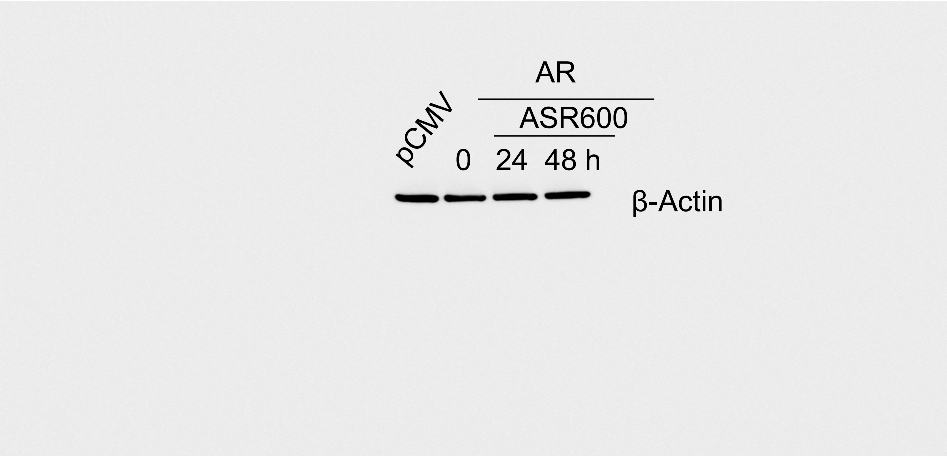
AR
pCMV
ASR600
0 24 48 h
β-Actin

Fig.5n

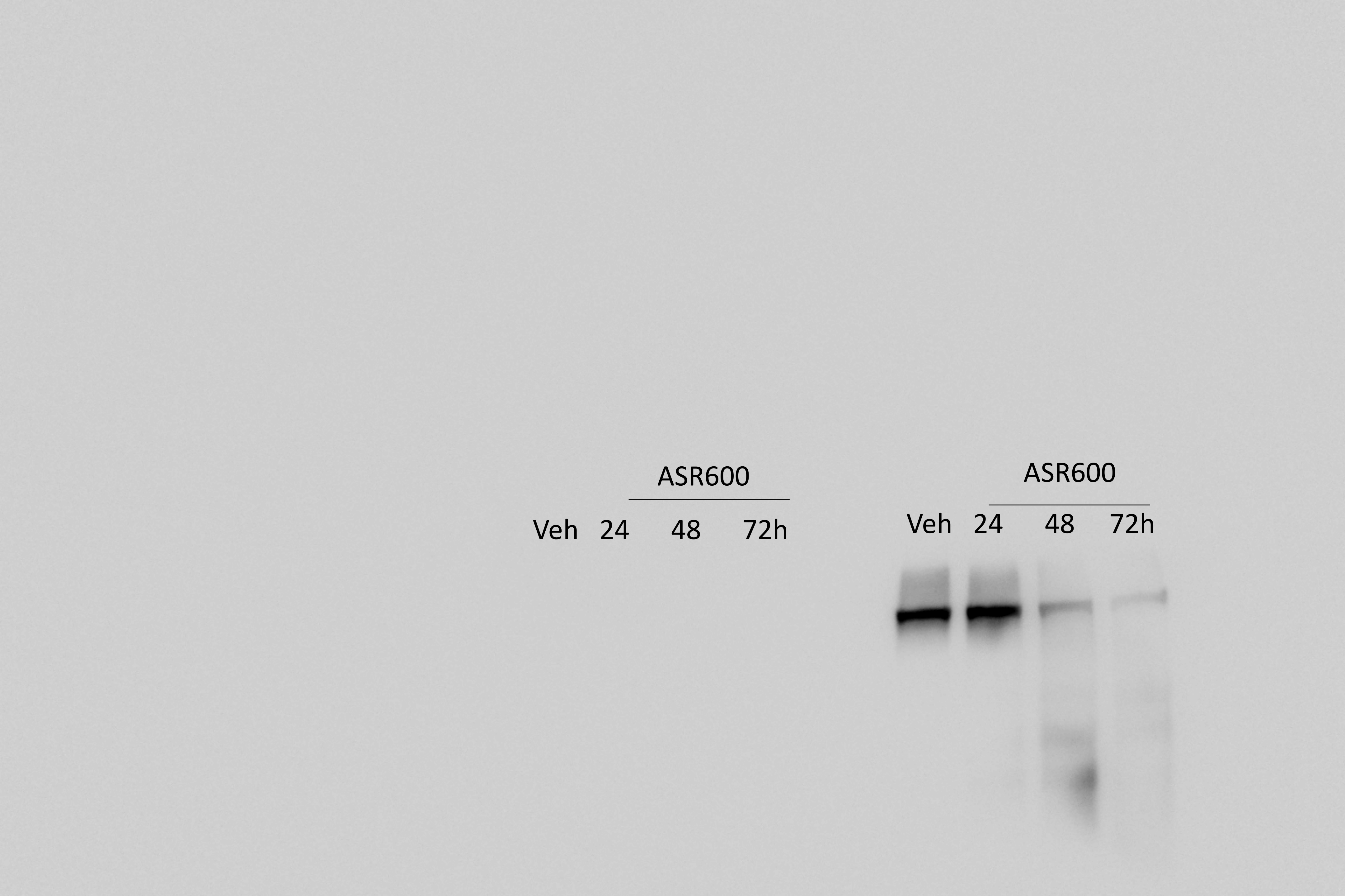

Fig.5n
Actin

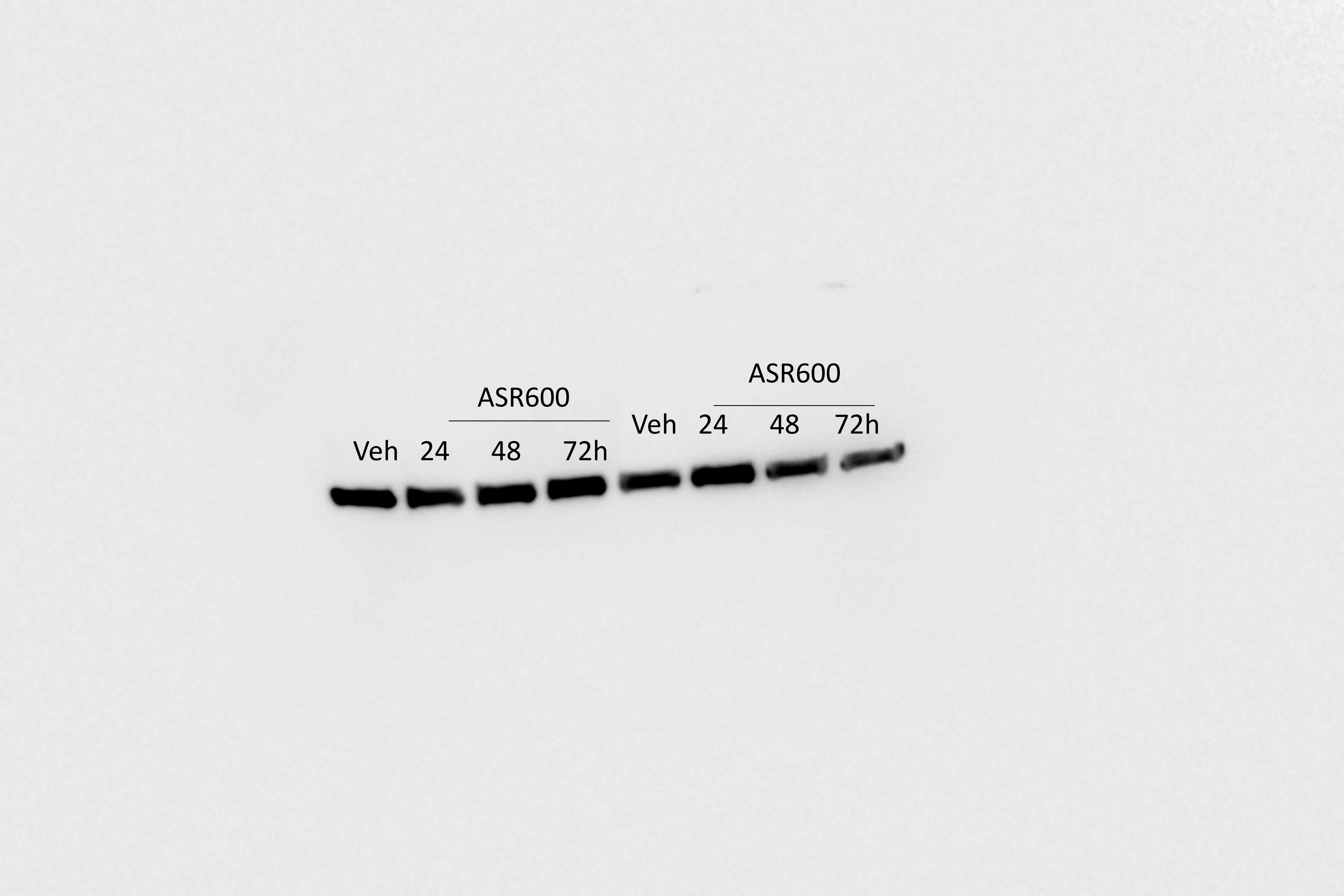

Fig.6a
pAKT

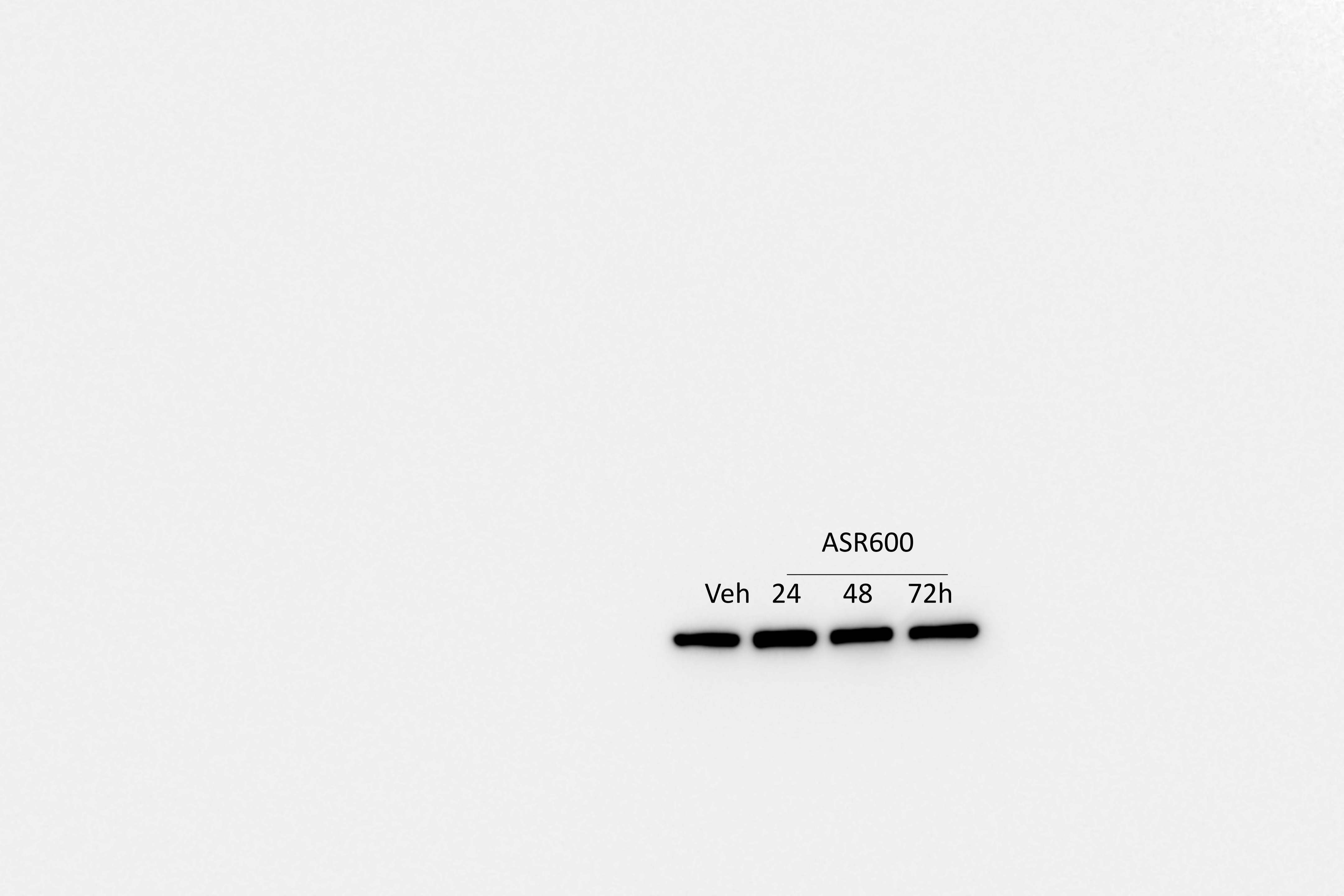

Fig.6a
AKT

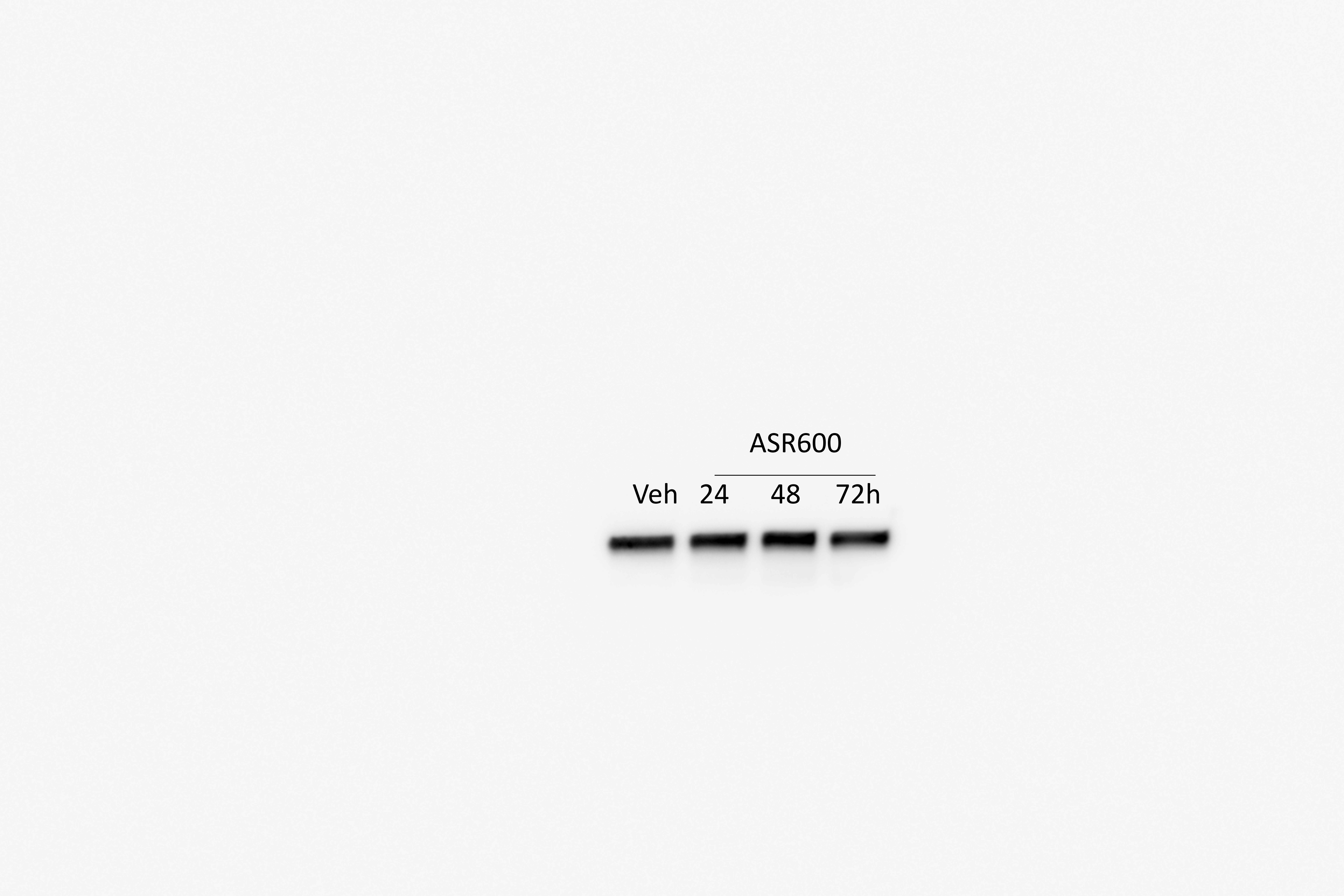

Fig.6a
Actin

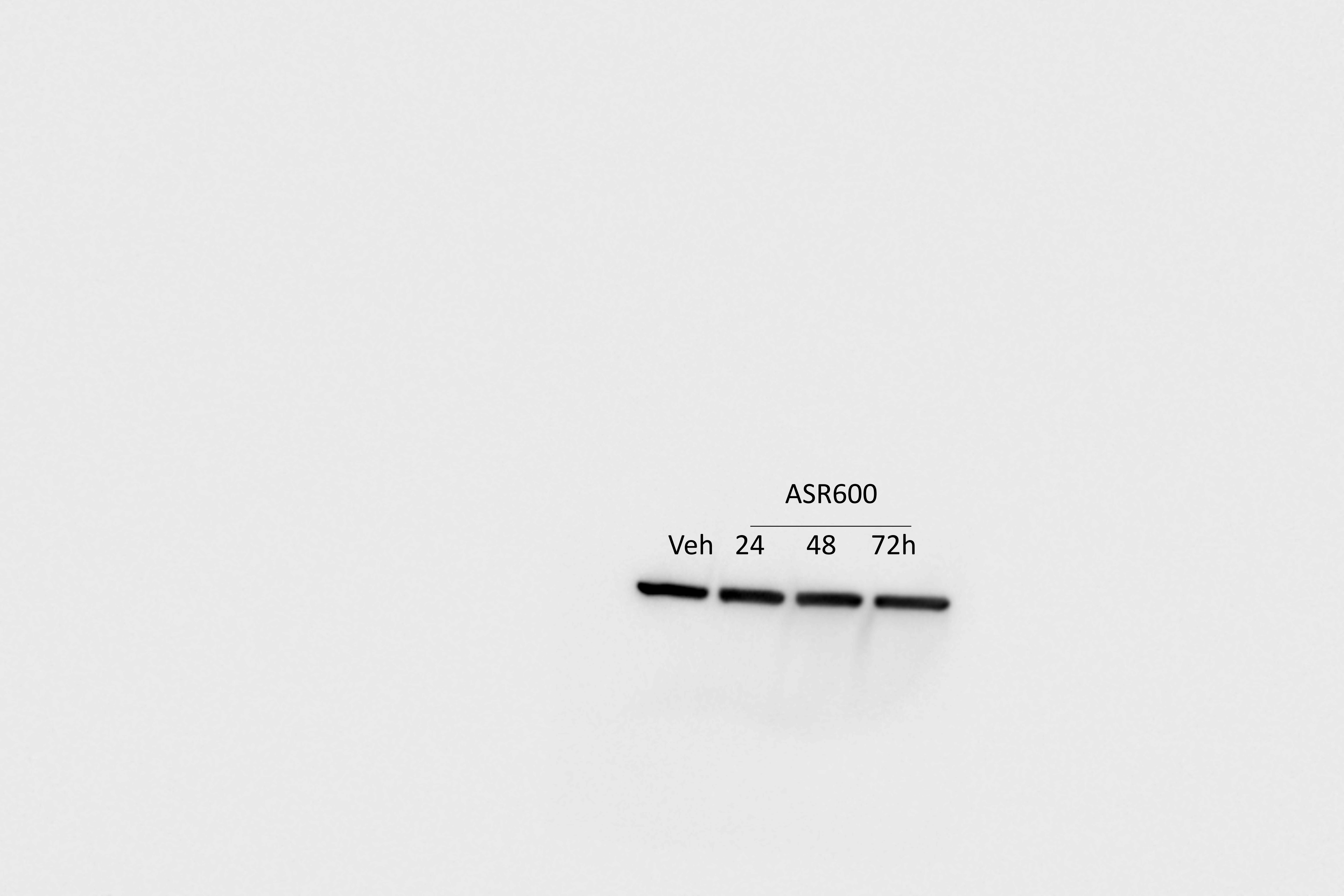

Fig.6b
pmTOR

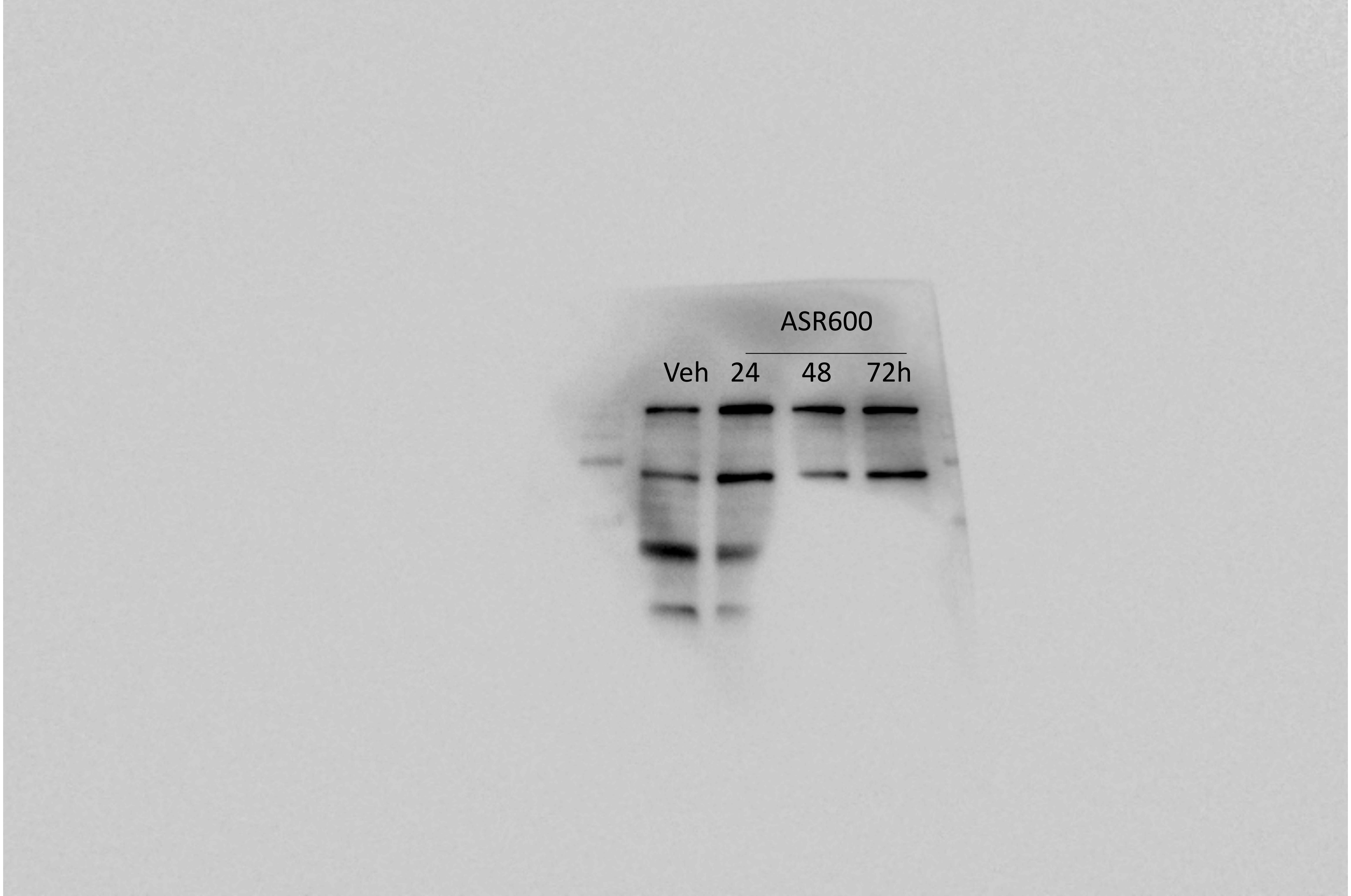

Fig.6b
pMTOR
Actin

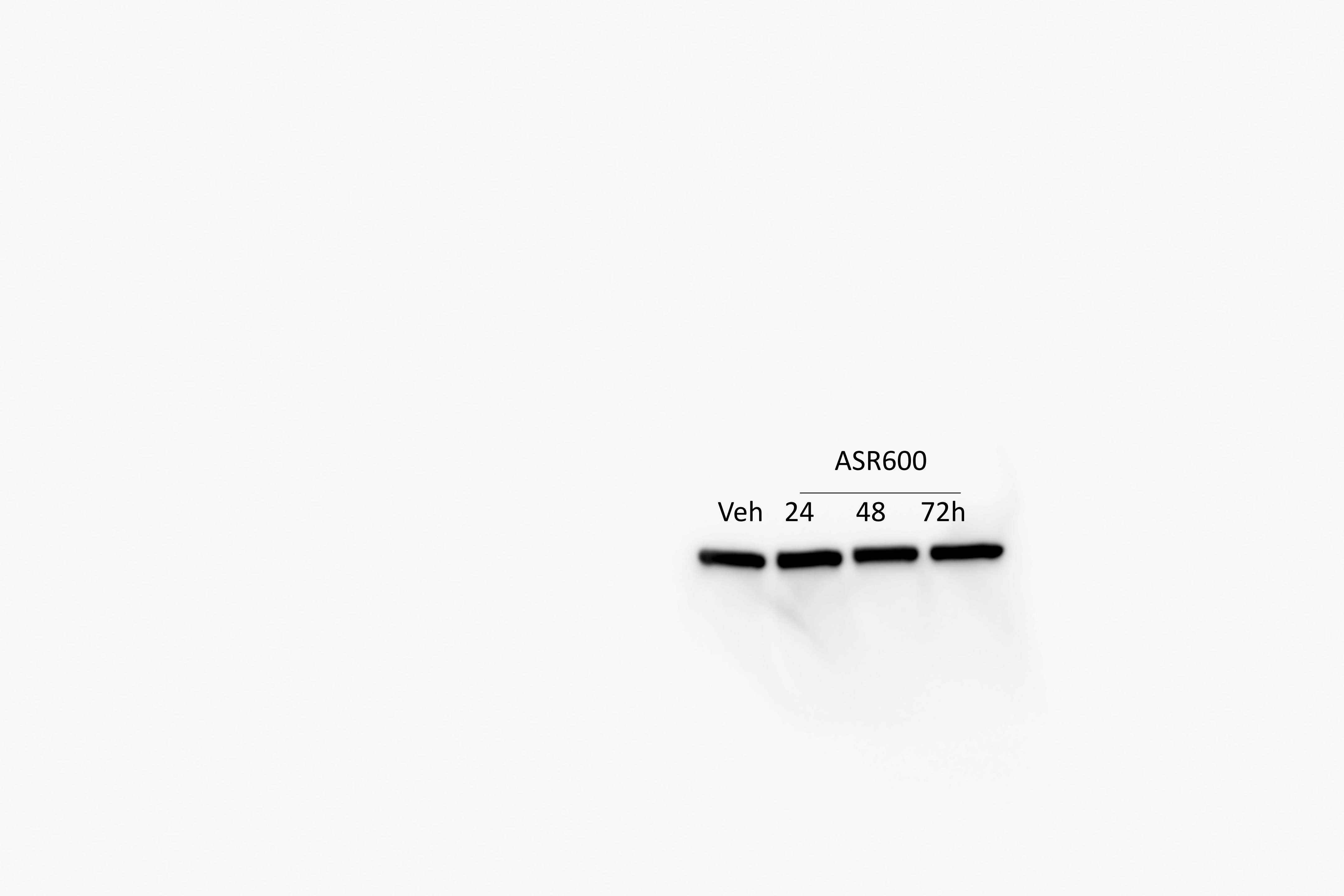

Fig.6b
mTOR

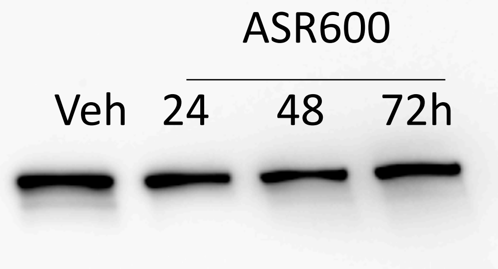

Fig.6b
mTOR_
Actin

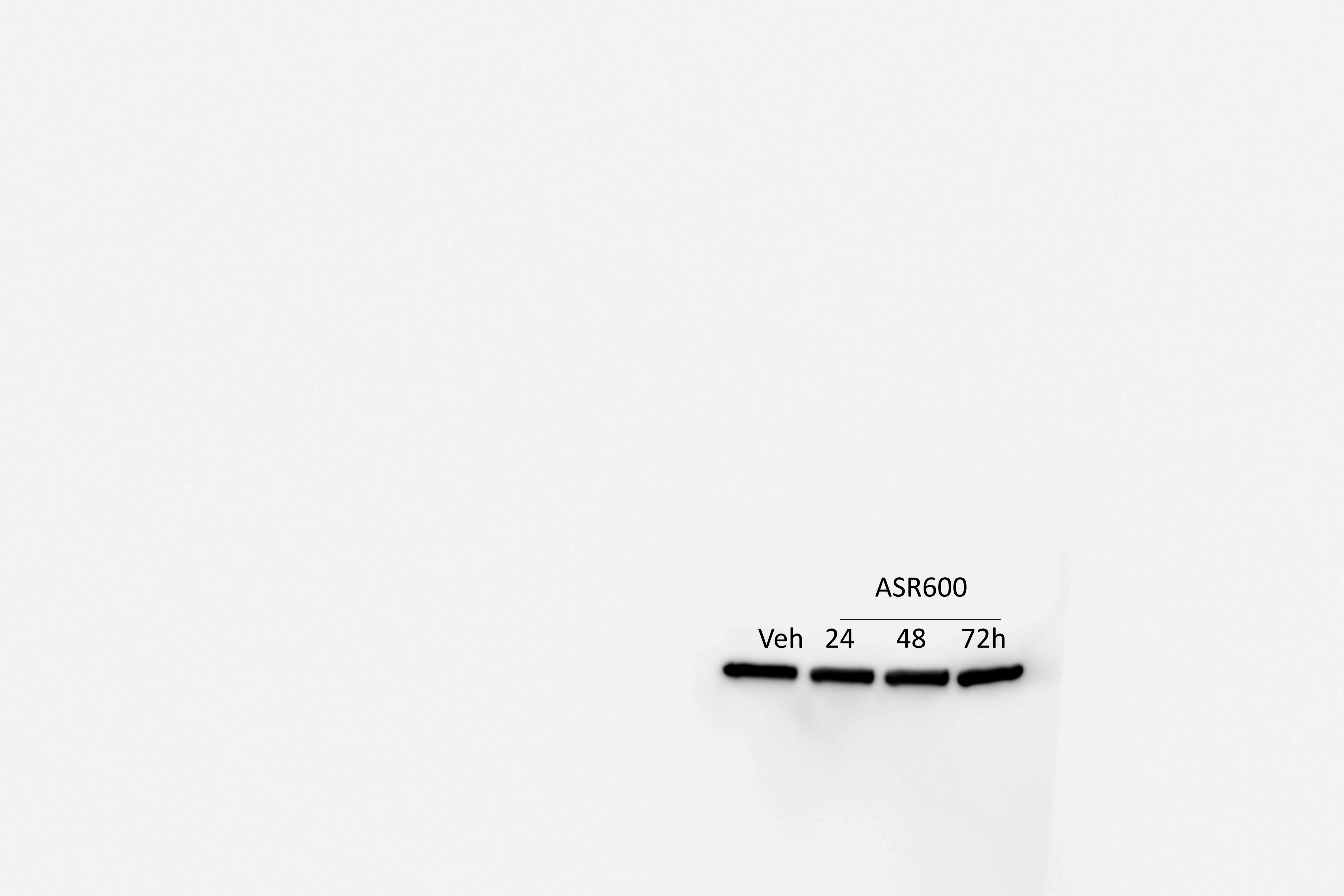

Fig.6b
pAKT

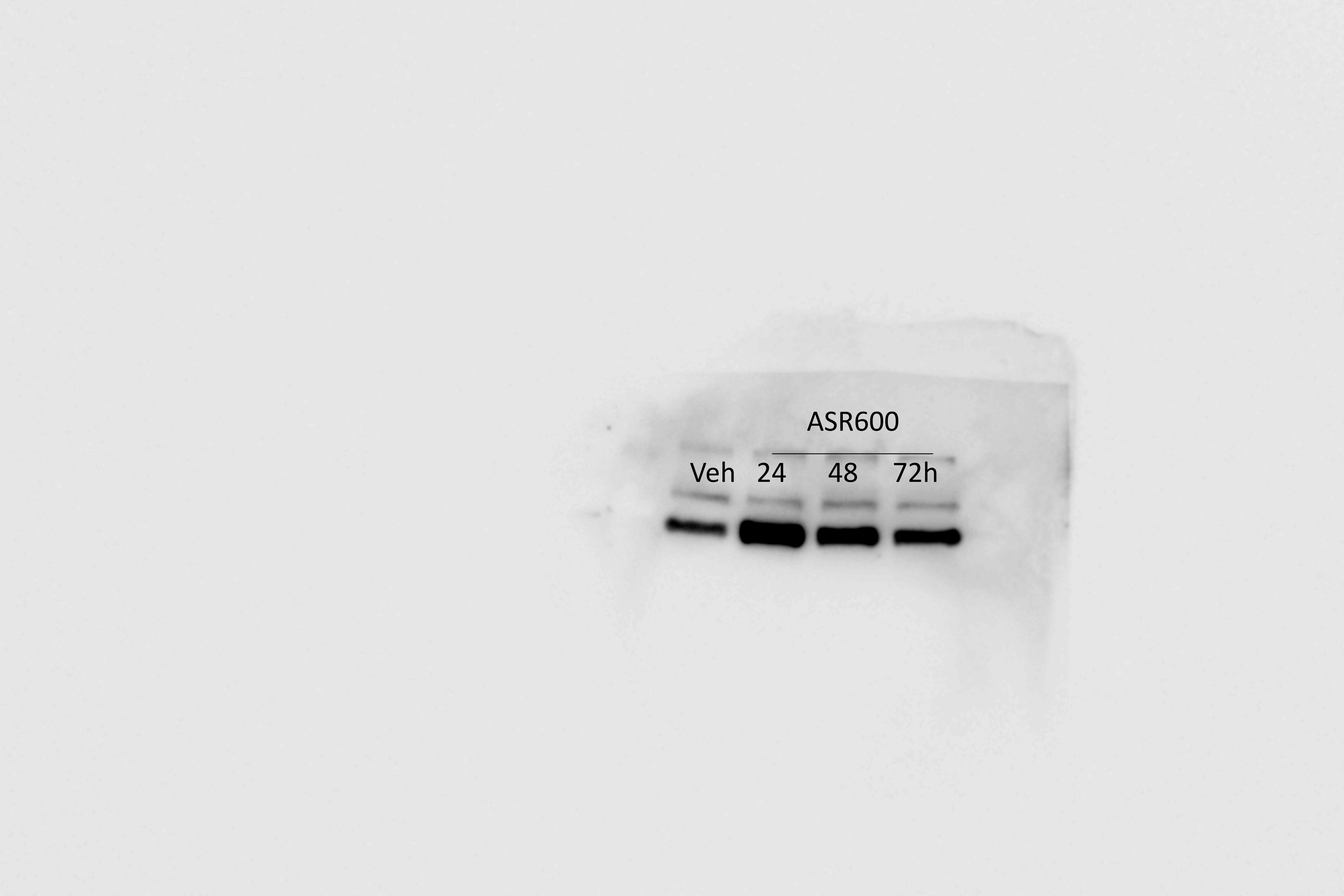

Fig.6b
AKT

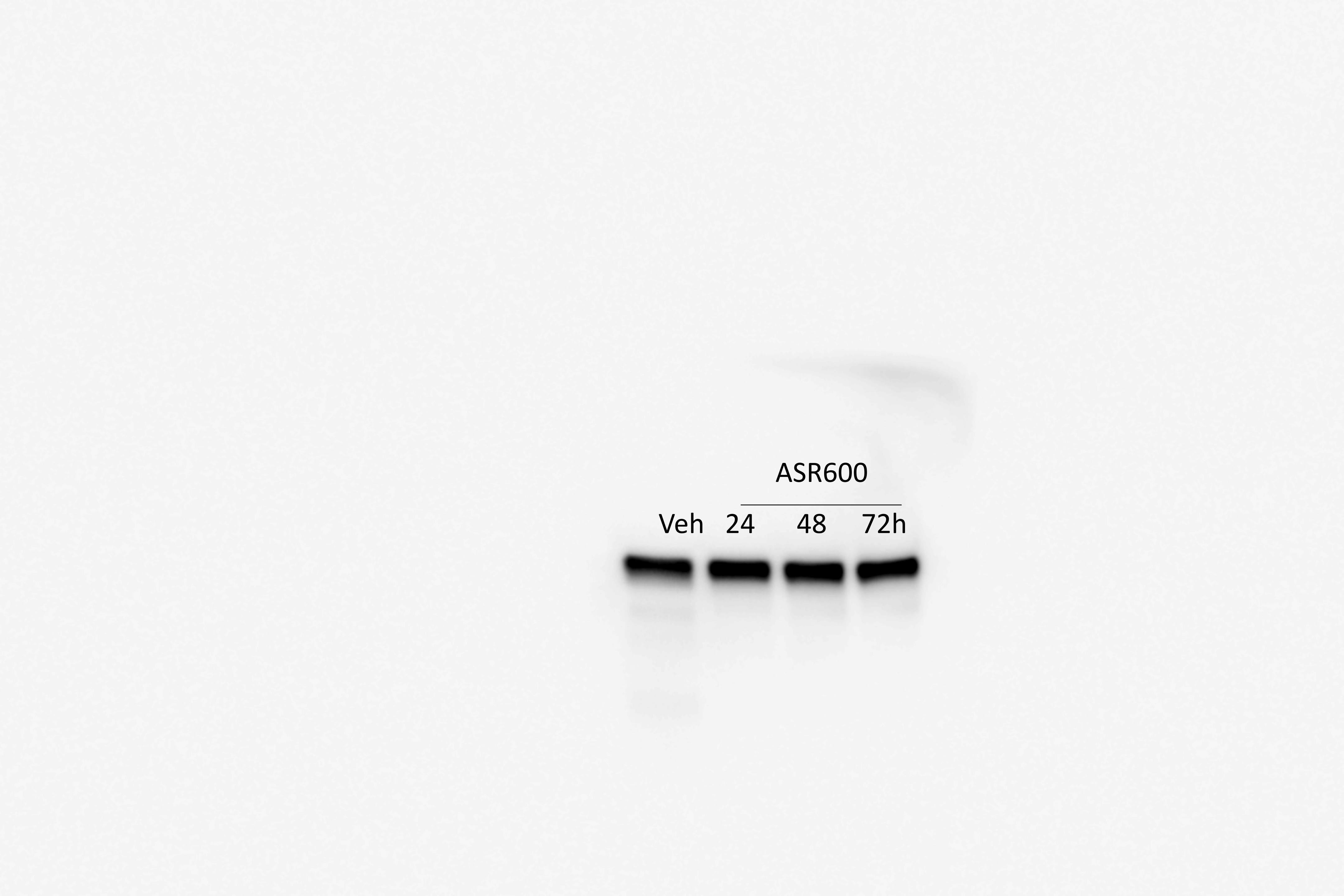

Fig.6b
pAKT_
Actin

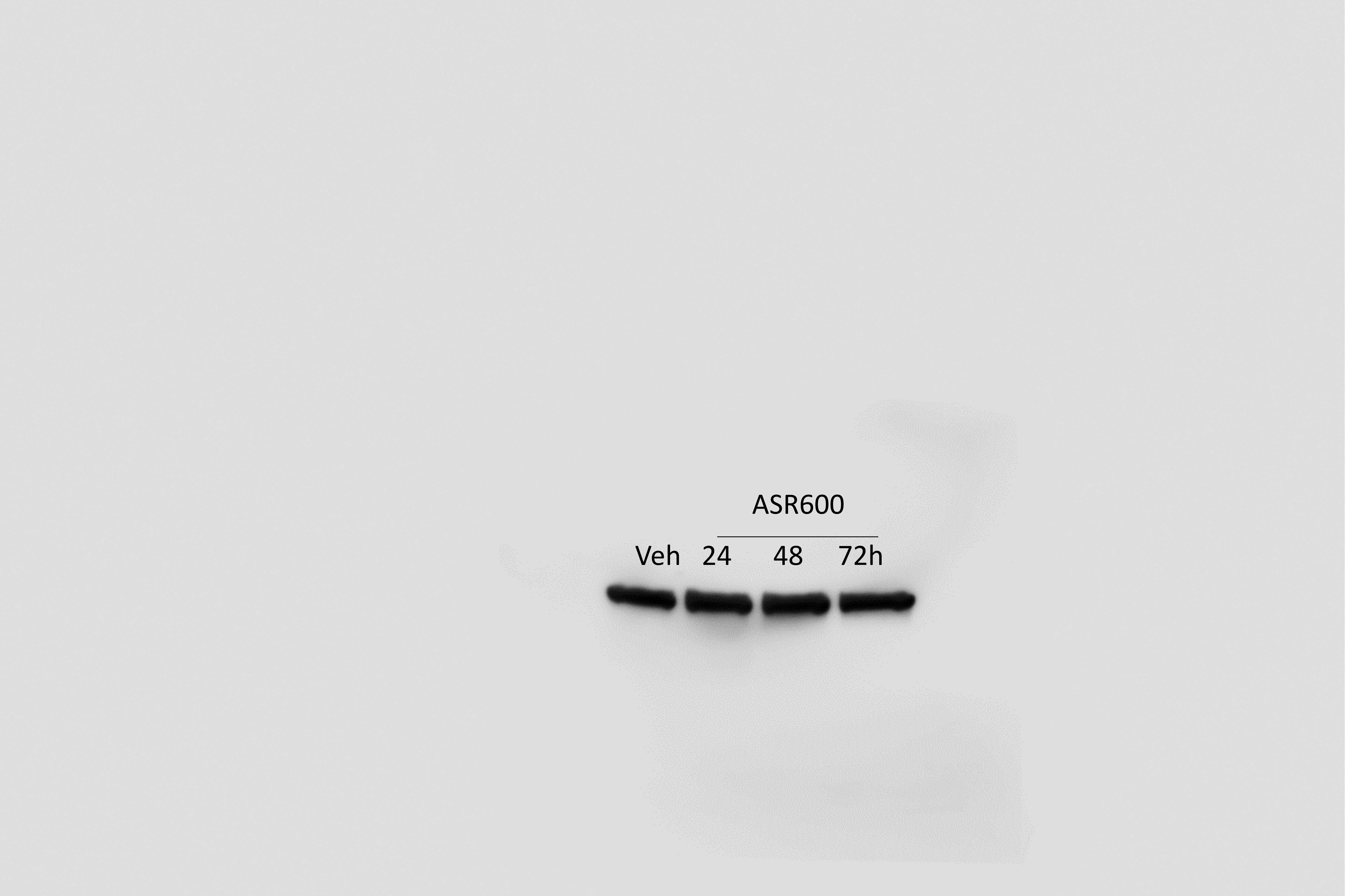

Fig.6c
ERα

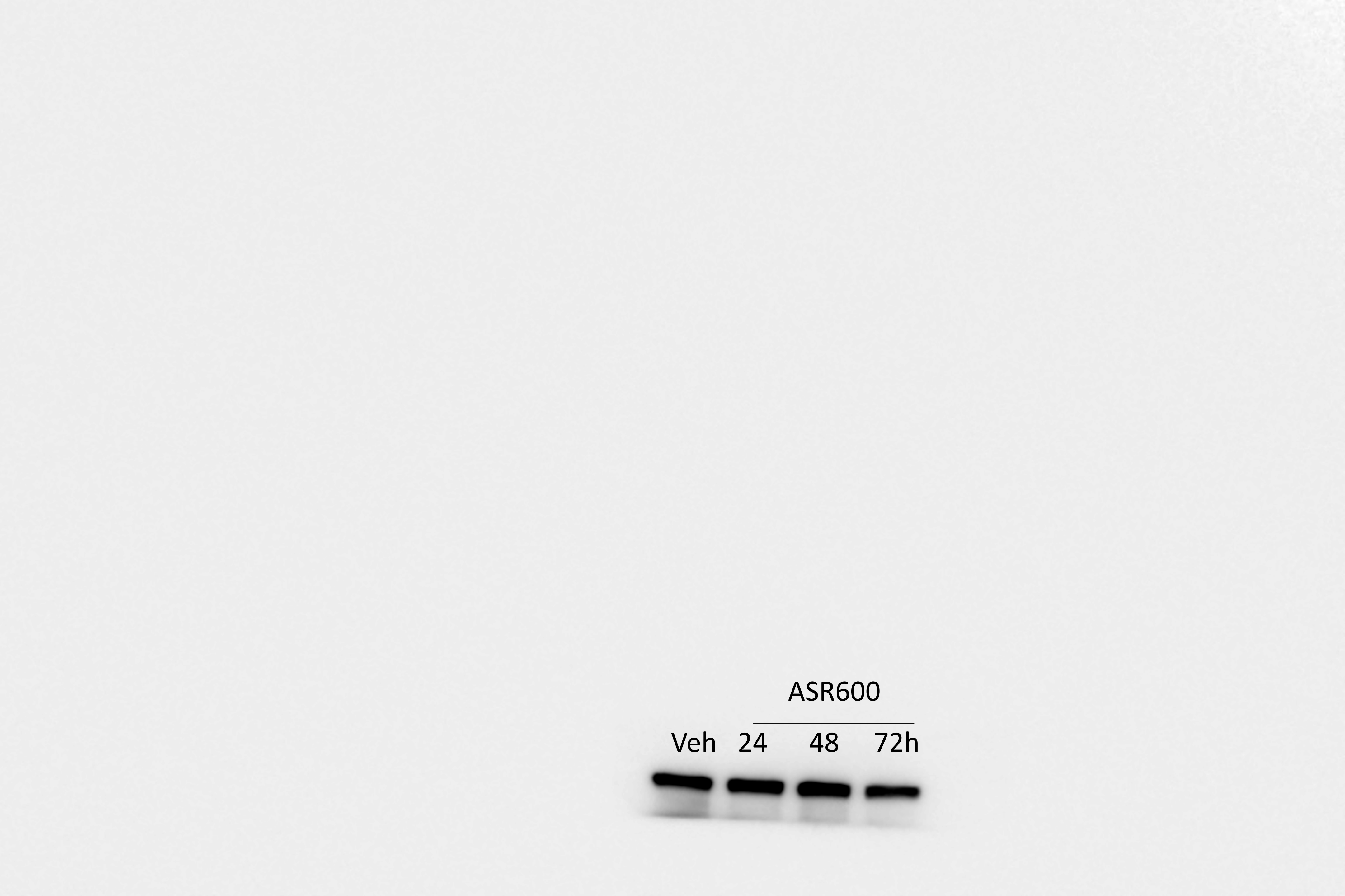

Fig.6c
ER_Actin

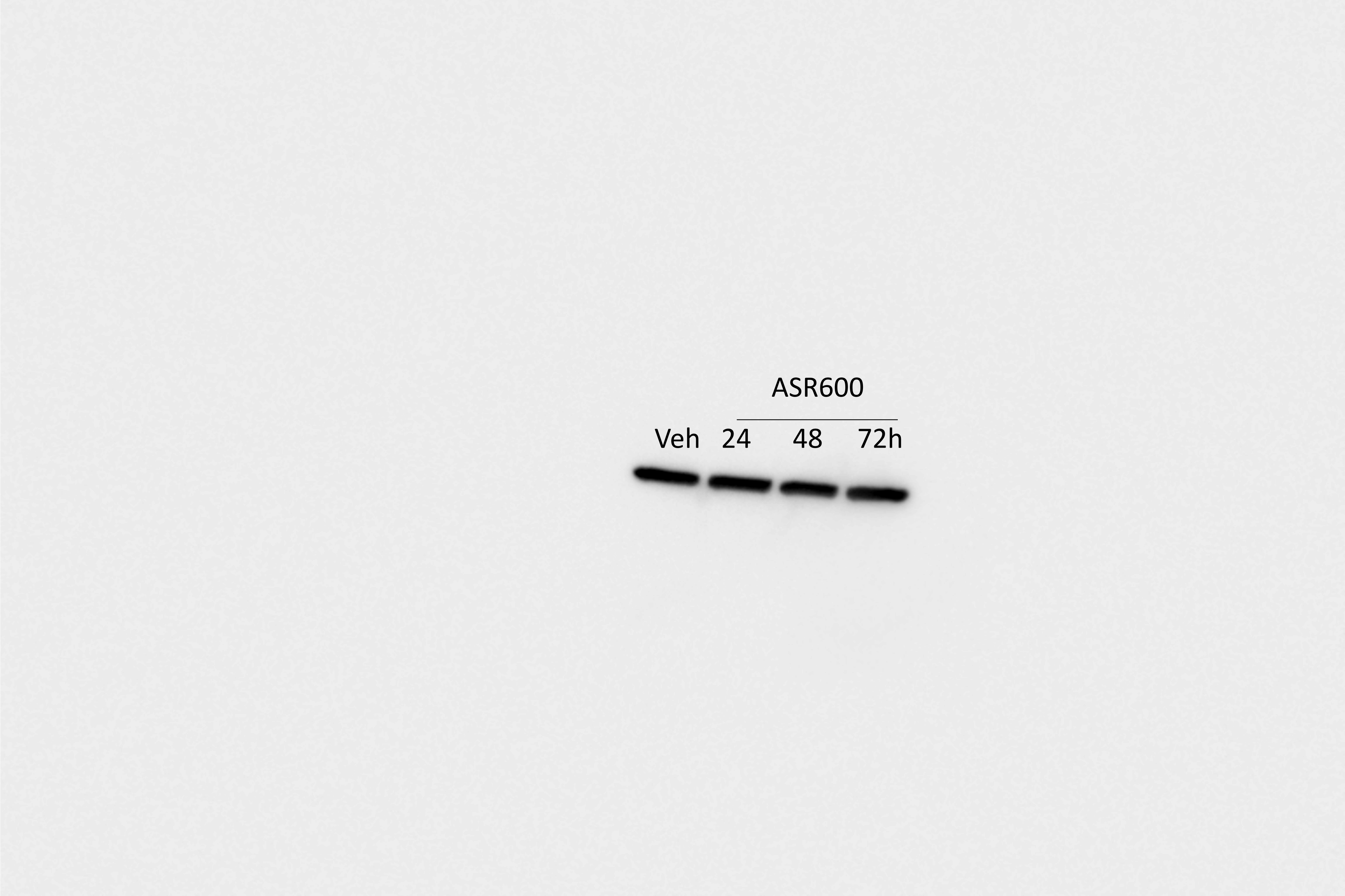

Fig.6c PR

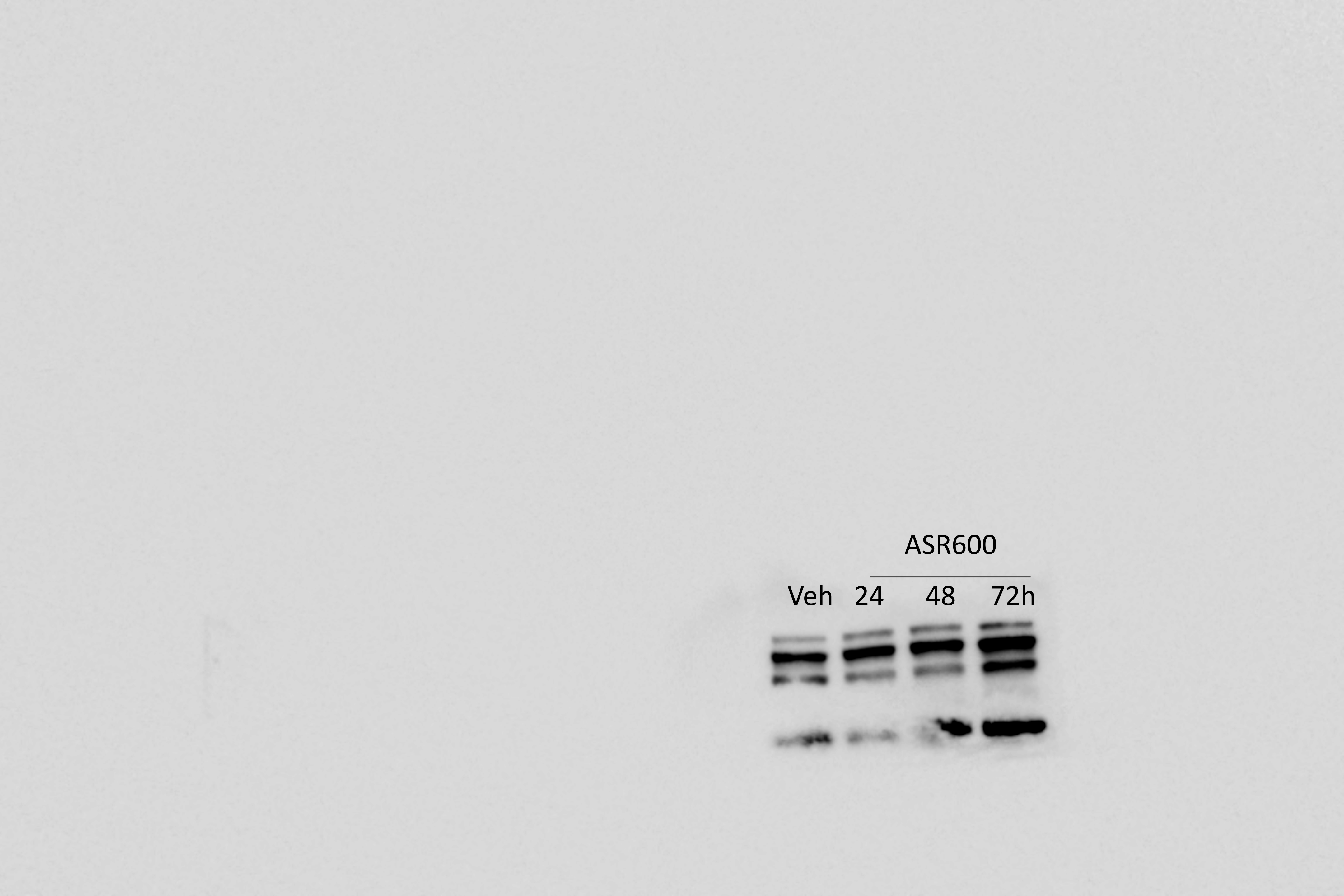

Fig.6c
PTEN

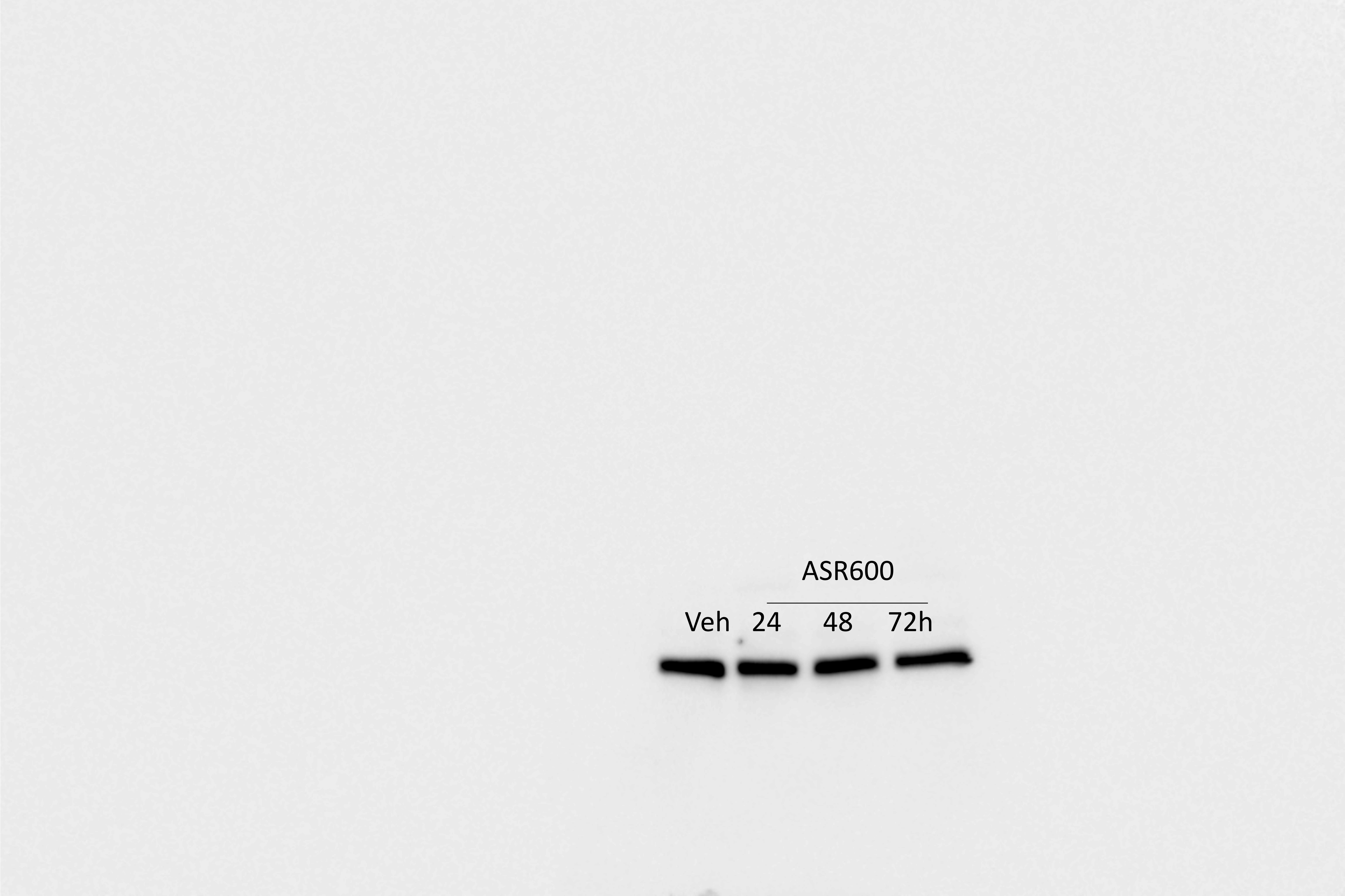

Fig.6c
Actin

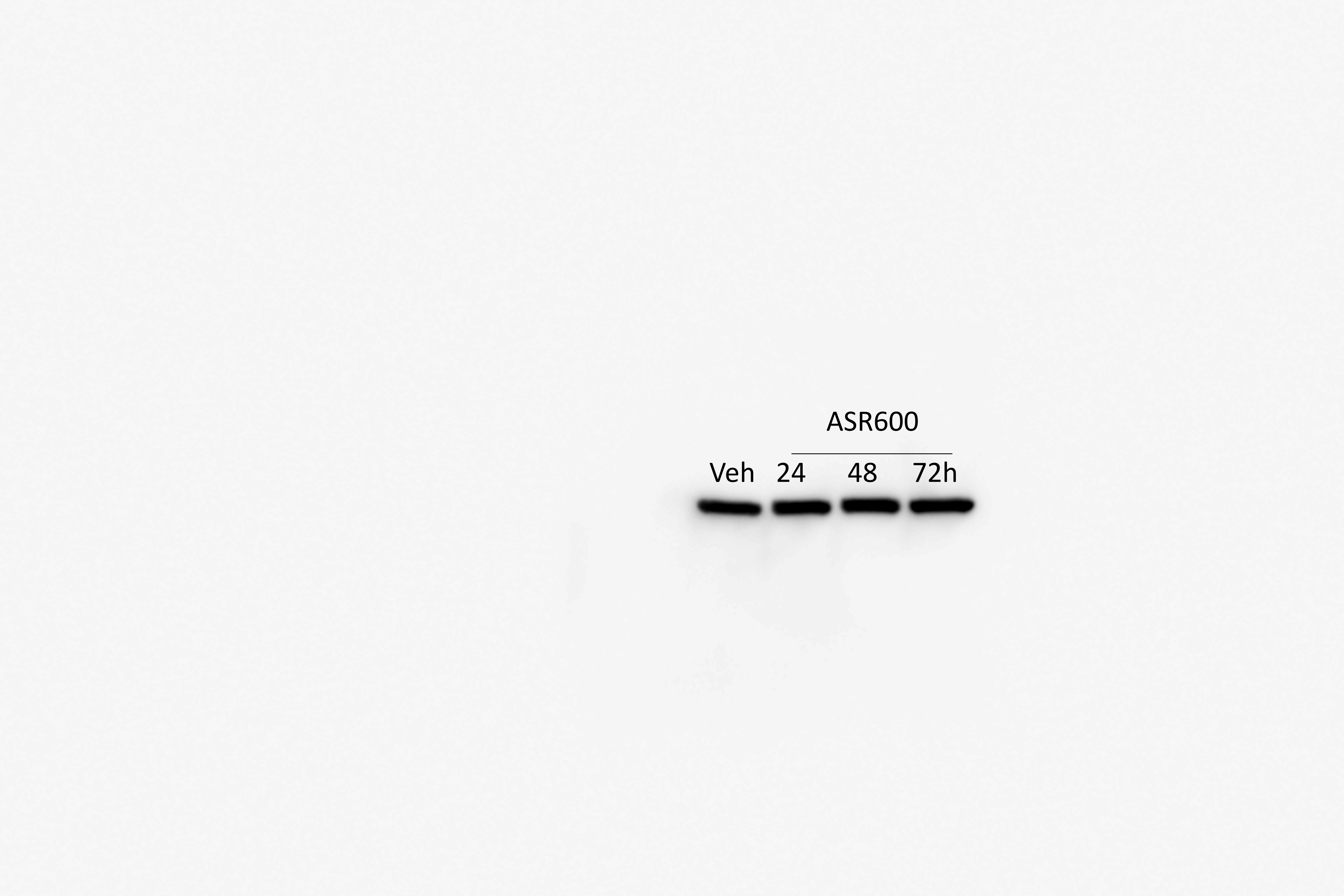

Supplement: Supplementary file 2 [file DataSheet3.PDF]
