# Supplementary material for: Urolithin A analog inhibits castration-resistant prostate cancer by targeting the androgen receptor and its variant, androgen receptor-variant 7
Source: Front Pharmacol. 2023 Mar 3;14:1137783. doi: 10.3389/fphar.2023.1137783 (PMC10020188; doi:10.3389/fphar.2023.1137783)
Supplement: Supplementary file 3 [file DataSheet1.PDF]

Supplementary Table 1. List of antibodies used in western blotting		
Antibody Name	Company	Dilution
AR-FL	Cell signaling (CST#5153)	1:1000
AR-V7	Abcam [EPR15656] (ab198394)	1:1000
PSA	Abcam (abcam#53774)	1:1000
AKT	Cell Signaling (CST#4691)	1:1000
$pAKT^{Ser473}$	Cell Signaling (CST#4060)	1:1000
PTEN	Abcam (abcam#31392)	1:1000
$pmTORSer^{2481}$	Cell Signaling (CST #5536)	1:1000
mTOR	Cell signaling (CST#2972)	1:1000
Erα	Cell signaling (CST#8644)	1:1000
PR	Cell signaling (CST#3176)	1:1000
Ubiquitin	Cell signaling (CST#3936)	1:1000
Lamin A	Cell signaling ((CST#3936)	1:1000
β-Actin	Cell signaling (CST#5125)	1:2000
Blocking condition: PVDF membranes are blocked with 5% non-fat milk.		
Primary antibodies are incubated overnight at 4°C.		
Secondary antibodies are incubated for one hour at room temperature .		
